# Genotype Imputation with Thousands of Genomes

**DOI:** 10.1534/g3.111.001198

**Published:** 2011-11-01

**Authors:** Bryan Howie, Jonathan Marchini, Matthew Stephens

**Affiliations:** * Department of Human Genetics and; † Department of Statistics, University of Chicago, Chicago, Illinois 60637, and; ‡ Department of Statistics, University of Oxford, Oxford OX1 3TG, United Kingdom

**Keywords:** GWAS, reference panel, haplotype, linkage disequilibrium, human

## Abstract

Genotype imputation is a statistical technique that is often used to increase the power and resolution of genetic association studies. Imputation methods work by using haplotype patterns in a reference panel to predict unobserved genotypes in a study dataset, and a number of approaches have been proposed for choosing subsets of reference haplotypes that will maximize accuracy in a given study population. These panel selection strategies become harder to apply and interpret as sequencing efforts like the 1000 Genomes Project produce larger and more diverse reference sets, which led us to develop an alternative framework. Our approach is built around a new approximation that uses local sequence similarity to choose a custom reference panel for each study haplotype in each region of the genome. This approximation makes it computationally efficient to use all available reference haplotypes, which allows us to bypass the panel selection step and to improve accuracy at low-frequency variants by capturing unexpected allele sharing among populations. Using data from HapMap 3, we show that our framework produces accurate results in a wide range of human populations. We also use data from the Malaria Genetic Epidemiology Network (MalariaGEN) to provide recommendations for imputation-based studies in Africa. We demonstrate that our approximation improves efficiency in large, sequence-based reference panels, and we discuss general computational strategies for modern reference datasets. Genome-wide association studies will soon be able to harness the power of thousands of reference genomes, and our work provides a practical way for investigators to use this rich information. New methodology from this study is implemented in the IMPUTE2 software package.

Genotype imputation is a well-established statistical technique for estimating unobserved genotypes in association studies (Browning 2008; Li *et al.* 2009; Marchini and Howie 2010). Imputation works by copying haplotype segments from a densely genotyped reference panel into individuals typed at a subset of the reference variants. In this way, genotypes can be estimated and tested for association at variants that were not assayed in a study. This approach can increase the power of a given study (Guan and Stephens 2008; Li *et al.* 2010; Marchini *et al.* 2007; Servin and Stephens 2007), find candidate susceptibility variants to guide fine-mapping (*e.g.* Liu *et al.* 2010), and facilitate meta-analyses that combine studies genotyped on different sets of variants (De Bakker *et al.* 2008; Zeggini and Ioannidis 2009).

Although the statistical methods for genotype imputation are now highly developed and widely used, there are still open questions in the field. Of these, one of the most pressing is how best to use rapidly accumulating reference datasets from around the world to impute into various human populations. Our goal in this paper is to develop a framework for achieving accurate and efficient imputation with current and future reference panels. We focus specifically on low-frequency variation (defined here as polymorphisms with MAFs from 0.5 to 5%) because interrogating such variants is a major aim of contemporary association studies (McCarthy *et al.* 2008; Stranger *et al.* 2011).

In the first generation of genome-wide association studies (GWAS), most imputation analyses used reference panels from Phase 2 of the International HapMap Project (The International HapMap Consortium 2005), which contain a total of 210 unrelated individuals with ancestry from West Africa, East Asia, and Europe. More recently, Phase 3 of the HapMap Project (The International HapMap Consortium 2010) increased the public reference set to over 1000 unrelated individuals sampled from 11 locations, including parts of the world that were poorly represented by HapMap 2. The 1000 Genomes Project (The 1000 Genomes Project Consortium 2010) is currently extending this resource by applying whole-genome shotgun sequencing to an even larger number of individuals (∼2500) sampled from a finer geographic grid (∼25 separate locations). Direct sequencing can identify variants not represented on the genotyping arrays that were used in the HapMap Project, so this reference set will include many more polymorphic sites than did previous imputation reference panels, with most of the new variants occurring at low population frequencies. As high-throughput genotyping and sequencing technologies continue to mature and decrease in cost, the worldwide collection of reference data will continue to grow.

There are numerous approaches for building imputation reference panels from publicly available resources. Many published GWAS simply used the HapMap 2 panel that most closely matched the ancestry of the study population; *e.g.* CEU for European populations and CHB+JPT for East Asian populations. This “best match” strategy produced useful results in several studies, but it can yield suboptimal accuracy with more diverse reference collections (The International HapMap Consortium 2010) or in studies with no clear reference matches (Huang *et al.* 2009a). A simple alternative is to use a “cosmopolitan” reference set that includes all available haplotypes, each of which is assigned an equal chance of being copied *a priori*. This approach produces relatively accurate results in a variety of human populations and has therefore been proposed as a good fallback choice when the optimal panel composition is unclear (Guan and Stephens 2008; Huang *et al.* 2009a; Li *et al.* 2010). Another class of methods tries to maximize accuracy by weighting reference panels through cross-validation (Huang *et al.* 2009a) or ancestry estimation (Egyud *et al.* 2009; Pasaniuc *et al.* 2010); the Pasaniuc *et al.* approach differs from the others in that it uses *local* ancestry estimates to provide customized reference weights for each study individual. As an alternative, Jostins *et al.* (2011) suggested balancing accuracy and computation by using reference panels that “approximately cluster” with the study individuals on a plot of principal components (PC) that capture genetic ancestry.

The standard way to impute genotypes in a GWAS is to apply one of these panel selection schemes and then pass the indicated haplotypes to an imputation method. Modern reference datasets pose a couple of challenges to this paradigm. First, many panel selection strategies become harder to execute or interpret as reference data are sampled from more populations. For example, the systematic HapMap 2 cross-validations from Huang *et al.* (2009a) would be more difficult to implement with additional panels, and it is harder to make clear demarcations on a plot of PCs [as recommended by Jostins *et al.* (2011)] as more populations are included. More importantly, larger reference panels increase the computational burden of imputation, which may compel some investigators to use smaller panels at the cost of imputation accuracy and association power. Methodological developments like “pre-phasing” (which we address in the *Discussion*) can alleviate this concern by speeding up imputation, but existing methods still need substantial computing power to handle reference sets with thousands of haplotypes.

Our work was motivated by the idea that growing reference datasets need not make panel selection more difficult or force tradeoffs between imputation speed and accuracy; in principle, larger and more diverse reference collections could actually make it easier to identify haplotype sharing with simple models, thereby making imputation faster *and* more accurate. Kong *et al.* (2008) demonstrated this point in a large sample from a founder population, where they found that genotypes could be accurately phased and imputed using simple identity-by-state (IBS) calculations to identify shared haplotype segments. Howie *et al.* (2009) introduced a related idea for phasing smaller samples from outbred populations, and we wanted to see whether a similar approach could help impute genotypes from modern reference panels.

On the basis of our findings, we propose an imputation framework with two basic components: (i) a cosmopolitan reference panel and (ii) a new algorithmic approximation that maintains the accuracy of large, diverse panels while controlling computational costs. We use a cosmopolitan panel for its combination of simplicity and accuracy: past studies have shown that such panels produce similar accuracy to those chosen by more sophisticated schemes (Guan and Stephens 2008; Huang *et al.* 2009a; Li *et al.* 2010) and that they can improve accuracy at low-frequency variants (Jostins *et al.* 2011; Marchini and Howie 2010). Our approximation is based on the idea that, within a limited genomic region, allelic consistency between study and reference individuals can be used to quickly rule out unhelpful reference haplotypes, thereby making imputation faster without sacrificing accuracy. In our framework, this approximation reduces the full reference set to a custom panel for each study haplotype in each part of the genome. The approximation is similar to the one that drives the phasing algorithm of IMPUTE version 2 (IMPUTE2; Howie *et al.* 2009), and we have implemented it in the same software package.

We evaluate our framework by running extensive cross-validations in HapMap 3 and in African data from the MalariaGEN Project (Malaria Genomic Epidemiology Network 2008). With a more detailed panel selection scheme as a benchmark, we find that our approach produces high imputation accuracy in all populations considered, with the greatest benefits at low-frequency variants. We also use simulated data to show that our approximation substantially reduces the computational cost of adding haplotypes to a reference set. We further demonstrate that an implementation of our framework is faster and more accurate than another leading method (Beagle; Browning and Browning 2009) when imputing from large, sequence-based reference panels. On the basis of our results, we discuss general computational strategies for balancing efficiency and accuracy, and we explain how our methodology can be combined with other techniques for speeding up imputation.

We have tied together numerous threads from the literature to create a coherent, efficient, and accurate framework for imputing genotypes from modern reference datasets. Imputation-based GWAS are beginning to harness the power of thousands of reference genomes, and we expect that the practical solutions provided here will help investigators make the most of these rich genetic resources.

## Materials and Methods

### IMPUTE2 algorithm

We begin by describing the basic IMPUTE2 algorithm, which will be retained in this work with some modifications. Full details of the original algorithm are available in Howie *et al.* (2009). Although that paper addressed the use of multiple reference panels typed on different SNP sets (“Scenario B” in their terminology), for simplicity we will focus on the situation where the reference haplotypes are all defined on the same SNPs (“Scenario A”). Although we do not discuss Scenario B in this paper, the ideas presented here are easily extended to that setting.

IMPUTE2 uses a Markov chain Monte Carlo (MCMC) algorithm that alternates between phasing typed SNPs and imputing untyped SNPs. Each MCMC iteration includes two steps:

1. Sample a new phase configuration for each study individual, using information from other study individuals and reference panel haplotypes at SNPs typed in the study.
2. Given the newly sampled haplotypes for all study individuals, treat each haplotype as independent (conditional on the reference panel) and analytically impute the alleles at untyped SNPs.

This MCMC algorithm is run for a number of iterations (typically 30, including 10 burn-in iterations), then the probabilities from Step 2 are averaged across iterations to produce marginal posterior genotype probabilities at each untyped SNP.

The phasing and imputation calculations are driven by the hidden Markov model (HMM) of Li and Stephens (2003). This model can be used to update an individual's haplotypes by constructing them as “imperfect mosaic” copies of a set of template haplotypes. In the simplest case, the templates would include all reference+study haplotypes (minus the pair being updated) in Step 1 and all reference haplotypes in Step 2.

To reduce the computational burden of Step 1, Howie *et al.* (2009) introduced an approximation that restricts each phasing update to a set of *k* template haplotypes, which are chosen separately for each individual at each iteration; the other templates are implicitly assigned copying probabilities of zero. The *k* templates are chosen by computing Hamming distances between an individual's current sampled haplotypes and each possible template haplotype. We refer to the *k* templates with the smallest distances as “surrogate family members” because they (ideally) share recent ancestry with the study individual. [These haplotypes were called “informed conditioning states” in the Howie *et al.* (2009) paper and early versions of the IMPUTE2 documentation. We now prefer the nomenclature used here because of the approximation’s relationship to the “surrogate parent” phasing method of Kong *et al.* (2008).]

Howie *et al.* (2009) used the surrogate family approximation to speed up the phasing updates in IMPUTE2 (Step 1 of the MCMC algorithm described above), but they used all available reference haplotypes for the imputation updates (Step 2). To make computation faster in large, ancestrally diverse reference panels, we now extend the approximation to imputation updates.

To decide which reference haplotypes to copy at a particular point in an IMPUTE2 run, we add an extra step between Steps 1 and 2. After individual *i* has sampled a new haplotype pair in Step 1, we calculate the Hamming distance from each of these haplotypes to each of the reference haplotypes, using only the overlapping SNPs. Then, separately for each of individual *i* 's haplotypes, we perform Step 2 (haploid imputation of untyped alleles) using only the *k_hap_* nearest reference haplotypes as templates. This procedure is not guaranteed to identify *k_hap_* unique haplotypes as multiple haplotypes near the *k_hap_* cutoff may have the same Hamming distance. In these situations, we select a random subset of the boundary haplotypes to produce a reference panel with *k_hap_* states. Intuitively, our approach corresponds to imputing each study haplotype from a “custom” reference panel containing close genealogical neighbors. We generally choose larger values for *k_hap_* than for *k* because phasing updates require evaluation of *k*^2^/2 HMM states per individual per SNP, whereas imputation updates require evaluation of only *k_hap_* states.

### HapMap 3 cross-validation experiments

To assess the accuracy of genotype imputation from reference panels of diverse ancestry, we performed leave-one-out cross-validations in data from HapMap Phase 3. The HapMap 3 paper (The International HapMap Consortium 2010) includes a number of similar comparisons, which use a series of carefully controlled experiments to show how population ancestry in the reference and study data affects imputation accuracy. By contrast, our main goal is to validate a general strategy for using ancestrally diverse reference data. One way to view this distinction is that their experiments provide information about the kinds of reference data one might want to collect to improve imputation accuracy in a given population, whereas our experiments illustrate a strategy for getting good results from whichever data are available. To the extent that we consider how reference panels of different ancestries affect imputation accuracy, the main point is to guide intuition about why the overall strategy works.

All of our experiments were based on phased haplotypes from HapMap 3, release 2, in NCBI Build 36 coordinates. HapMap 3 includes samples from 11 analysis panels, which are listed in Table 1. After some minor processing (which is described in the Table 1 legend), there was a total of 1011 unrelated individuals from 10 panels in this dataset. Recent work on the HapMap data has revealed a small number of close relatives among these putative unrelateds (Pemberton *et al.* 2010). We conducted the analyses for this study before those relationships were revealed, but we do not anticipate that removing the related individuals would have a major effect on our results or conclusions.

**Table 1:** HapMap 3 panels used for cross-validation

Panel ID	Panel Description	Number of Unrelated Individuals*^a^*
ASW	African ancestry in Southwest USA	63
CEU	Utah residents with Northern and Western European ancestry from the CEPH collection	117
CHB*^b^*	Han Chinese in Beijing, China	84
CHD	Chinese in Metropolitan Denver, Colorado	85
GIH	Gujarati Indians in Houston, Texas	88
JPT*^b^*	Japanese in Tokyo, Japan	86
LWK	Luhya in Webuye, Kenya	90
MKK	Maasai in Kinyawa, Kenya	143
MXL	Mexican ancestry in Los Angeles, California	52
TSI	Toscani in Italia	88
YRI	Yoruba in Ibadan, Nigeria	115
	All panels	1011

a In panels that included trios (ASW, CEU, MXL, MKK, and YRI), we retained the trio parents as “unrelated” individuals. In panels that included parent-child duos (ASW, CEU, MXL, and YRI), we retained the observed duo parent and the inferred transmitted haplotype from the unobserved duo parent, yielding three “unrelated” haplotypes per duo; we then paired the inferred transmitted haplotypes at random to create diploid pseudo-individuals.

b We combined the CHB and JPT panels into a single CHB+JPT panel with 170 individuals for all of the analyses in this paper.

The HapMap 3 samples were genotyped on both the Affymetrix SNP 6.0 platform and the Illumina 1M Human platform. For our cross-validations, we masked the SNPs not typed on the Affymetrix platform in one individual at a time, then imputed the masked genotypes from the haplotypes of other HapMap 3 individuals; details about which individuals were included in the reference panel are provided below. We also masked the phase of the observed genotypes in the individual being imputed. For these experiments, we used all of the HapMap 3 SNPs on chromosome 20, which led to 16,606 non-Affymetrix SNPs being imputed from 19,650 Affymetrix SNPs. We also repeated the experiments after reversing the roles of the SNP platforms, with results shown in File S3.

Once every individual in a panel had been masked and imputed, we assessed accuracy at each SNP as the squared Pearson correlation (*R*^2^) between the masked genotypes, which take values in {0,1,2}, and the imputed allele dosages (also known as posterior mean genotypes), which take values in [0,2]. The allele dosage is defined for each genotype G as $\sum_{x=0}^{2}Pr(G=x)*x$, where *Pr* (*G* = *x*) is a marginal posterior probability generated by an imputation method. Once the correlation *R*^2^ had been measured for every masked SNP, we calculated the mean *R*^2^ across SNPs and reported this as a scalar summary of imputation accuracy in that cross-validation experiment. In rare situations, the correlation at a SNP was undefined because the imputation produced identical allele dosages for all individuals. In these cases, we set *R*^2^ = 0 to capture the intuition that there would be no power to detect an effect at such SNPs. We note that the HapMap 3 samples were phased together in continental groups, which implies that the absolute accuracies in this experiment may be slightly optimistic. However, our main focus is on the *relative* accuracy of different reference panel configurations, so the non-independence of cross-validation samples is not a meaningful shortcoming.

We repeated this experiment for each of the 10 HapMap 3 panels in Table 1 under various conditions. To understand the benefits and drawbacks of different reference panel compositions, we sequentially added HapMap 3 panels to the reference set and re-imputed after each addition. Adding the panels in all possible orders is combinatorically daunting and of dubious interpretive value, and we preferred to have an objective ordering, so we added panels in order of increasing pairwise *F_ST_* to the cross-validation panel. We calculated *F_ST_* between each pair of panels as the average across all HapMap 3 SNPs on the autosomes. The panel orderings induced by this criterion are shown in File S1. We also considered different orderings – *e.g.* adding panels as dictated by a greedy algorithm based on cross-validation accuracy – but these did not change our qualitative results (data not shown).

Up to this point, we have described 10 separate cross-validation experiments for each HapMap 3 panel: a cross-validation within the panel of interest, followed by 9 additional experiments with successively more inclusive reference panels. We also wanted to assess the sensitivity of the inference to the *k_hap_* parameter in IMPUTE2, so we repeated each of these experiments across a grid of *k_hap_* values: 15, 30, 60, 90, 120, 150, 200, 250, 300, 350, 400, 500, 600, 700, 800, 900, 1000, 1100, 1200, 1300, 1400, 1500, 1600, 1700, 1800, 1900, and 2020 (which is the total number of HapMap 3 haplotypes, 2022, minus the two haplotypes of the masked individual). By definition, *k_hap_* cannot exceed the number of haplotypes in the reference panel, so each reference configuration used only the values that were consistent with its panel size. Each configuration also used a special *k_hap_* value that was set to the total number of reference haplotypes in that experiment, since this value seldom fell on a grid point.

We performed these experiments with IMPUTE version 2.1.2 under the following settings: *k* = 80 (tuning parameter for phasing updates), *iter* = 30 (total number of MCMC iterations), *burnin* = 10 (number of *iter* to discard as burn-in), *hap_spec_fam* (flag to make the program choose a custom reference panel for each study haplotype), and *Ne* = 20000. The *Ne* parameter represents the effective size of the population being analyzed, and it is used to scale the recombination rates in the imputation HMM. It may seem odd that we use a single *Ne* value in populations that clearly have different effective sizes, but our pilot experiments showed that IMPUTE2 is largely insensitive to this parameter and that 20000 is a good universal value (data not shown). The approximations underlying the *k* and *k_hap_* parameters are modeled on local genealogies with limited recombination, so we split chromosome 20 into nonoverlapping 5-Mb chunks for analysis, with a 250-kb buffer region on each side to prevent edge effects (this is a default setting in IMPUTE2).

It is useful to run a separate imputation method as an external benchmark. We chose to compare against Beagle (Browning and Browning 2009) because past results showed that it could be competitive on a dataset of this scale (Browning and Browning 2009; Howie *et al.* 2009; Jostins *et al.* 2011). Beagle has already been compared with IMPUTE2 in a large, well-matched reference panel of European ancestry (Howie *et al.* 2009), so to simplify the presentation we applied it only to the cosmopolitan HapMap 3 reference panels. We used Beagle version 3.0.2 with default settings for all experiments presented here. To facilitate parallel computation, we ran Beagle on the same 5-Mb chromosome chunks (with buffers) that were used by IMPUTE2.

We also attempted to use a “coalescent-based” method for choosing custom reference panels (Pasaniuc *et al.* 2010), but we could not get it to produce accurate results on our data; see File S6 for details. We omitted another leading imputation method, MaCH (Li *et al.* 2010), because it was not computationally feasible with the full HapMap 3 panel at the time of these experiments. Both IMPUTE2 and MaCH have recently been made more efficient through “pre-phasing” of GWAS genotypes (the MaCH implementation is called “minimac”). While we did not evaluate these approaches here, we have found that pre-phasing is complementary to our *k_hap_* approximation in preliminary experiments, as we explain in the *Discussion*. All else being equal, we would expect minimac to achieve similar accuracy to IMPUTE2 since both methods are based on the Li and Stephens (2003) model of DNA sequence variation, although further work may be needed to compare these methods in various contexts.

### MalariaGEN cross-validation experiments

To evaluate strategies for reference panel construction in African populations, we performed cross-validation experiments in genotypes that were kindly provided by the Malaria Genetic Epidemiology Network (MalariaGEN; Malaria Genomic Epidemiology Network 2008). The data we used were collected by MalariaGEN investigators for genome-wide association studies of human resistance or susceptibility to severe malaria. The individuals in these datasets were recruited at medical centers in the Gambia and Ghana; we henceforth refer to these samples by the tags GMB and GHN, respectively, with the understanding that they may not represent the full spectrum of genetic diversity in the countries of origin. Further details about the study recruitment are available at www.malariagen.net.

Each dataset consists of trios (658 from GMB and 608 from GHN) that were ascertained via proband children diagnosed with malaria in hospitals. The members of each trio were genotyped on the Illumina 650Y array, with the genotypes subjected to standard quality control procedures and phased by Beagle with trio information. MalariaGEN carried out the genotyping and data processing, then provided us with inferred haplotypes for the trio parents (1316 GMB individuals and 1216 GHN individuals) in NCBI Build 36 coordinates.

As in the HapMap 3 comparisons, our main goal in designing a cross-validation experiment was to provide guidance on how to use existing and future imputation reference panels. We chose to focus on the GMB panel because genotype imputation has previously been evaluated in a Gambian GWAS (Jallow *et al.* 2009) and because the 1000 Genomes Project is planning to sequence a set of individuals from the Gambia.

To mimic the planned 1000 Genomes Gambian dataset, we randomly allocated 100 GMB individuals to a reference panel. We also formed a reference panel from 100 randomly chosen GHN individuals. Ghana is located between Nigeria (the source of the HapMap YRI panel) and the Gambia on the Atlantic coast of Africa, so this panel contains reference haplotypes sampled nearer to the location of interest than those in the HapMap data.

We used the remaining 1216 GMB individuals as a validation set to model imputation into a Gambian GWAS. We imputed the GMB genotypes from a series of reference panels: all 2022 HapMap 3 (HM3) haplotypes; 200 GMB haplotypes (GMB); 200 GMB haplotypes plus 200 GHN haplotypes (GMB+GHN); and a combined set containing all 2422 GMB, GHN, and HM3 haplotypes (GMB+GHN+HM3).

For each reference panel, we masked and imputed every 25^th^ SNP in the GMB validation set, then repeated this analysis in a sliding window so that every genotyped SNP was imputed exactly once. To mimic a GWAS of unrelated individuals, we treated the nonmasked genotypes in the GMB validation set as unphased. We masked and imputed all available Illumina 650Y SNPs on chromosomes 20 and 11 (we added chromosome 11 to raise the counts of low-frequency SNPs, which are underrepresented in this dataset), except for those that were not typed in or had allele conflicts with HapMap 3, yielding a total of 40,300 SNPs for imputation. As in the HapMap 3 cross-validations, we split the chromosomes into non-overlapping 5-Mb regions to speed up the analysis and support IMPUTE2’s computational approximations.

We also wanted to compare against Beagle on reference panels of primarily African ancestry, so we generated another reference panel called “HM3.afr” that included the HapMap3 haplotypes from the ASW, LWK, MKK, and YRI panels (822 haplotypes). We then used IMPUTE2 and Beagle to impute genotypes in the 1216 Gambians based on two reference panels: GMB (200 haplotypes) and GMB+GHN+HM3.afr (1222 haplotypes). We masked the SNPs as described above, except that this time we masked every 13^th^ SNP so that Beagle would treat the data as it would a standard GWAS analysis. (Beagle invokes a different model-fitting strategy when fewer than 7% of the genotypes are missing from the study dataset.)

### Computational benchmarking

To produce computational benchmarks in a realistic imputation scenario, we simulated data that model the large, ancestrally diverse reference panel that is being generated by the 1000 Genomes Project. Our simulations were based on the sfs_code program (Hernandez 2008), which uses a pre-specified demographic model (typically obtained from unbiased site frequency spectra) and DNA sequence annotations to drive a forward simulation that models the effects of genetic drift and natural selection on a population of chromosomes. Ryan Hernandez kindly provided us with the output of an sfs_code run that used a joint demographic model of three HapMap panels (CEU, CHB, and YRI) on chromosome 17p12 (a 4.7-Mb region). At the end of the forward simulation, the program sampled 10,000 haplotypes from each of the three populations. These haplotypes do not capture the full demographic complexity of the 1000 Genomes sample set, but the simulation does provide realistic DNA sequence data for three major sources of human genetic variation.

Given these simulated sequences, we sought to create imputation reference panels that would capture features of the anticipated 1000 Genomes panels. We mirrored the overall size of the 1000 Genomes reference set by sampling a panel of 1600 chromosomes from each population, which yielded a total of 4800 chromosomes worldwide, just under the 1000 Genomes target of 5000. The genome-wide sequencing module of the 1000 Genomes Project is based on a low-coverage design, so a certain fraction of low-frequency variants will be missed in the real data. To mimic this ascertainment process, we used power calculations from the 1000 Genomes pilot paper (The 1000 Genomes Project Consortium 2010) to determine the chances of discovering SNPs with different numbers of variant allele copies. The discovery probabilities are shown in Table S2; we applied them separately in each set of 1600 reference chromosomes, under the assumption that true SNPs are discovered (or not) independently of each other. Conditional on a SNP being discovered in any panel, we assumed it was genotyped perfectly in all three panels. This is a reasonable assumption for a benchmarking experiment because sporadic genotyping errors are unlikely to have a noticeable effect on a program’s computational burden.

Having simulated a reference panel, we then simulated a GWAS dataset as the target for imputation. Of the 8400 CEU-like haplotypes from sfs_code that were not included in the reference set, we selected 2000 and randomly paired them to create a GWAS sample of 1000 individuals. We thinned this dataset to the approximate SNP density and MAF distribution observed in real Affymetrix 500k data on a set of British controls [the 1958 Birth Cohort of the Wellcome Trust Case Control Consortium (2007)].

We imputed our simulated GWAS dataset from two different reference panels: a “Cosmopolitan” panel containing the full set of 4800 haplotypes and a “European” panel containing 1000 haplotypes sampled from the 1600 CEU-like reference chromosomes. The first panel models the worldwide 1000 Genomes set, whereas the second panel models a single cluster of related populations (roughly speaking, the 1000 Genomes samples will be divided among five such clusters containing 500 individuals each). By imputing from both reference panels, we can see how reference panel size and diversity affect the computational loads of different imputation methods.

To provide a simple benchmark, we selected an imputation region that contained exactly 10,000 polymorphic sites in the European panel of 1000 haplotypes; this yielded the 1.9-Mb interval [11200000,13096366] on chromosome 17. This region contains a larger number of SNPs in the Cosmopolitan reference panel, but we restricted that panel to the same 10,000 SNPs to simplify the comparison. In practice, GWAS investigators may perform similar filtering on Cosmopolitan reference panels to remove variants that are underpowered in a particular study.

For each 10,000-SNP reference panel, we imputed the 1,000 GWAS individuals using IMPUTE v2.1.2 and Beagle v3.0.2. We used the default settings for both methods (for IMPUTE2, the default *k_hap_* setting is 500), and we also ran IMPUTE2 with *k_hap_* set to include all available reference haplotypes. We recorded the single-processor running times and memory requirements for each run.

## Results

We performed a series of experiments to evaluate our proposed imputation framework, which combines a cosmopolitan reference panel with a new approximation for speeding up imputation from large reference datasets.

We implemented our approximation within IMPUTE2, which uses an iterative algorithm to impute untyped variants in GWAS datasets. Whereas the original algorithm imputes genotypes from the full set of reference haplotypes, the new approximation imputes each study haplotype from a custom subset of reference haplotypes. (Study genotypes seldom come with known phase, but the haplotypes can be inferred as part of the algorithm.) Each of these custom reference panels includes the *k_hap_* reference haplotypes that have the fewest allele differences with a study haplotype at overlapping SNPs, where *k_hap_* is a user-defined parameter that controls the computational cost of imputation. If this method is applied over a limited genomic region (*e.g.* a few million base pairs rather than a whole chromosome), we expect the *k_hap_* reference haplotypes to be enriched for those that share recent common ancestry with the study haplotype of interest. We refer to these haplotypes as “surrogate family members” because, like real family members, they may share segments of nearly identical DNA that can be used for imputation. We explore the relationship between *k_hap_* and accuracy in the results that follow, and we provide practical suggestions for applying this approximation in the *Discussion*.

### HapMap 3 cross-validation experiments

We first tested our proposed imputation framework on a chromosome 20 dataset from HapMap 3. This dataset includes hundreds of haplotypes from each of several locations around the world, making it a good qualitative model for future reference panels like those being generated by the 1000 Genomes Project. The HapMap 3 panels used in this study are described in Table 1; note that we combined the CHB and JPT panels for this analysis.

Within each of the 10 panels, we masked a set of SNPs in one individual at a time and used IMPUTE2 to infer the hidden genotypes. We repeated this procedure across a range of *k_hap_* values and with various reference panels, which were creating by cumulatively adding HapMap 3 panels in the order dictated by genome-wide average *F_ST_*_._ One way to think of this procedure is to imagine building a composite reference panel for a population of interest: we start with a population-specific reference panel, and we successively add more-diverged panels to see if they will help (or possibly hurt) the imputation accuracy. These composite panels capture aspects of the Huang *et al.* (2009a) cross-validation strategy for choosing reference sets, and they provide population-label-informed benchmarks against which to compare our label-free way of using reference data.

#### Selected results for ASW and TSI:

**Figure 1:**
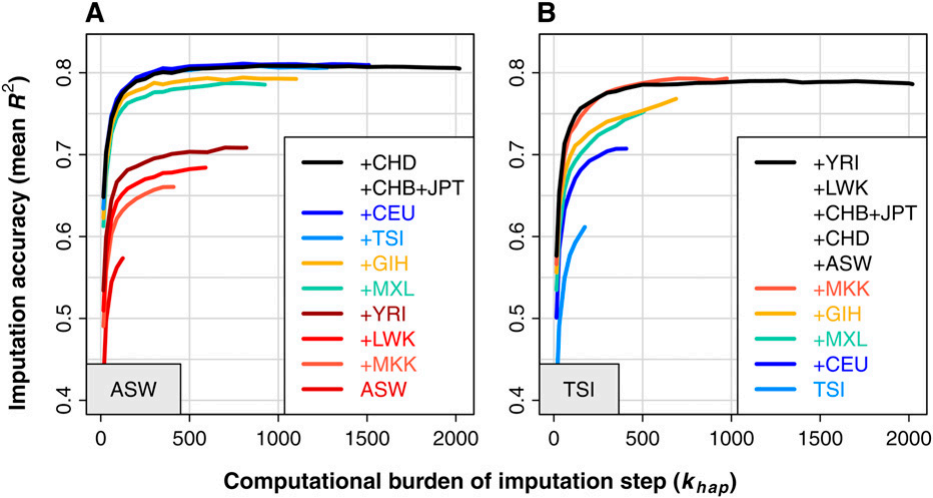
Imputation accuracy at low-frequency SNPs in HapMap 3 cross-validations in ASW and TSI, as a function of reference panel composition and *k_hap_* value. These plots show the imputation accuracy of IMPUTE2 in (A) the ASW panel and (B) the TSI panel. The accuracy of each experiment is plotted on the *y*-axis as the mean *R^2^* across all SNPs with MAF < 5% in the cross-validation panel (identified by the gray box in each plot). The *x*-axis shows the *k_hap_* parameter, which scales linearly with the computational burden of imputation updates in IMPUTE2. Each curve represents a different reference panel, with panels added cumulatively in the order shown in the legends, reading from bottom to top. Similar plots for other HapMap 3 target panels can be found in File S1.

In this section, we present results for the ASW (African American) and TSI (Italian) panels, which exhibit general trends and unexpected outcomes from our HapMap 3 cross-validations. Figure 1 shows how imputation accuracy depends on reference panel composition and the number of surrogate family haplotypes chosen from each composite panel (*k_hap_*). We constructed these plots in ways that highlight interesting features, and we restricted the results to SNPs with MAF < 5% in the target panel. We provide analogous plots for all HapMap 3 target panels in File S1.

The *x*-axis shows the value of the *k_hap_* parameter, and the *y*-axis shows the imputation accuracy, which is measured as the mean SNP-wise *R*^2^ between true and imputed allele dosages (posterior mean genotypes) for each cross-validation experiment. The computational cost of imputation is roughly proportional to *k_hap_*_._ Applying different *k_hap_* settings to a single reference panel generates a curve, and each curve represents a different reference panel. (For further details of the *R*^2^ distributions underlying the mean values at *k_hap_* = 500, see File S2.) Each individual was imputed initially from haplotypes in the same panel, then from reference sets that cumulatively added panels in the order shown in the plot legends, reading from bottom to top. The black curves represent our suggested strategy of using a cosmopolitan reference panel.

**Figure 2:**
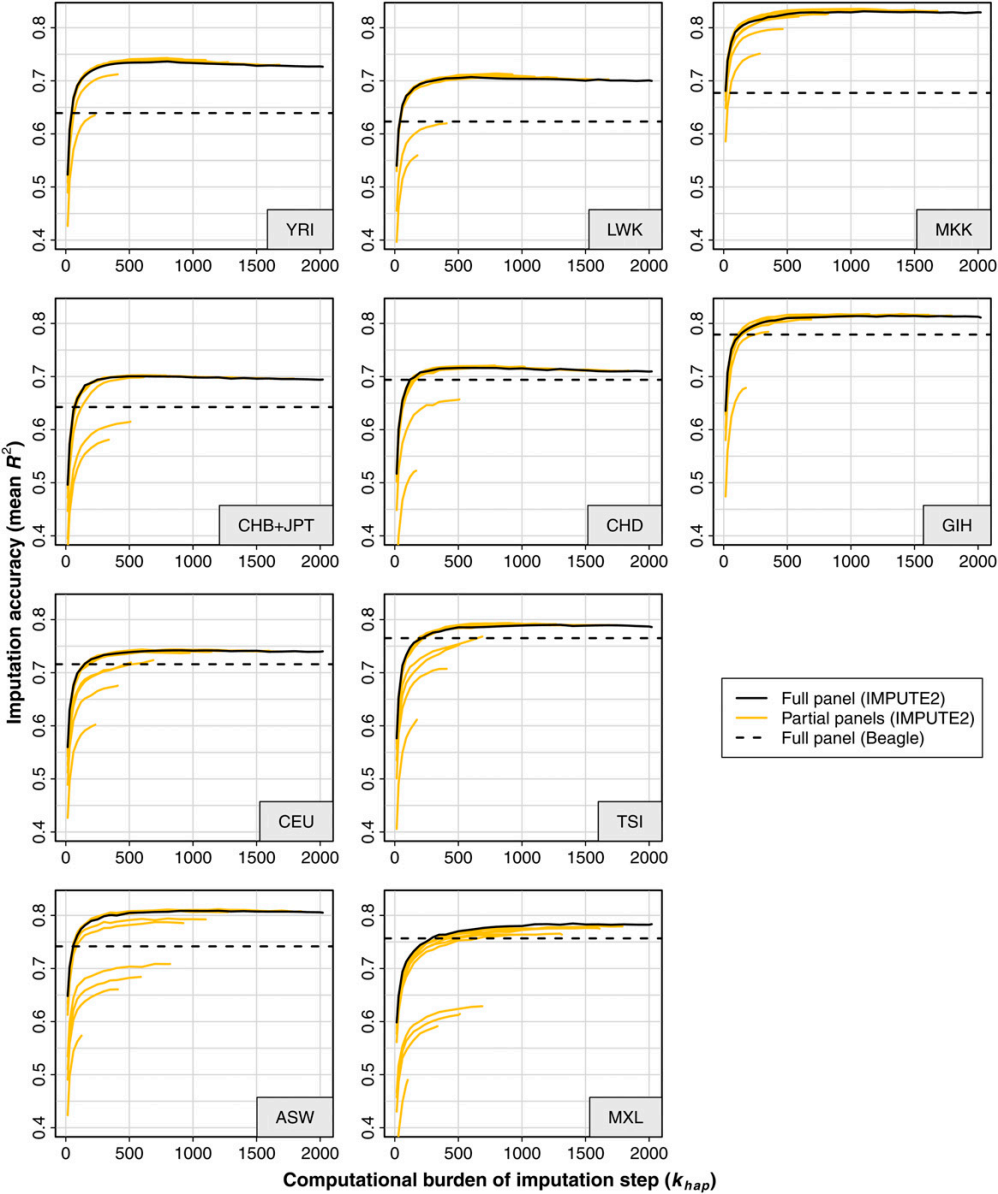
Imputation accuracy at low-frequency SNPs in HapMap 3 cross-validations, as a function of target panel, reference panel composition, *k_hap_* value, and imputation method. These plots show the imputation accuracy of IMPUTE2 and Beagle in various cross-validation experiments. The accuracy of each experiment is plotted on the *y*-axis as the mean *R^2^* across all SNPs with MAF < 5% in the cross-validation panel (identified by the gray box in each plot). The *x*-axis shows the *k_hap_* parameter, which scales linearly with the computational burden of imputation updates in IMPUTE2. The solid black curves show how *R^2^* varies with *k_hap_* when using IMPUTE2 with a reference panel containing the full set of 2020 HapMap 3 haplotypes; the dashed black lines show the accuracy of Beagle with this reference panel. IMPUTE2 was also applied to subpanels of the full HapMap 3 panel, with results shown as orange curves. Similar plots for other observed SNP sets and imputed SNP MAFs can be found in File S3.

One observation from Figure 1 is that the full set of reference haplotypes generated some of the highest accuracy levels in this experiment – the black curves almost always lie above the other curves. As we show in Figure 2 and File S3, this holds true across HapMap 3 target panels, genotyping arrays, and SNP frequency classes. Another salient feature of Figure 1 is that the curves plateau quickly with increasing values of *k_hap_* (moving from left to right within each plot). This shows that our surrogate family approximation can decrease computing time without losing accuracy. For example, the runs that selected 500 haplotypes from the full HapMap 3 panel (*k_hap_* = 500; black curves) achieved similar accuracy to the runs that didn't use this approximation (*k_hap_* = 2020), but the first set of runs was about four times faster.

Figure 1 also shows how imputation accuracy improved as particular panels were added to the reference set. The trends for the ASW panel (Figure 1A) are basically as expected: each successive African panel (MKK, LWK, and YRI) improved the accuracy incrementally, and the panels that capture components of European ancestry (MXL, GIH, TSI, and CEU) collectively raised the accuracy to its maximum value for this experiment. The east Asian panels (CHB+JPT and CHD) did not increase imputation accuracy any further, but nor did they reduce it; this demonstrates the ability of the method to ignore unhelpful reference haplotypes.

The results for the TSI panel (Figure 1B) are more surprising. As we would expect, accuracy improved with the addition of each panel that contains recent European ancestry (CEU and MEX). The GIH panel also increased the accuracy, which is reasonable given the relatively modest genetic divergence between this panel and TSI. Perhaps unexpectedly, the maximum imputation accuracy in TSI was not achieved until a set of African haplotypes (MKK) was added to the reference panel. This improvement was also observed when adding MKK haplotypes to the reference set for CEU imputation (File S1). Supplementary results from the HapMap 3 paper (The International HapMap Consortium 2010) show that the MKK panel has an admixture component that could reflect an ancient migration from Europe or the Middle East into eastern Africa, which might explain our results, although we observed a similar increase in accuracy after replacing the MKK panel with the remaining African panels (data not shown). Regardless of the underlying explanation, these results highlight the complexity of human demographic history, which is one motivation for frameworks like ours that use inclusive reference panels without population labels.

Notably, the common SNPs did not follow all of the patterns seen here for low-frequency SNPs (see File S3.) A cosmopolitan reference panel produced high imputation accuracy in both frequency classes, but the accuracy at common SNPs came almost entirely from the most closely related panels. For example, the maximum accuracy at common SNPs in TSI was attained with a TSI+CEU reference panel, and the addition of other panels neither increased nor decreased the accuracy. Hence, our results confirm that cosmopolitan reference panels are benign for imputing common variants, while showing that such panels can be positively helpful for imputing low-frequency variants. This difference can be understood in terms of the representation of the minor allele in the reference panel: common alleles are usually well-represented in population-matched panels of nontrivial size, whereas low-frequency alleles may be present in only a few copies or absent entirely, depending on allele frequency, SNP ascertainment scheme, and the number of reference haplotypes. Additional copies of these alleles (and their associated haplotype backgrounds) may sometimes be found in other populations, which explain why a cosmopolitan reference panel can improve imputation accuracy at low-frequency variants. These statements are supported by the results in File S2.

#### High-level results for all HapMap 3 panels:

We now extend the results from Figure 1 to the full set of HapMap 3 panels and a competing imputation method. We chose to compare against Beagle (Browning and Browning 2009) because past studies showed that it could achieve competitive speed and accuracy with large reference panels (Browning and Browning 2009; Howie *et al.* 2009; Jostins *et al.* 2011).

The high-level results of our cross-validation experiment are shown in Figure 2. As before, the solid black curves depict IMPUTE2 results with our suggested strategy of using a cosmopolitan reference panel, and different points on the *x*-axis correspond to different values of *k_hap_*_._ Here, the multicolored curves from Figure 1 (which represent imputation from subsets of the HapMap 3 haplotypes) are replaced with orange curves. The identities of the orange curves are omitted for plotting clarity, but full details are provided in File S1. We imputed and evaluated 1,523-2,364 low-frequency SNPs per HapMap 3 panel, with the exact numbers provided in Table S1. The black dashed lines in Figure 2 show the results of using Beagle with the full HapMap 3 reference panel; these lines are flat because Beagle does not have an analog of the *k_hap_* parameter.

As in Figure 1, our proposed framework always produced near-maximal accuracy. Also as before, the solid black curves typically reach their highest accuracy values at small values of *k_hap_*_._ Figure 2 shows that IMPUTE2 achieved higher accuracy than Beagle in every panel, except at the lowest *k_hap_* settings. In some target panels, the difference between methods was small; for example, IMPUTE2 was only slightly more accurate than Beagle in the CEU and TSI panels, which is consistent with previous results comparing these methods on a European dataset (Howie *et al.* 2009). [We note that Jostins *et al.* (2011) reached the contradictory conclusion that Beagle is more accurate than IMPUTE2 when imputing Europeans from diverse reference panels. We believe that their conclusion was driven by spurious IMPUTE2 results, as we explain in File S4.] On the other hand, IMPUTE2 was more accurate by a large margin in the African panels (YRI, LWK, and MKK). These trends cannot be attributed to the fact that we are running Beagle with a stratified reference panel when the method is not designed for that situation: IMPUTE2 also produced higher accuracy when we used reference panels that were well-matched to the target panels, both in the current HapMap 3 framework (data not shown) and in our MalariaGEN analyses (results below). We further note that the Beagle results shown here are better than the ones we obtained with smaller, less diverse HapMap 3 reference sets (data not shown). We tried running Beagle with larger values of its *niterations* and *nsamples* parameters, but there was essentially no change in these results (data not shown). We speculate on the mechanistic reasons for the accuracy differences between IMPUTE2 and Beagle in the *Discussion*.

A subtle feature of Figure 2 is that not all of the IMPUTE2 curves are monotonically increasing with *k_hap_*: some of the black curves peak at intermediate values of this parameter, then steadily decay as *k_hap_* grows to its maximum value of 2,020. This trend is clearest in the YRI panel, but it is also observable in other panels. There should be few problems of statistical computation (*e.g.* failure of the MCMC algorithm to converge) in our leave-one-out experiments, so we assume that this result reflects a real feature of the method. Our interpretation is that restricting the reference set via *k_hap_* actually imposes a more appropriate prior distribution on the haplotype copying probabilities when there is significant population structure in the panel. Tuning this prior by changing *k_hap_* has only a small effect on mean accuracy, which implies that our imputation method is largely robust to stratified reference data even without the surrogate family approximation. At the same time, this result implies that choosing custom reference panels may have benefits beyond just speeding up the computation, which is consistent with the conclusions of Pasaniuc *et al.* (2010).

It is not straightforward to compare these results to those of Huang *et al.* (2009a) because our experimental design is somewhat different. Nonetheless, we believe that our basic conclusions align with theirs. For example, we observed that the imputation accuracy with a worldwide reference panel (*k_hap_* = 2020) was never much lower than the accuracy with the optimal *k_hap_* (peaks of black curves in Figure 2), and the Huang *et al.* (2009a) results show a similar trend for cosmopolitan *vs.* optimal mixtures of HapMap 2 panels. In this sense, our *k_hap_* approximation can be viewed as a flexible and automatic way of implementing the Huang *et al.* (2009a) panel selection approach with an arbitrarily large number of reference populations.

### MalariaGEN cross-validation experiments

To assess whether our imputation strategy would yield similar benefits in African populations outside the HapMap 3 set, we performed additional cross-validations in a Gambian dataset from the Malaria Genomic Epidemiology Network (MalariaGEN; Malaria Genomic Epidemiology Network 2008). Previous work on imputing Gambians in a disease study found that the HapMap 2 YRI panel produced weaker association signals than did a Gambia-specific panel at the strongly selected beta-globin gene (Jallow *et al.* 2009). Here, we extend that work to examine imputation accuracy in larger reference panels and a broader variety of loci. We also rephrase the question to ask whether haplotypes sampled outside The Gambia can improve accuracy when a dedicated Gambian panel is available.

**Figure 3:**
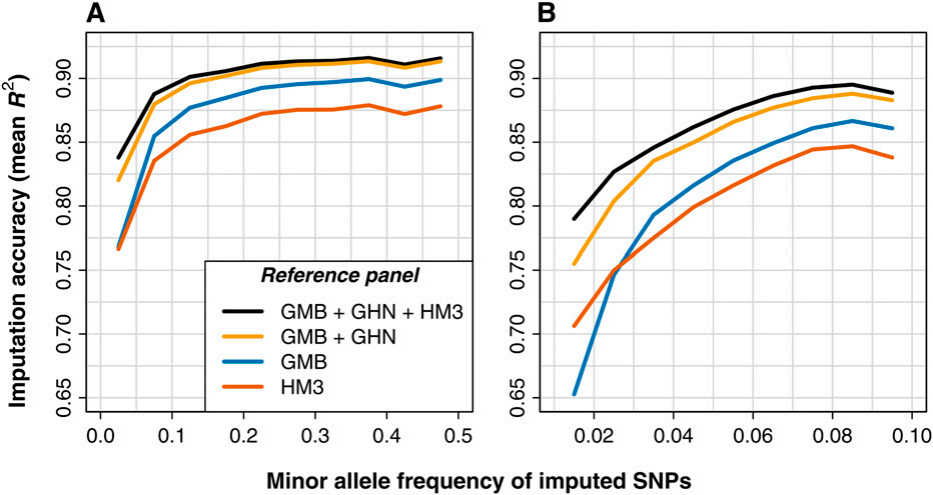
Imputation accuracy in Gambian validation set as a function of reference panel composition and minor allele frequency. These plots show the accuracy obtained when imputing masked SNPs in 1216 Gambian individuals from the MalariaGEN dataset using IMPUTE2 with *k_hap_* = 500. Each reference panel is represented by a different color, and the results are shown for (A) all SNPs and (B) SNPs with MAF < 10% in the Gambian validation set. The results are binned by MAF, with 5% bins in (A) and 1% bins in (B). Each point on a curve is located in the middle of the corresponding MAF bin. The following reference panel codes are used in the legend: GMB (Gambia, 200 haplotypes); GHN (Ghana, 200 haplotypes); and HM3 (HapMap 3, 2022 haplotypes).

To answer these questions, we masked and imputed 40,300 SNPs from the Illumina 650Y array in a set of 1216 Gambian individuals from MalariaGEN. We repeated the analysis for each of four reference panels: all 2022 HapMap 3 haplotypes (HM3); 200 Gambian haplotypes (GMB); 200 Gambian haplotypes plus 200 Ghanaian haplotypes (GMB+GHN); and all of the aforementioned panels combined (GMB+GHN+HM3; 2422 haplotypes). For each imputation run, we used IMPUTE2 with *k_hap_* = 500. In reference panels with fewer than 500 haplotypes, we reduced *k_hap_* to the number of available haplotypes. The results are shown in Figure 3, which breaks down the imputation accuracy by the minor allele frequencies of the SNPs in the 1216 imputed Gambians.

Figure 3A shows the results for all imputed SNPs, whereas Figure 3B is restricted to SNPs with MAF < 10%. We omitted SNPs with MAF < 1% from both plots as there were too few of these to provide reliable measurements. Like previous authors (Jallow *et al.* 2009), we found that a Gambia-specific reference set outperformed a HapMap set that did not include Gambians: the GMB panel (blue curves) produced higher overall accuracy than did the HM3 panel (red curves), despite the fact that there were ∼800 chromosomes of African ancestry in the HM3 set and only 200 chromosomes in the GMB set. This suggests that historical divergence between the populations in the HapMap 3 and Gambian panels makes HapMap 3 less accurate as an imputation resource.

The HapMap 3 panel is still useful, however. Across the allele frequency spectrum, the difference in mean *R*^2^ between the GMB and HM3 panels was never larger than 2.5%, and the difference was smallest at low-frequency SNPs. In fact, the HM3 panel was more accurate than the GMB panel for SNPs in the 1–2% and 2–3% MAF bands (Figure 3B); this shows that an ancestrally inclusive, nonspecific reference panel can capture low-frequency alleles that are poorly represented in a Gambia-specific panel. A recent simulation study found that association power and mean imputation *R*^2^ have a roughly linear relationship with a slope near 1.0 (Zheng *et al.* 2011), which suggests that using the HM3 panel in place of the GMB panel would cause only a small loss of power in an imputation-based association scan. [We note that the results from Zheng *et al.* (2011) appear to conflict with those of a recent study by Huang *et al.* (2009b), which found that power drops quickly with mean *R*^2^. We believe that the Zheng *et al.* (2011) results more accurately reflect this relationship because the mathematical model of imputation errors used by Huang *et al.* ignores the correlation of imputation error across individuals, which could be substantial, *e.g.* at hard-to-impute SNPs.

Figure 3 also shows that non-Gambian panels can improve imputation accuracy in the presence of a Gambian panel: the orange curve represents the addition of haplotypes from a moderately diverged population (GHN) and the black curve represents the further addition of a worldwide reference panel (HM3). These results support our strategy of using cosmopolitan reference panels: regardless of which reference data are available, the highest accuracy is achieved when using all available haplotypes and letting the imputation method decide which ones to use.

Aside from these questions of reference panel composition, we can also ask whether the choice of imputation method matters in African datasets. Our HapMap 3 comparisons suggest that Beagle has trouble imputing African genotypes, and we wanted to see if this conclusion would hold up in the MalariaGEN data. To address this, we used Beagle to impute the Gambian validation set using two different reference panels: (i) the GMB panel and (ii) a composite reference set containing the GMB panel, the GHN panel, and the HM3 panels with majority African ancestry (ASW, LWK, MKK, and YRI). We decided to reduce the HM3 set to these panels (which we label “HM3.afr”) in case Beagle’s difficulties in the previous comparisons were caused by the inclusion of non-African haplotypes in the reference panel. For consistency, we also imputed from the GMB+GHN+HM3.afr panel with IMPUTE2.

**Figure 4:**
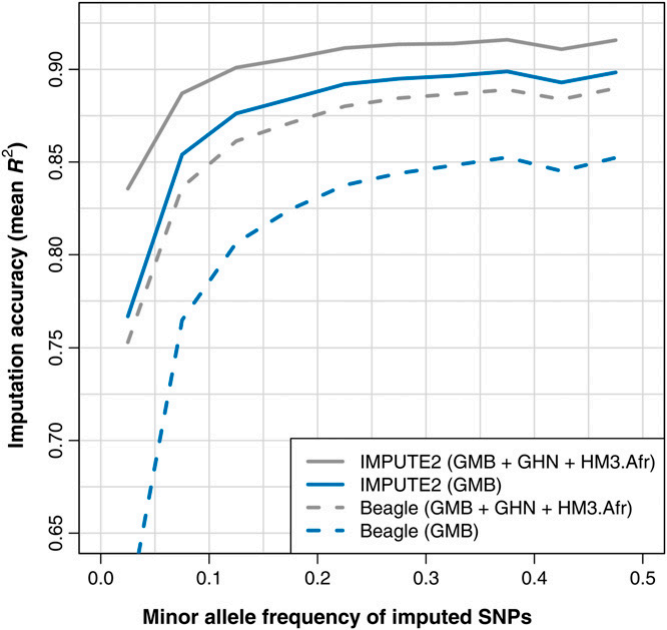
Comparison of imputation accuracy between IMPUTE2 and Beagle in Gambian validation set. This plot shows the accuracy obtained when imputing masked SNPs in 1216 Gambian individuals from the MalariaGEN dataset using either IMPUTE2 with *k_hap_* = 500 (solid lines) or Beagle on default settings (dashed lines). Imputation was performed with a reference panel of Gambian haplotypes (blue) and a reference panel of Gambian, Ghanaian, and HapMap 3 African ancestry haplotypes (gray). The results are grouped into 5% MAF bins, and each point on a curve is located in the middle of the corresponding MAF bin. The following reference panel codes are used in the legend: GMB (Gambia, 200 haplotypes); GHN (Ghana, 200 haplotypes); and HM3.afr (HapMap 3 African ancestry, 822 haplotypes).

The results of this comparison are shown in Figure 4. Results based on the GMB panel are shown in blue, while results based on the GMB+GHN+HM3.afr panel are shown in gray. Accuracy curves for IMPUTE2 and Beagle are drawn with solid and dashed lines, respectively. In concurrence with our previous results on African populations, we found that Beagle was much less accurate than IMPUTE2 when provided with the same reference panel: for each color, the solid line in Figure 4 is consistently above the dashed line. In fact, IMPUTE2 achieved higher accuracy when imputing from 200 GMB haplotypes (solid blue line) than Beagle did when imputing from 1222 African haplotypes (dashed gray line), of which the 200 GMB were a subset. The difference between methods was largest at low-frequency SNPs, but there was a substantial gap across the entire frequency spectrum.

We believe that Beagle's difficulties in imputing African datasets arise from properties of its clustering model. It is possible that Beagle could produce better results with different settings, although we tried varying both the model-fitting parameters (*niterations*, *nsamples*) and the model-building parameters (*scale*, *shift*) without observing a meaningful change in accuracy (data not shown).

### Computational benchmarking

To provide computational benchmarks for the imputation methods used in this study, we simulated two reference panels: one containing 4800 haplotypes modeled on the ancestrally diverse reference dataset that is being produced by the 1000 Genomes Project, and another containing 1000 haplotypes modeled on the European component of the 1000 Genomes set. We restricted the panels to a shared set of 10,000 SNPs spanning 1.9 Mb of sequence, and we imputed from both panels into a simulated GWAS of 1000 European individuals. The GWAS samples were provided with genotypes at SNPs that mirror the properties of the Affymetrix 500k platform; there were 337 such SNPs in the simulated region.

We imputed the simulated GWAS dataset from each reference panel using IMPUTE2 and Beagle. We ran Beagle on the same (default) settings as in the cross-validations, and we ran IMPUTE2 on two settings for each panel: *k_hap_* = 500 and *k_hap_* = *N*, where *N* is the number of haplotypes in the reference panel. As in the cross-validations, we fixed the IMPUTE2 phasing parameter at *k* = 80. Table 2 shows the single-processor running times and random-access memory (RAM) requirements of each program. We obtained these benchmarks from a single computer with 148 GB of RAM and a 2.4 GHz Intel Xeon processor.

**Table 2:** Computational benchmarks for a simulated GWAS of 1000 European individuals imputed from reference panels with 10,000 SNPs

Method	*k_hap_*	Reference Panel	Running Time (minutes)	RAM (GB)
IMPUTE2	500	European*^a^*	90	0.26
	500	Cosmopolitan*^b^*	127	0.60
	1000	European	157	0.30
	4800	Cosmopolitan	603	0.74
Beagle	—	European	655	5.2
	—	Cosmopolitan	5904	15.2

a The European panel contains 1000 haplotypes.

b The Cosmopolitan panel contains 4800 haplotypes with ancestry from Africa, Asia, and Europe.

Table 2 illustrates the computational benefits of our surrogate family approximation. In the Cosmopolitan reference panel with 4800 haplotypes, reducing *k_hap_* from 4800 to 500 decreased IMPUTE2’s running time by a factor of 4.8. Another way of viewing this is to notice that with *k_hap_* fixed at 500, IMPUTE2's running time increased by only a factor of 1.4 when moving from a panel with 1000 haplotypes to a panel with 4800 haplotypes. By comparison, Beagle's running time increased by a factor of 9 with the same panels. In this setting, fixing *k_hap_* fixes the cost of the imputation calculations used by IMPUTE2, so the 1.4-fold increase in running time at *k_hap_* = 500 reflects the additional time needed to evaluate a larger number of haplotypes when choosing which 500 to use for imputation. Preliminary experiments suggest that this evaluation step could be shortened by ignoring divergent haplotypes after the first few iterations of the algorithm (data not shown), which would make the overall running time almost independent of the number of reference haplotypes for fixed *k_hap_*_._

On these kinds of imputation datasets, IMPUTE2 shows clear computational advantages over Beagle. Even when using a large, ancestrally diverse reference panel, IMPUTE2 finished in less time on default settings (127 min with *k_hap_* = 500) than it took Beagle to impute from an ancestrally homogeneous panel with almost five times fewer haplotypes (655 min). IMPUTE2 also required much less RAM: for each reference panel, Beagle needed about 20 times more memory. These results, in combination with our cross-validation results, confirm that IMPUTE2 is both more accurate and more efficient than Beagle in the kinds of imputation datasets that are beginning to drive the field. As with any sophisticated inference method, there are ways to tweak Beagle's settings to achieve better speed, but all of them would reduce imputation accuracy. (Beagle's memory footprint can also be reduced, at the cost of even longer running times.) We explore some of the factors underlying these computational differences, and the implications they hold for future methods development, in the *Discussion*.

For fixed *k* and *k_hap_*_,_ IMPUTE2’s computational burden scales linearly with the number of study individuals, the number of reference haplotypes, the number of study SNPs, and the number of reference SNPs. Each of these factors makes a different per-unit contribution to the overall running time, with the number of study individuals and the number of reference SNPs having the biggest effect in modern datasets. Extrapolating the numbers from Table 2 to the entire genome and assuming the availability of 100 parallel computer processors, we predict that it would take IMPUTE2 about a day to impute 1000 individuals from a reference panel with thousands of sequenced haplotypes. For investigators with limited computational resources or very large GWAS cohorts, the imputation can be made even faster by prephasing the GWAS genotypes, as we explain in the *Discussion*.

## Discussion

Advances in DNA sequencing technologies have made it feasible to obtain near-complete genome sequences from thousands of individuals. Association mapping studies will immediately benefit from these developments: whole-genome sequencing of large GWAS datasets will not be practical for a while yet; in the meantime, we can impute a wide range of genetic variation from genomes that have already been sequenced. Most of the mutations discovered in these genomes will occur at low population frequencies, so it is important that imputation strategies be tailored to capture low-frequency variants. As sequencing projects produce larger and more diverse reference datasets, imputation-based GWAS will also face practical challenges like choosing appropriate reference panels and keeping computation tractable.

We have developed a coherent and convenient imputation framework that addresses these concerns. To simplify the process of choosing reference haplotypes from a diverse collection, our approach uses a cosmopolitan reference panel. Previous work suggested that cosmopolitan panels could increase imputation accuracy at low-frequency variants, and our results extend these findings to a wide range of human populations. It is computationally intensive to perform imputation with large, ancestrally diverse reference panels, so we have also developed an approximation that decreases the cost of adding haplotypes to a reference set without sacrificing accuracy.

Our framework was motivated by the idea that larger reference datasets should make imputation faster and simpler, rather than slower and more complicated. We believe that our work represents a first step toward bridging current imputation practice with the paradigm suggested by Kong *et al.* (2008), in which large population samples eliminate the need for complex models and reference panel selection, and investigators do not have to balance efficiency and accuracy. Detailed population models and reference panel weighting schemes may provide modest accuracy improvements in the short term, but we expect that the power gains from such developments will seldom justify the added computational costs.

Conversely, imputation strategies that are agnostic to population labels (as ours is) may become increasingly attractive as sequencing studies fill in the continuum of human genetic diversity. One benefit of such approaches is that they can capture unexpected allele sharing without needing to model the complexities of human demographic history, as we demonstrated by showing that African haplotypes can improve imputation accuracy in Europeans. Approaches like ours are also well-suited for imputation in recently admixed populations: methods that choose custom reference panels for different admixed individuals in different parts of the genome can increase accuracy by adapting to local ancestry changes, as previously suggested by Pasaniuc *et al.* (2010).

The framework we have proposed can serve as a general approach for using reference data in current and future imputation studies, and we expect that it will spur additional methods development in this area. Below, we discuss the aspects of this framework that make it successful in modern reference panels, along with some practical and theoretical questions that may arise when extending the conclusions of this study to other datasets.

### Extending our results to future studies

Our cross-validation experiments have provided a wealth of information about how to use existing imputation resources like HapMap 3, but these datasets do not capture the full range of features that will be present in future reference panels. For example, our results are based on data from commercial SNP arrays, whose composition is biased toward variants that share alleles across populations. Consequently, population-specific accuracy contributions like the ones seen in Figure 1 should not be treated as quantitative predictions for newly discovered variants. While we could have used 1000 Genomes data to address the SNP ascertainment issue, the data available when we were preparing this manuscript contained smaller sample sizes and a narrower sampling of human genetic diversity than found in HapMap 3, so we decided to focus on the latter dataset as a model of future 1000 Genomes reference panels. We have run similar imputation experiments with an interim release of the 1000 Genomes Phase I haplotypes, and we have continued to see benefits from using ancestrally inclusive reference panels (B. Howie; unpublished data).

In this work, we have highlighted the fact that combining reference data from different populations can improve imputation accuracy at low-frequency variants. This finding reflects both the limited sample sizes of existing reference panels and the shared ancestry of human populations: an allele that occurs at low frequency in a study population may be poorly represented in a well-matched reference panel due to sampling effects; however, that same allele may be found in reference sets from other populations due to genetic drift or introduction by recent migrants. While a multipopulation reference panel can improve accuracy at this kind of variant, there are other situations in which accuracy might be harmed by such panels. Possible mechanisms for decreased accuracy include (i) the imputation of variant alleles at sites that do not segregate in a study dataset, (ii) signal dilution from reference haplotypes that are similar to those in a study population but do not carry a variant allele that segregates in that population, and (iii) misleading results from reference haplotypes that carry recurrent mutations. We discuss these issues in File S5; we conclude that they will seldom hurt the imputation of low-frequency alleles from HapMap 3 or 1000 Genomes haplotypes, but that reference panel composition may need to be reevaluated when imputing rare alleles (MAF < 0.5%) or using other reference datasets.

Another question to consider when applying our framework is whether the optimal number of surrogate family haplotypes will change with different reference datasets. Judging from our experience in a variety of studies, we suggest the rule of thumb that *k_hap_* should be set to the number of reference haplotypes that have broadly similar ancestry to the study population. For example, the broad ancestral groupings in HapMap 3 (Europe, East Asia, Africa) each include 500–800 haplotypes, and we found that *k_hap_* = 500 worked well with this resource. Imputation accuracy is not highly sensitive to this variable, regardless of other factors like chunk size and local recombination rate, so it should not usually be necessary to optimize *k_hap_* empirically. As reference sets grow and we further develop our approximation, we anticipate that it will be possible to achieve high accuracy with even lower values of *k_hap_*_._

### Suggestions for imputation-based GWAS in Africa

African populations pose a special challenge for imputation because they are among the most genetically diverse in the world (Rosenberg *et al.* 2010). Genetic relationships among African populations have been shaped by complex demographic histories, deep ancestries, and strong selective pressures, which can cause patterns of haplotype sharing in Africa to look much different than patterns in other parts of the world (Bryc *et al.* 2010; Campbell and Tishkoff 2008, 2010; Reed and Tishkoff 2006; Tishkoff *et al.* 2007, 2009). African populations also carry substantial burdens of common disease, yet few large-scale GWAS have been conducted in this setting. Efforts like the 1000 Genomes Project and MalariaGEN are changing this landscape, so it will be important to define effective reference panels for GWAS in a variety of African populations.

We addressed this question by performing cross-validation experiments in Gambian individuals from MalariaGEN. In concurrence with a more limited analysis by Jallow *et al.* (2009), we found that a population-specific reference panel yielded higher average accuracy than did a larger HapMap 3 panel that lacked Gambian haplotypes. However, we also found that the HapMap 3 panel produced reasonable imputation accuracy across the allele frequency spectrum, and that non-Gambian haplotypes improved accuracy when added to a Gambia-specific reference panel.

Although these findings were obtained from a limited sampling of African genetic diversity, they provide some intuition about general strategies for imputation in people of recent African ancestry. It will always help to collect new reference data through population-specific sequencing or genotyping, but the gains from this approach will often be larger in Africa. Whether or not a well-matched panel is available for a particular study population, imputing from all available haplotypes of African ancestry may often improve the results. Non-specific reference panels can weaken association signals near loci that have recently experienced strong selection, as with the beta-globin region in the Jallow *et al.* study, so it may be worthwhile to re-impute from just the ancestry-matched reference haplotypes (when such haplotypes are available) in regions showing decisive evidence of selective sweeps.

Imputation methods based on the Li and Stephens (2003) model of DNA sequence variation (like IMPUTE2 and MaCH) are well suited to performing imputation in African GWAS. As shown in our HapMap 3 and MalariaGEN cross-validations, this kind of model can consistently produce higher accuracy than clustering approaches like the one used by Beagle, for reasons we discuss below. When the model is implemented via an efficient algorithm like IMPUTE2, this accuracy can be achieved at a fraction of the computational price.

Looking ahead, we anticipate that many of the initial African GWAS will be conducted in west African populations; for example, such populations constitute a large part of MalariaGEN's Consortial Project 1. The power of these studies could potentially be boosted by using African American haplotypes to augment the reference sets collected in Africa. While there are clear merits to this idea, one might worry that the haplotype segments of non-African ancestry would pose problems for imputation. We can address this question by inspecting the IMPUTE2 results from our HapMap 3 cross-validations. Encouragingly, these results show that adding the HapMap 3 ASW panel to the reference set improved accuracy in every African cross-validation panel (File S1).

### Computational strategies for imputation with large, sequence-based reference panels

Throughout our imputation experiments, we found that IMPUTE2 can attain both higher accuracy and faster computation than Beagle, which is a leading inference method for large datasets. We believe that the success of IMPUTE2 in this context can be attributed to its computational strategies and its model of DNA sequence variation. We discuss these attributes here in hopes that they will inform future methods development, and we address the role that prephasing can play in speeding up imputation.

Beagle's basic modeling approach is to combine haplotypes into clusters. This speeds up computation because it restricts the number of HMM states that need to be considered: rather than perform HMM calculations on every haplotype in a dataset, Beagle can run the calculations on a smaller set of clusters. Similar state-reduction techniques are used by GERBIL (Kimmel and Shamir 2005), fastPHASE (Scheet and Stephens 2006), GEDI (Kennedy *et al.* 2008), and other related methods. By contrast, the basic HMM used by IMPUTE2 and MaCH includes a state for every haplotype. Using all of the states makes computation intractable, which is why IMPUTE2 restricts the states via its *k* and *k_hap_* parameters. The intuition is that the “surrogate family members” identified in this way should include the most informative haplotypes for a particular individual in a particular part of the genome.

Both of these state-reduction approaches speed up imputation, but our cross-validations show that IMPUTE2 attains higher accuracy than Beagle in practice, especially at low-frequency variants in datasets that have higher haplotype diversity (*e.g.* those with recent African ancestry). We suggest that this is because clustering models have inherent difficulties capturing low-frequency variation: by grouping similar haplotypes into clusters, these methods obscure the differences between those haplotypes, which reduces the ability to impute low-frequency variants. This could explain why the accuracy disparity between IMPUTE2 and Beagle was largest in African populations, which have higher genetic diversity than non-African populations and hence a larger fraction of low-frequency haplotypes. Methods like Beagle may be able to make up some of this ground by using more clusters, but this will further increase the computational load.

These trends should persist as imputation datasets continue to grow: clustering models will need to add even more states to their HMMs to remain competitive on accuracy, whereas the closest *k* (or *k_hap_*) surrogate family haplotypes will become even more informative, thereby enhancing the running time and accuracy advantages of methods like IMPUTE2. The natural endpoint of this process will arrive when so many genomes have been sequenced that imputation requires just a handful of the closest genealogical neighbors, which is where “surrogate parent” methods, like the one developed by Kong *et al.* (2008), will take hold. Until that point is reached, we suggest that our surrogate family approximation will remain an attractive way to balance accuracy and speed.

Another technique for increasing the efficiency of imputation is called “pre-phasing.” The idea is to (pre-)phase the assayed genotypes in a GWAS dataset, then impute directly into the inferred haplotypes; this speeds up imputation by more than an order of magnitude at the cost of a small amount of accuracy (B. Howie and C. Fuchsberger, unpublished data). In principle, most imputation methods could use this approach, and researchers can already download implementations based on the IMPUTE2 and MaCH models (the MaCH implementation is called “minimac”). We have found that *k_hap_* has similar accuracy characteristics in both unphased and pre-phased GWAS datasets (data not shown), so we view pre-phasing as being complementary to our surrogate family approximation: both approaches speed up imputation, and they can be used together for even greater efficiency.

### Extensions

One potential extension of the results and methodology seen in this study is to whole-genome sequencing efforts like the 1000 Genomes Project. One study design that has arisen in this context is to sequence many individuals at low coverage; say, 2–4×. The data from this kind of experiment are too sparse to directly and confidently determine most genotypes, but they can be called with high accuracy by applying the same kinds of models that are used for genotype imputation in GWAS (Li *et al.* 2011; The 1000 Genomes Project Consortium 2010). We expect that the approach of combining information across populations will help call low-frequency alleles in that setting, much as it helped impute low-frequency alleles in this study.

## Supplementary Material

Supporting Information
