# Supplementary material for: Genotype Imputation with Thousands of Genomes
Source: G3 (Bethesda). 2011 Nov 1;1(6):457–70. doi: 10.1534/g3.111.001198 (PMC3276165; doi:10.1534/g3.111.001198)
Supplement: Supporting Information [file supp_1_6_457__index.html]

Supporting Information 

# Genotype Imputation with Thousands of Genomes

## Supporting Infomation for Howie, Marchini, and Stephens, 2011

**Files in this Data Supplement:**

- Supporting Information - Files S1-S6 and Tables S1 and S2 (PDF, 6 MB)
- File S1 - Figures S1-S10 (PDF, 1.8 MB)
- File S2 - Figures S11-S15 (PDF, 2.6 MB)
- File S3 - Figures S16-S18 (PDF, 1.2 MB)
- File S4 - Discrepancy with Impute2 vs. Beagle Comparison in Jostins *et al.* (PDF, 72 KB)
- File S5 - Potential Problems in Multi-population Reference Sets (PDF, 64 KB)
- File S6 - Relationship between khap and a Coalescent-based approximation (PDF, 116 KB)
- Table S1 - Number of low-frequency SNPs imputed in each HapMap 3 panel from Affymetrix 6.0 SNPs (PDF, 40 KB)
- Table S2 - Discovery probabilities for SNPs by variant allele count in simulated reference panels (PDF, 36 KB)
