# Supplementary material for: Genotype Imputation with Thousands of Genomes
Source: G3 (Bethesda). 2011 Nov 1;1(6):457–70. doi: 10.1534/g3.111.001198 (PMC3276165; doi:10.1534/g3.111.001198)
Supplement: Supporting Information [file supp_1.6.457_001198SI.pdf]

File S1

Figures S1-S10

These figures show detailed results from cross-validation experiments in HapMap 3. A set of SNPs was masked in one individual at a time, then imputed with various reference panels and k_{hap} settings. Each masked individual was provided with observed genotypes at Affymetrix 6.0 SNPs. The accuracy of each experiment is plotted on the *y*-axis as the mean R^2 across all imputed SNPs with MAF < 5% in the cross-validation panel (identified by the grey box in each figure). The *x*-axis shows the k_{hap} parameter, which scales linearly with the computational burden of imputation updates in IMPUTE2. Each figure contains results for a single HapMap 3 cross-validation panel, in two parts:

(A) These plots are similar to the ones shown in Figure 1 of the main text. Each curve represents a different reference panel, with panels added cumulatively in the order shown in the legend, reading from bottom to top. All results are from IMPUTE2.

(B) These plots are identical to the ones shown in Figure 2 of the main text. Results from the full HapMap 3 reference panel of 2,020 haplotypes are represented in black (solid line for IMPUTE2, dashed line for Beagle), and results from sub-panels of the HapMap 3 set are shown in orange (IMPUTE2 only). This way of displaying the results highlights our proposed strategy, which is to use all available reference haplotypes for imputation in every population.

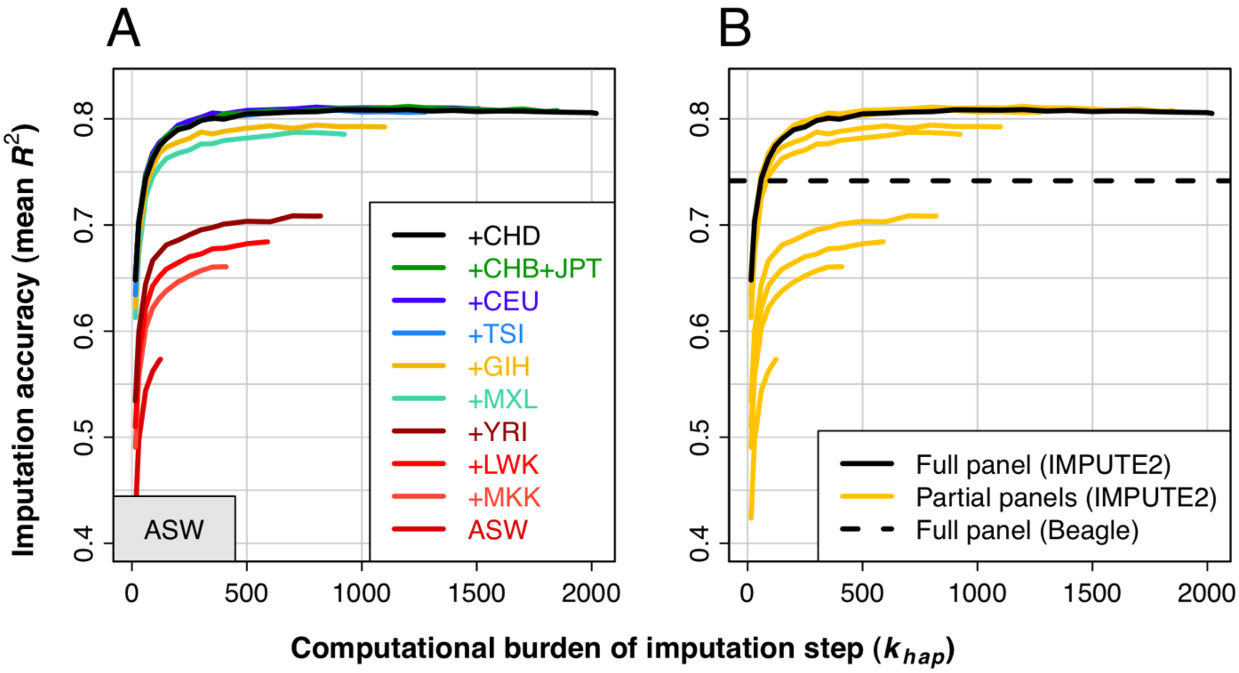

Figure S1 Imputation accuracy at low-frequency SNPs in HapMap 3 cross-validations in ASW, as a function of reference panel composition, k_{hap} value, and imputation method. Further details can be found at the start of this section.

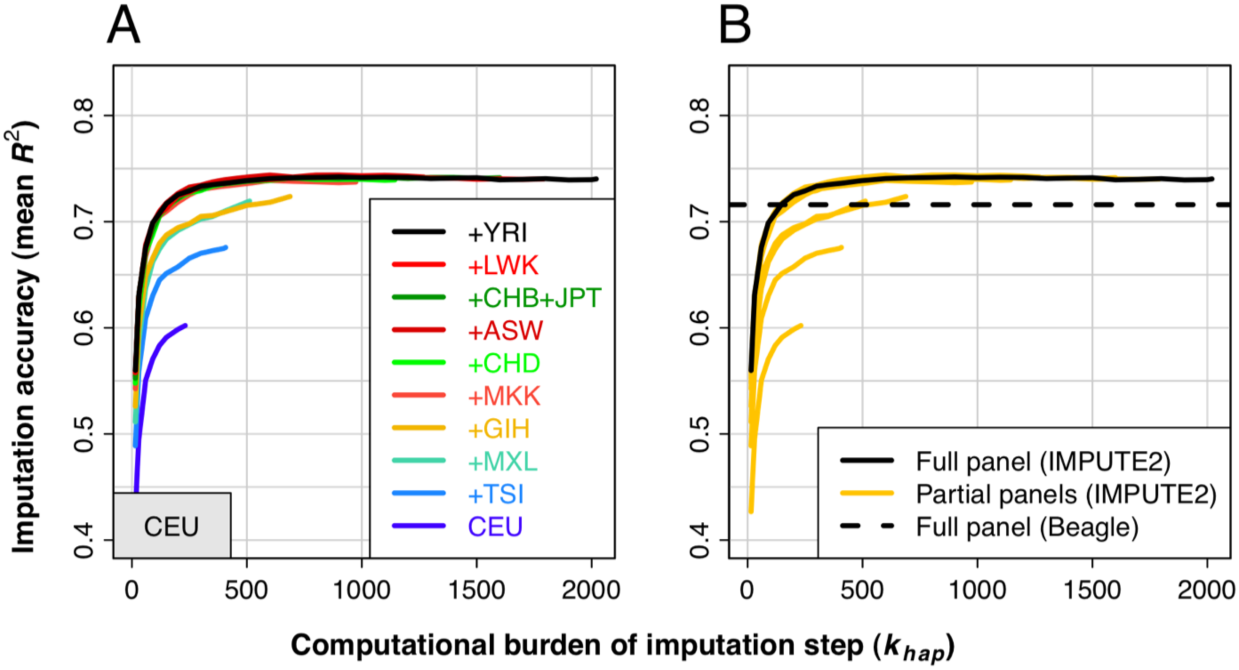

Figure S2 Imputation accuracy at low-frequency SNPs in HapMap 3 cross-validations in CEU, as a function of reference panel composition, k_{hap} value, and imputation method. Further details can be found at the start of this section.

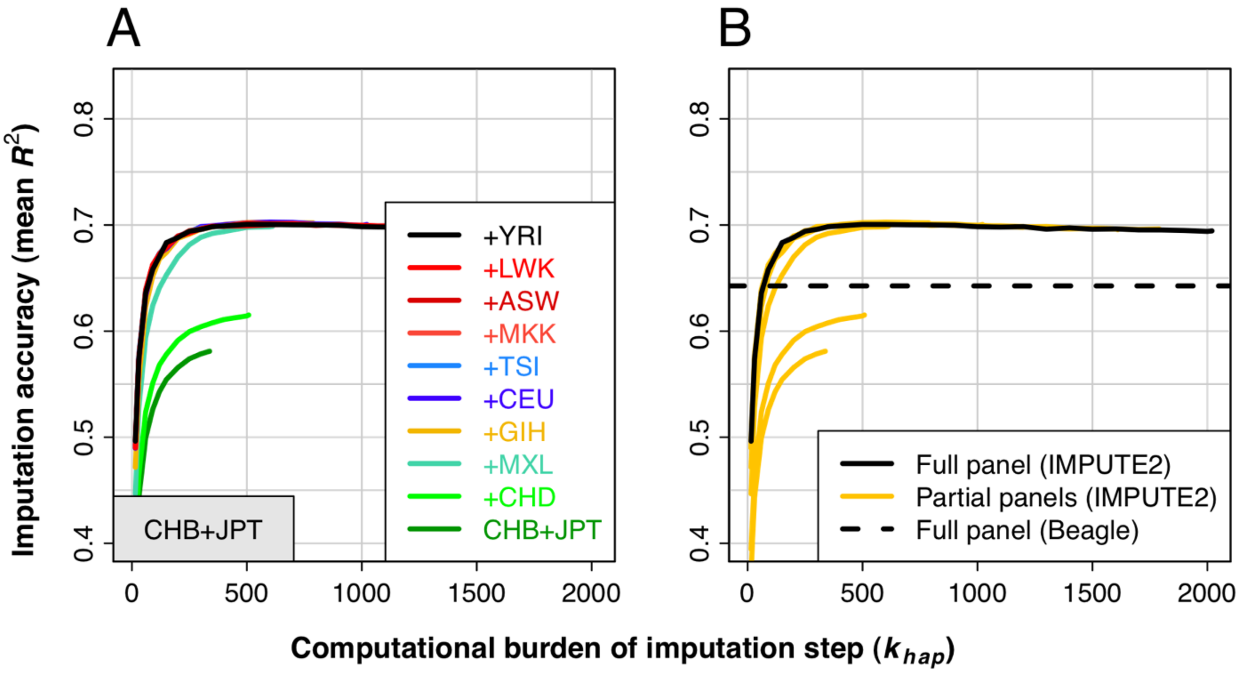

Figure S3 Imputation accuracy at low-frequency SNPs in HapMap 3 cross-validations in CHB+JPT, as a function of reference panel composition, k_{hap} value, and imputation method. Further details can be found at the start of this section.

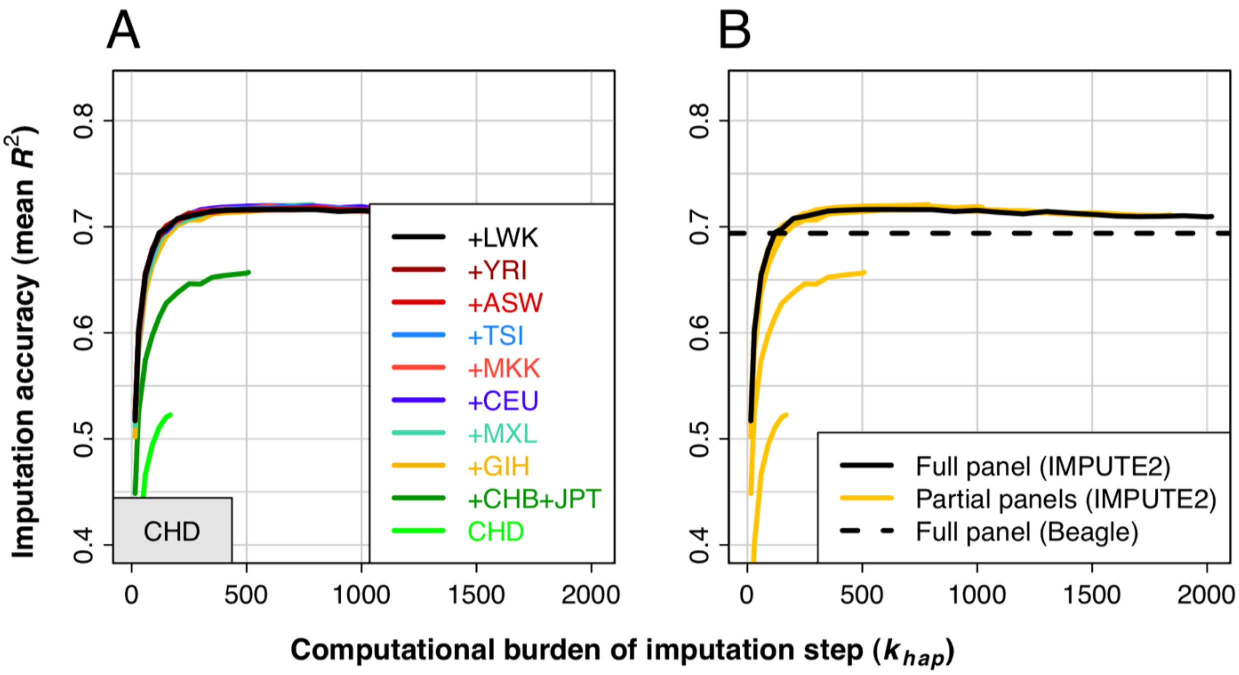

Figure S4 Imputation accuracy at low-frequency SNPs in HapMap 3 cross-validations in CHD, as a function of reference panel composition, k_{hap} value, and imputation method. Further details can be found at the start of this section.

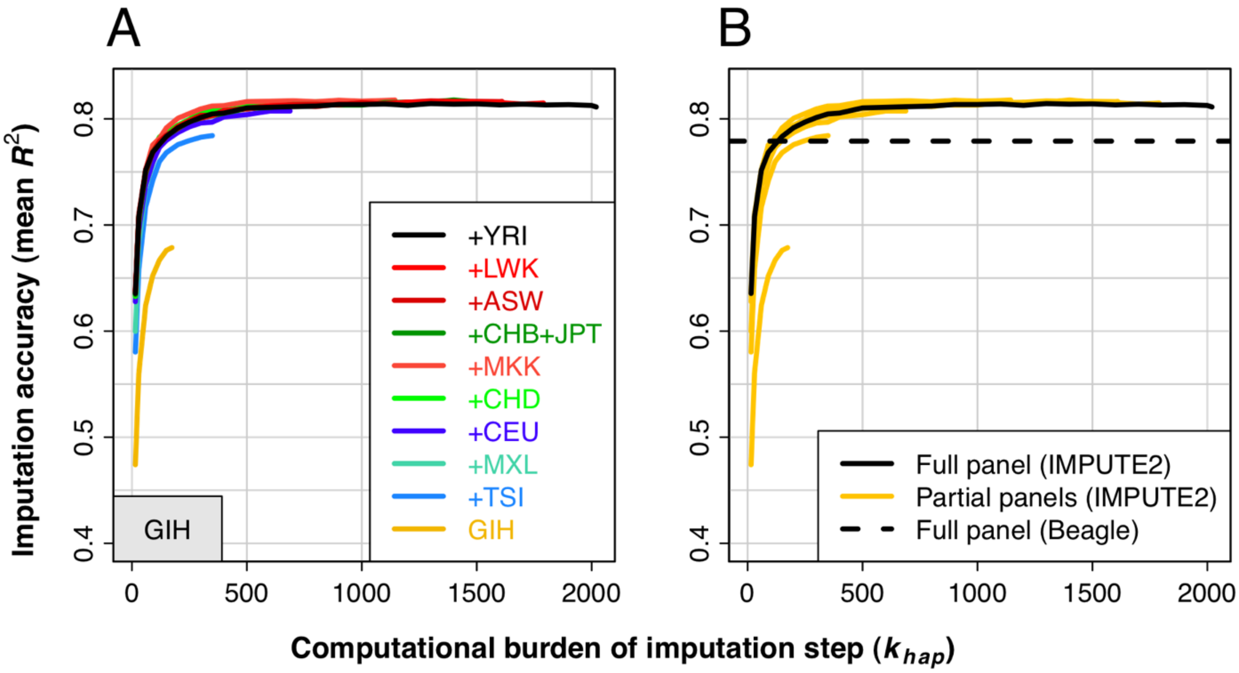

Figure S5 Imputation accuracy at low-frequency SNPs in HapMap 3 cross-validations in GIH, as a function of reference panel composition, k_{hap} value, and imputation method. Further details can be found at the start of this section.

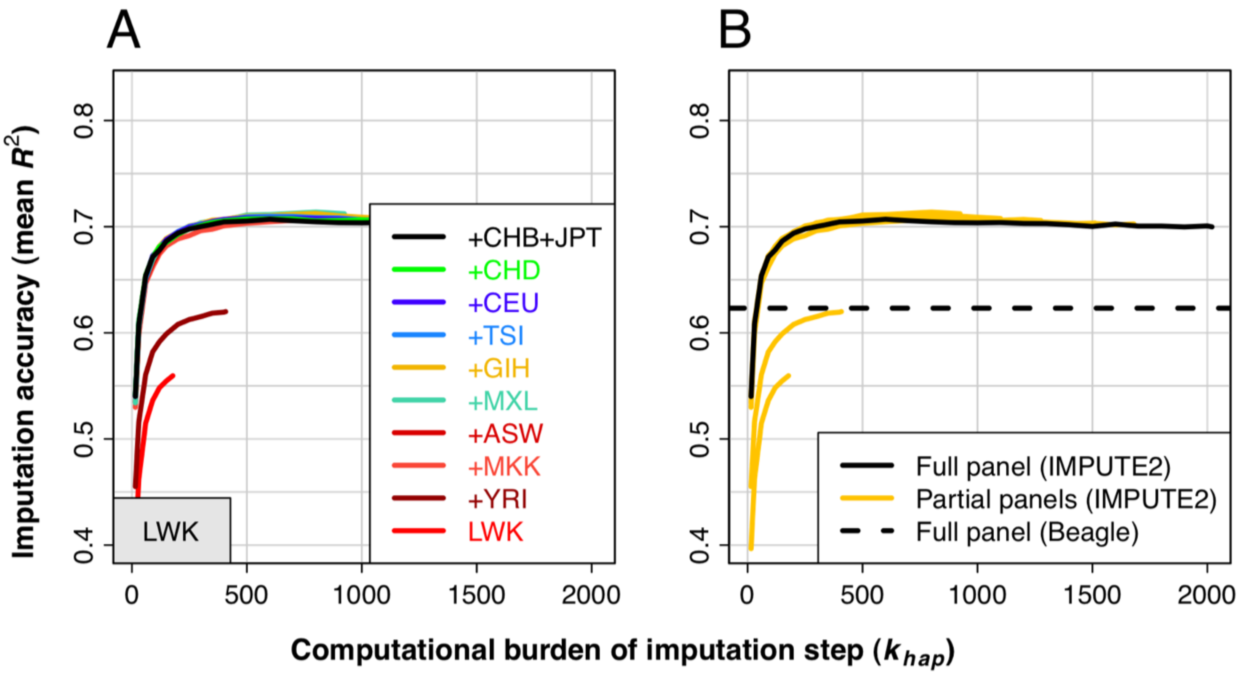

Figure S6 Imputation accuracy at low-frequency SNPs in HapMap 3 cross-validations in LWK, as a function of reference panel composition, k_{hap} value, and imputation method. Further details can be found at the start of this section.

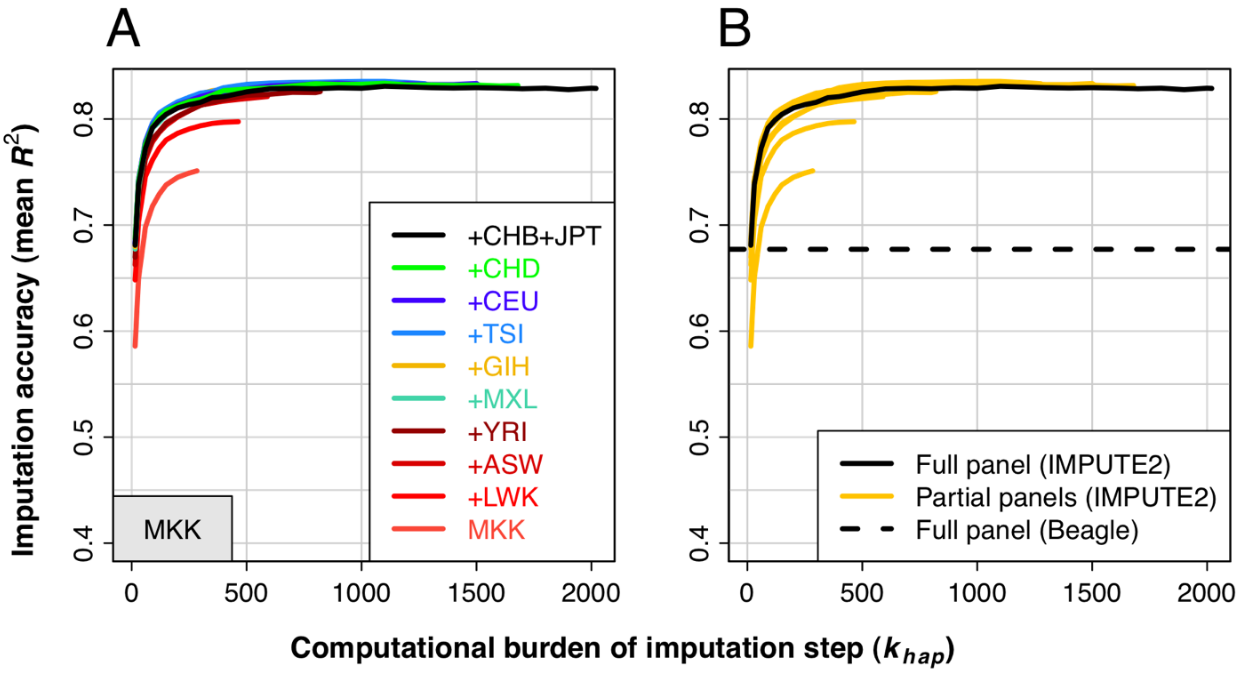

Figure S7 Imputation accuracy at low-frequency SNPs in HapMap 3 cross-validations in MKK, as a function of reference panel composition, k_{hap} value, and imputation method. Further details can be found at the start of this section.

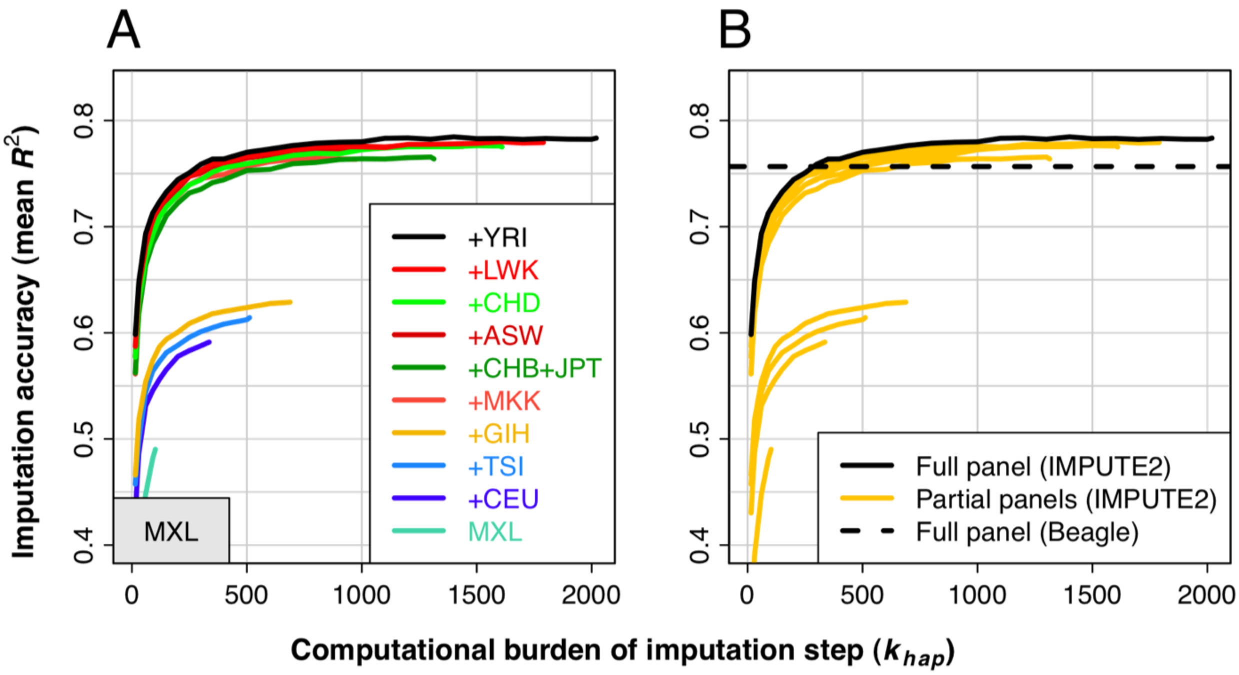

Figure S8 Imputation accuracy at low-frequency SNPs in HapMap 3 cross-validations in MXL, as a function of reference panel composition, k_{hap} value, and imputation method. Further details can be found at the start of this section.

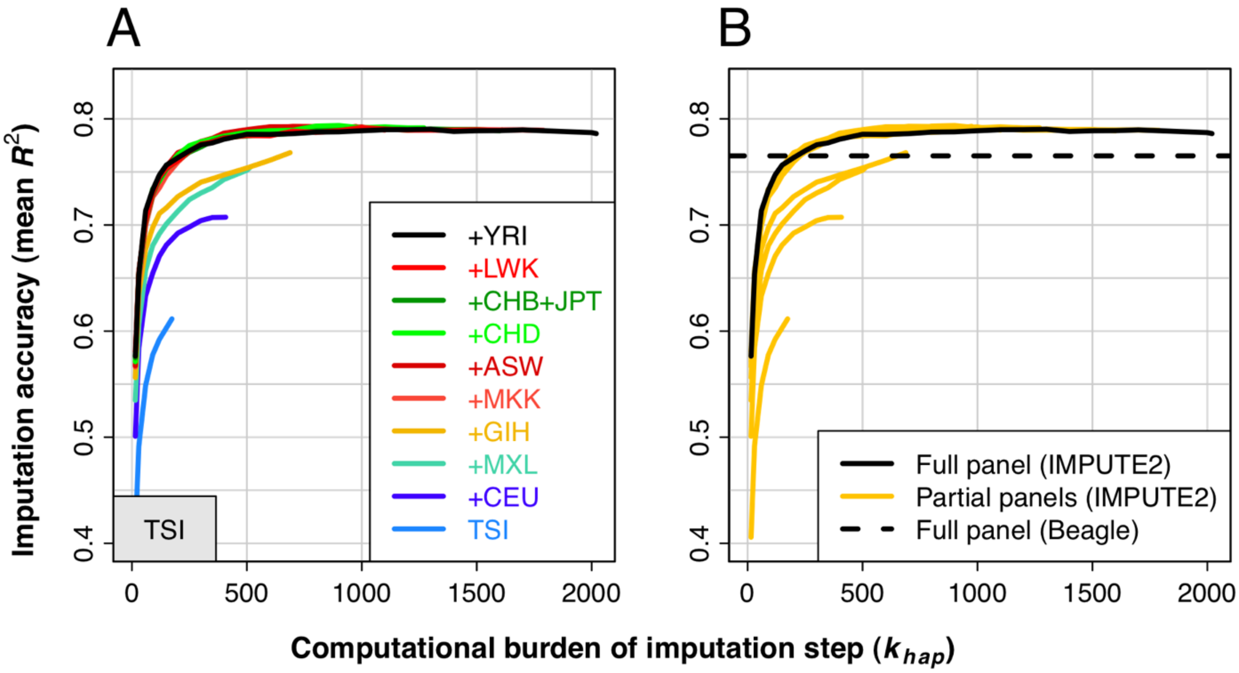

Figure S9 Imputation accuracy at low-frequency SNPs in HapMap 3 cross-validations in TSI, as a function of reference panel composition, k_{hap} value, and imputation method. Further details can be found at the start of this section.

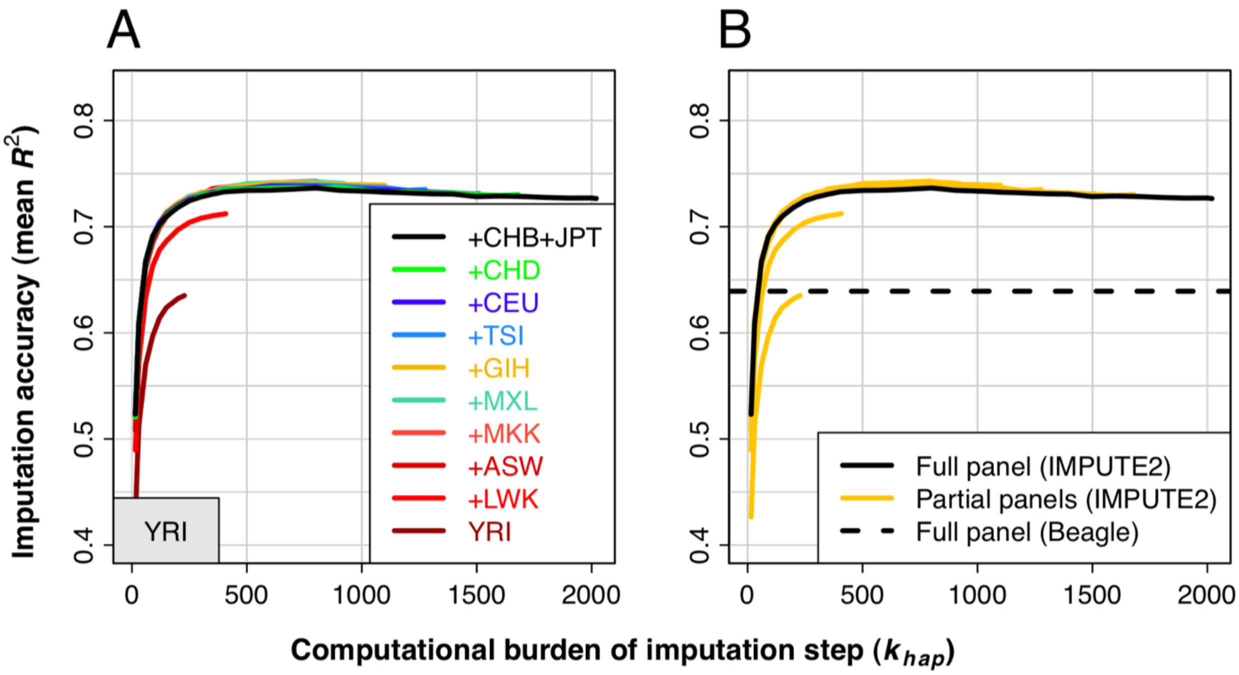

Figure S10 Imputation accuracy at low-frequency SNPs in HapMap 3 cross-validations in YRI, as a function of reference panel composition, k_{hap} value, and imputation method. Further details can be found at the start of this section.

File S2

Figures S11-S15

These figures provide a deeper explanation of the results presented in Figures S1-S10. For each HapMap 3 panel, we took slices of the IMPUTE2 accuracy curves in those figures at k_{hap} = 500, and we expanded the mean R^2 values to full cumulative distributions of SNP-wise R^2 values.

Each column corresponds to a single cross-validation target panel from HapMap 3, as labeled by the grey box in the topmost plot. Reading from top to bottom, the plots display R^2 distributions for SNPs with 1, 2, 3, and *N* copies of the minor allele in the target panel, where *N* is the number that corresponds to MAF = 20% in that panel. The different curves within a plot show the R^2 distributions for reference panels with various levels of inclusiveness, with red representing imputation within the target panel and black representing imputation from the full HapMap 3 panel. We allocated the intermediate colors (blue and orange) separately for each target panel to capture interesting features; the composite reference panels they represent can be determined by reading the plot legends from bottom to top.

The topmost plots in each figure show the results for SNPs with a single minor allele copy (singletons). These SNPs are a special case: in a leave-one-out analysis within the target panel, it should be impossible to correctly impute a singleton allele since each such allele is removed from the reference panel when the individual carrying it is masked. This scenario models alleles that exist in a population at low frequency but were not sampled in a population-specific reference panel. The red curves in the topmost plots are concentrated almost entirely at R^2 = 0 since a variant allele cannot be imputed when it is not represented in the reference panel; non-zero values in the other curves correspond to alleles that were “rescued” through the inclusion of reference panels from other populations. (Note that the red curves occasionally achieve non-zero R^2 values, as in the ASW panel, through stochastic fluctuations in the imputation.)

These results confirm that ancestrally diverse reference panels are most beneficial at SNPs with low MAFs, whereas common SNPs are imputed well regardless of panel composition. Our pro- posed strategy of using all available reference haplotypes leads to the highest accuracy levels in most of these plots.

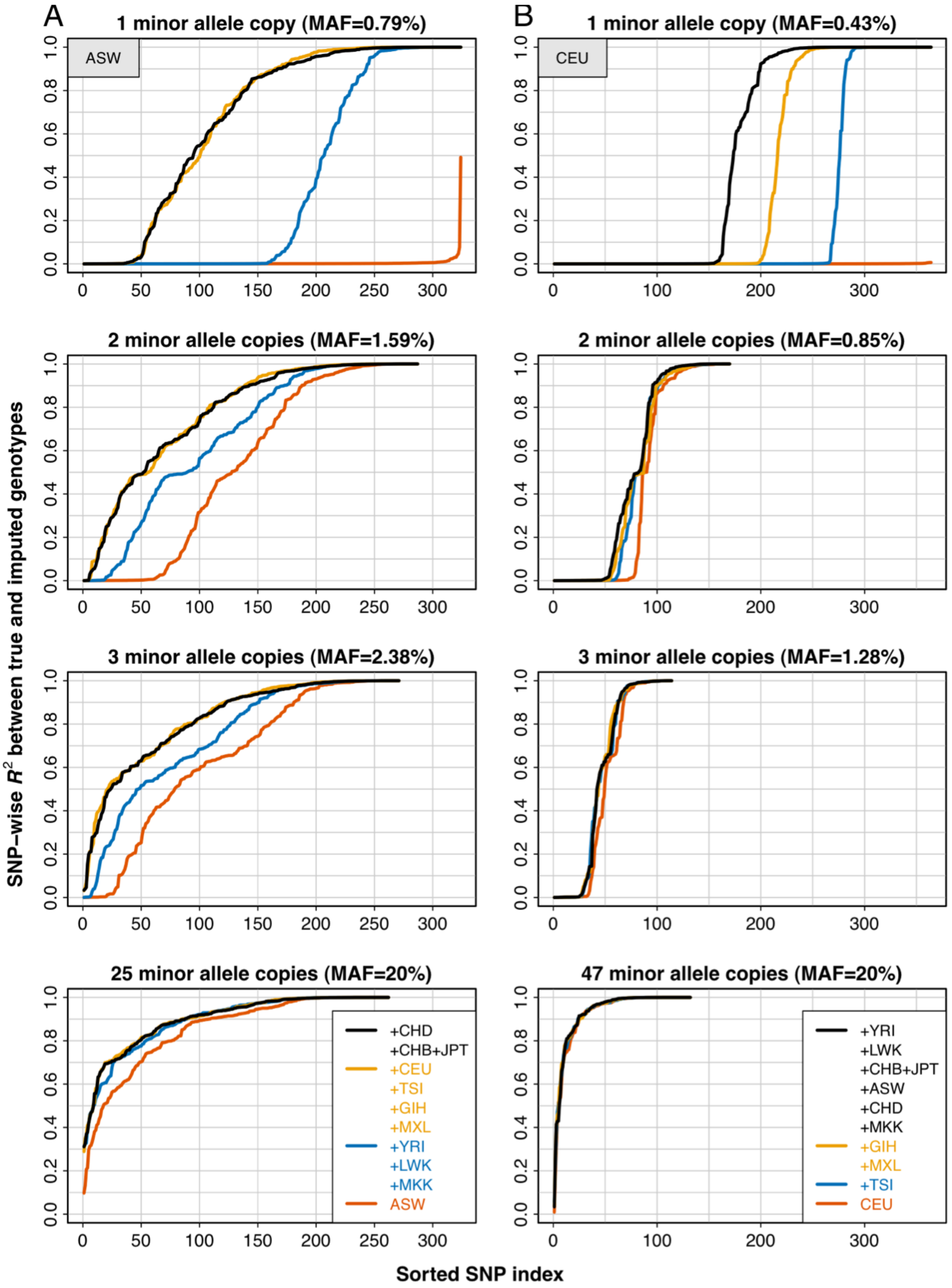

Figure S11 Cumulative distributions of SNP-wise imputation accuracy (R^2) in (A) ASW and (B) CEU, as a function of reference panel composition and minor allele count in the target panel. Further details can be found at the start of this section.

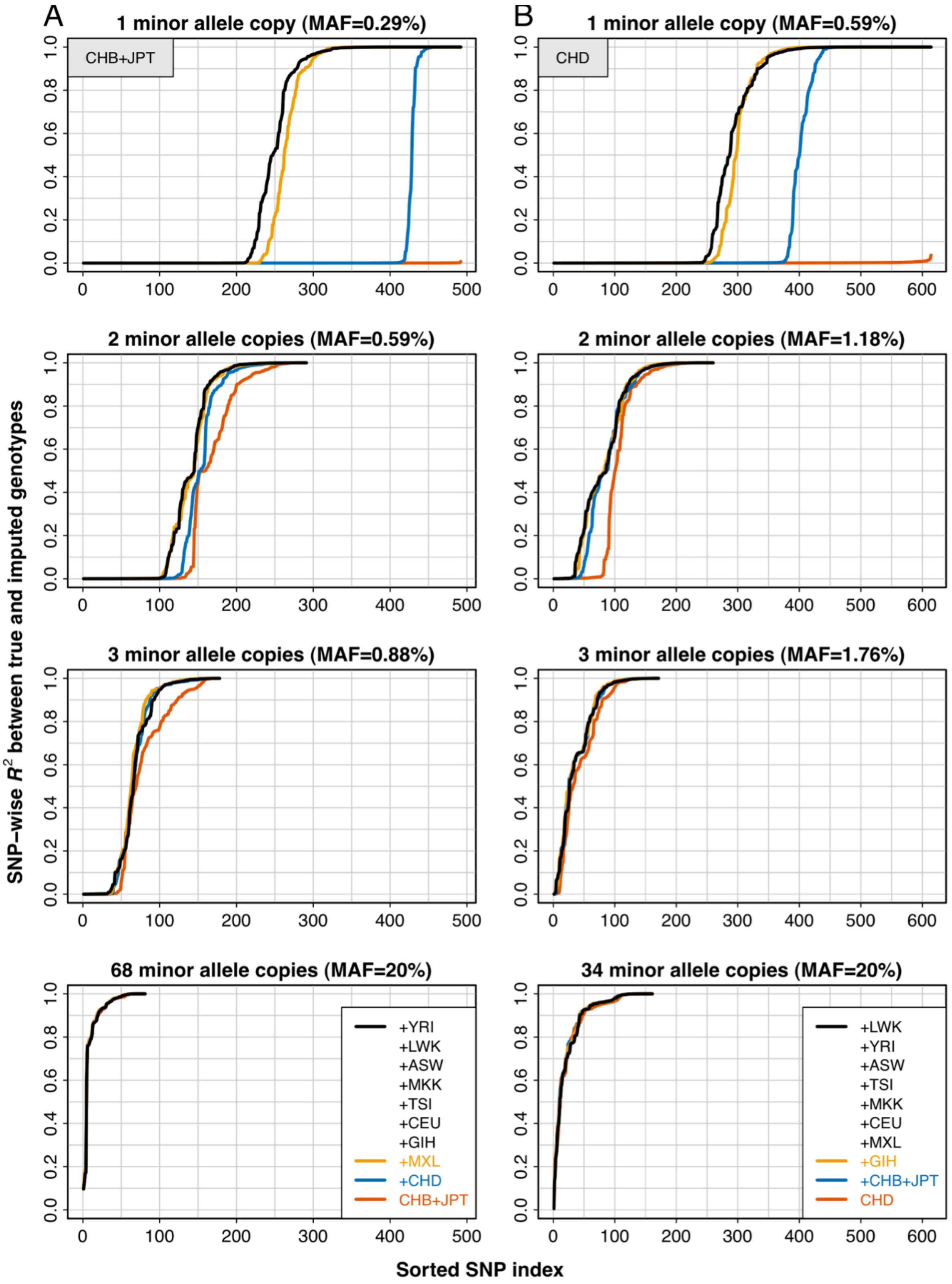

Figure S12 Cumulative distributions of SNP-wise imputation accuracy (R^2) in (A) CHB+JPT and (B) CHD, as a function of reference panel composition and minor allele count in the target panel. Further details can be found at the start of this section.

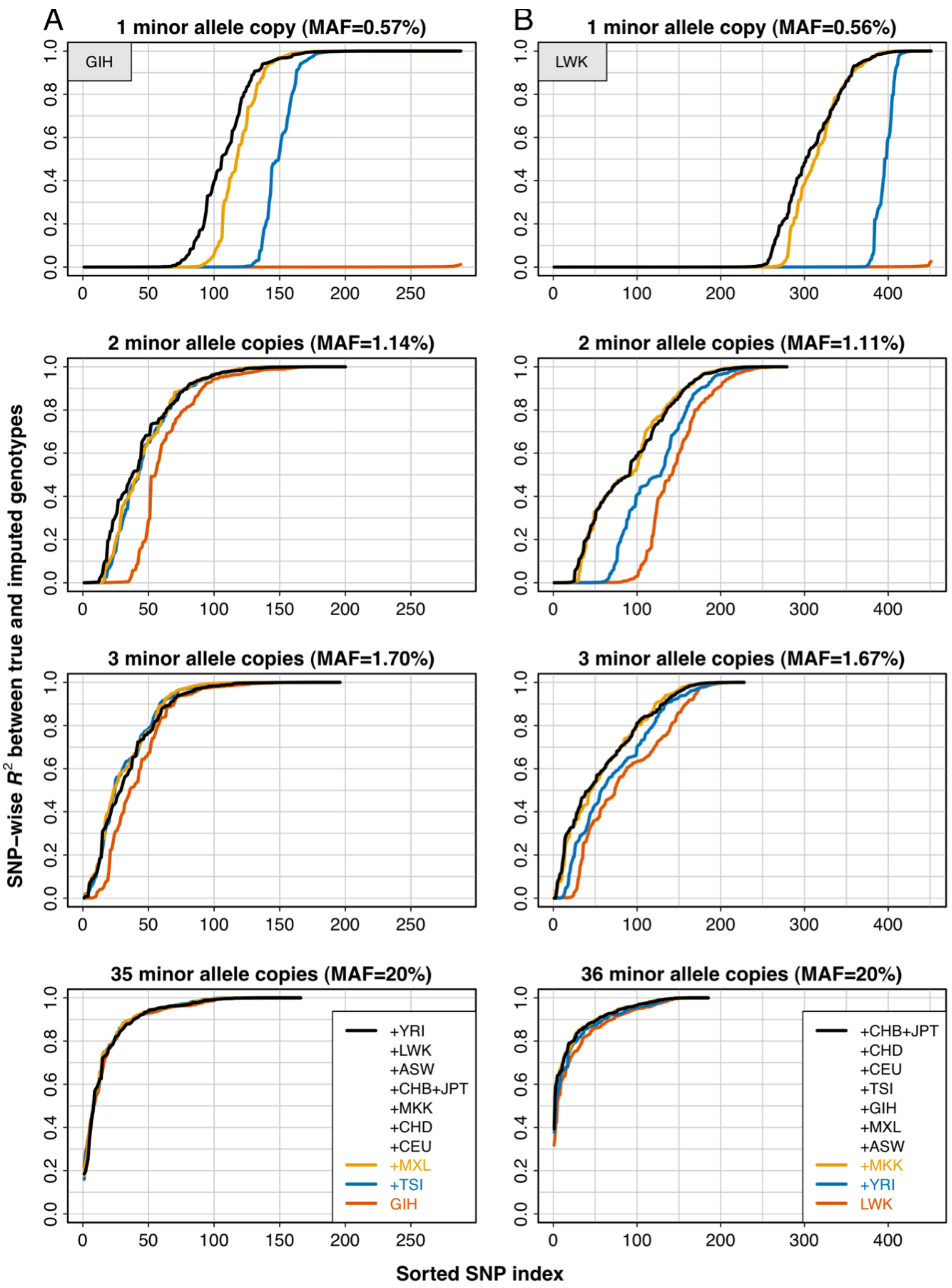

Figure S13 Cumulative distributions of SNP-wise imputation accuracy (R^2) in (A) GIH and (B) LWK, as a function of reference panel composition and minor allele count in the target panel. Further details can be found at the start of this section.

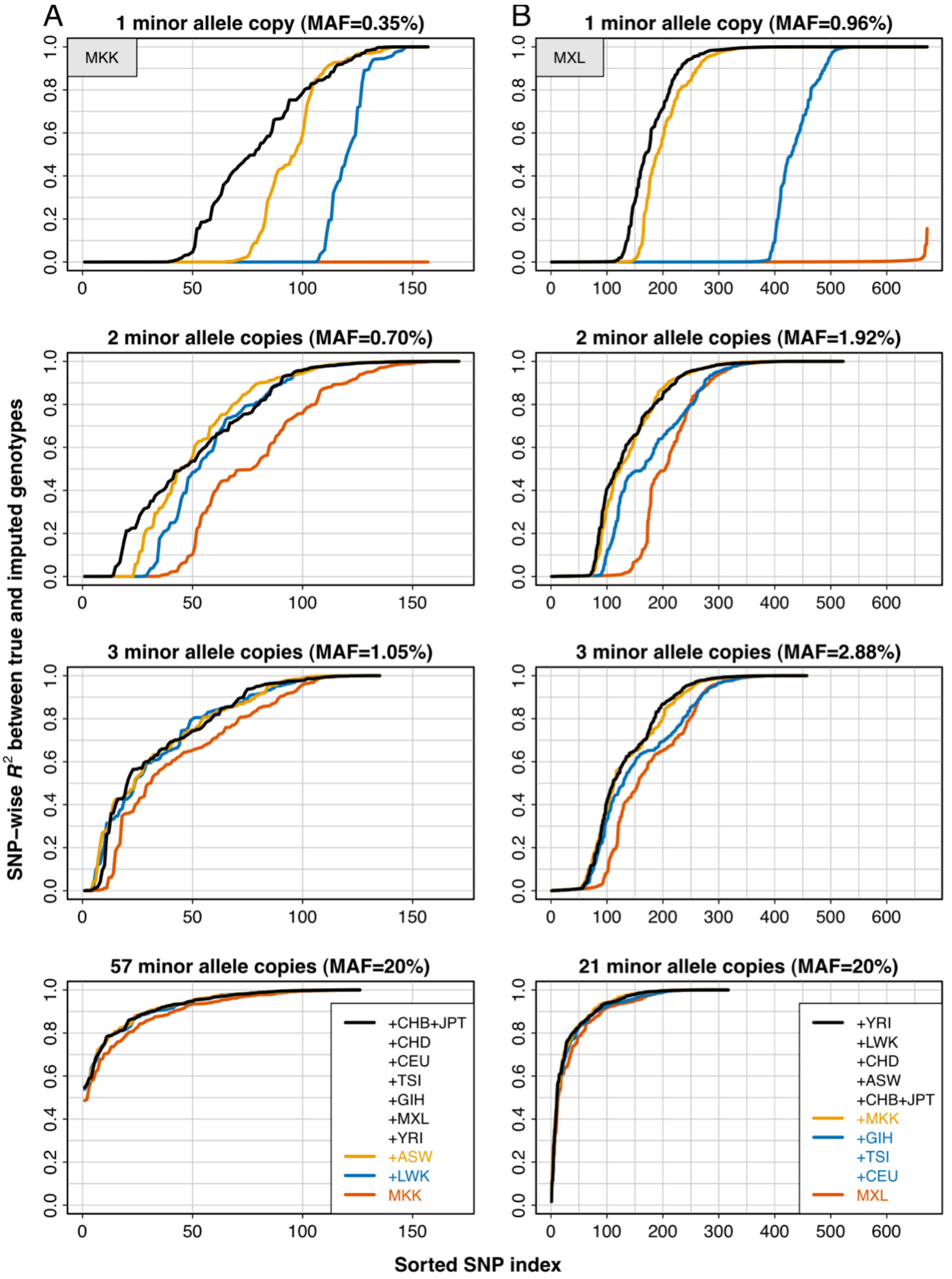

Figure S14 Cumulative distributions of SNP-wise imputation accuracy (R^2) in (A) MKK and (B) MXL, as a function of reference panel composition and minor allele count in the target panel. Further details can be found at the start of this section.

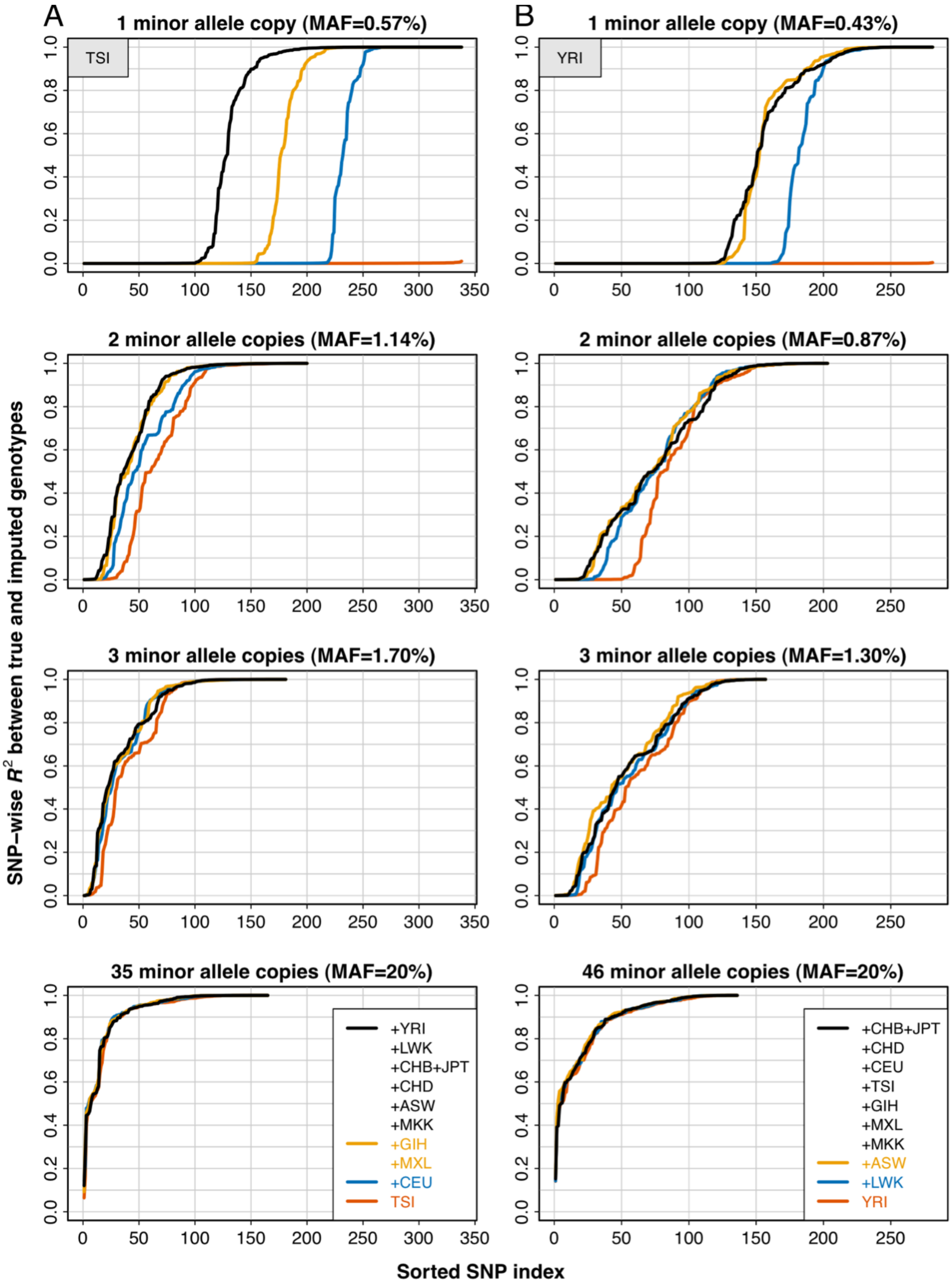

Figure S15 Cumulative distributions of SNP-wise imputation accuracy (R^2) in (A) TSI and (B) YRI, as a function of reference panel composition and minor allele count in the target panel. Further details can be found at the start of this section.

File S3

Figures S16-S18

These figures are similar to Figure 2 of the main text, except they are based on different observed SNP sets (Affymetrix 6.0 or Illumina 1M) and/or imputed SNP MAFs (low-frequency or common), as detailed in the figure captions. The plots show the imputation accuracy of IMPUTE2 and Beagle in all HapMap 3 cross-validation experiments. The accuracy of each experiment is plotted on the *y*-axis as the mean R^2 across all imputed SNPs in the specified frequency range in the cross-validation panel (identified by the grey box in each plot). The *x*-axis shows the k_{hap} parameter, which scales linearly with the computational burden of imputation updates in IMPUTE2. The solid black curves show how R^2 varies with k_{hap} when using IMPUTE2 with a reference panel containing the full set of 2,020 HapMap 3 haplotypes; the dashed black lines show the accuracy of Beagle with the same reference panel. IMPUTE2 was also applied to sub-panels of the full HapMap 3 panel, with results shown as orange curves.

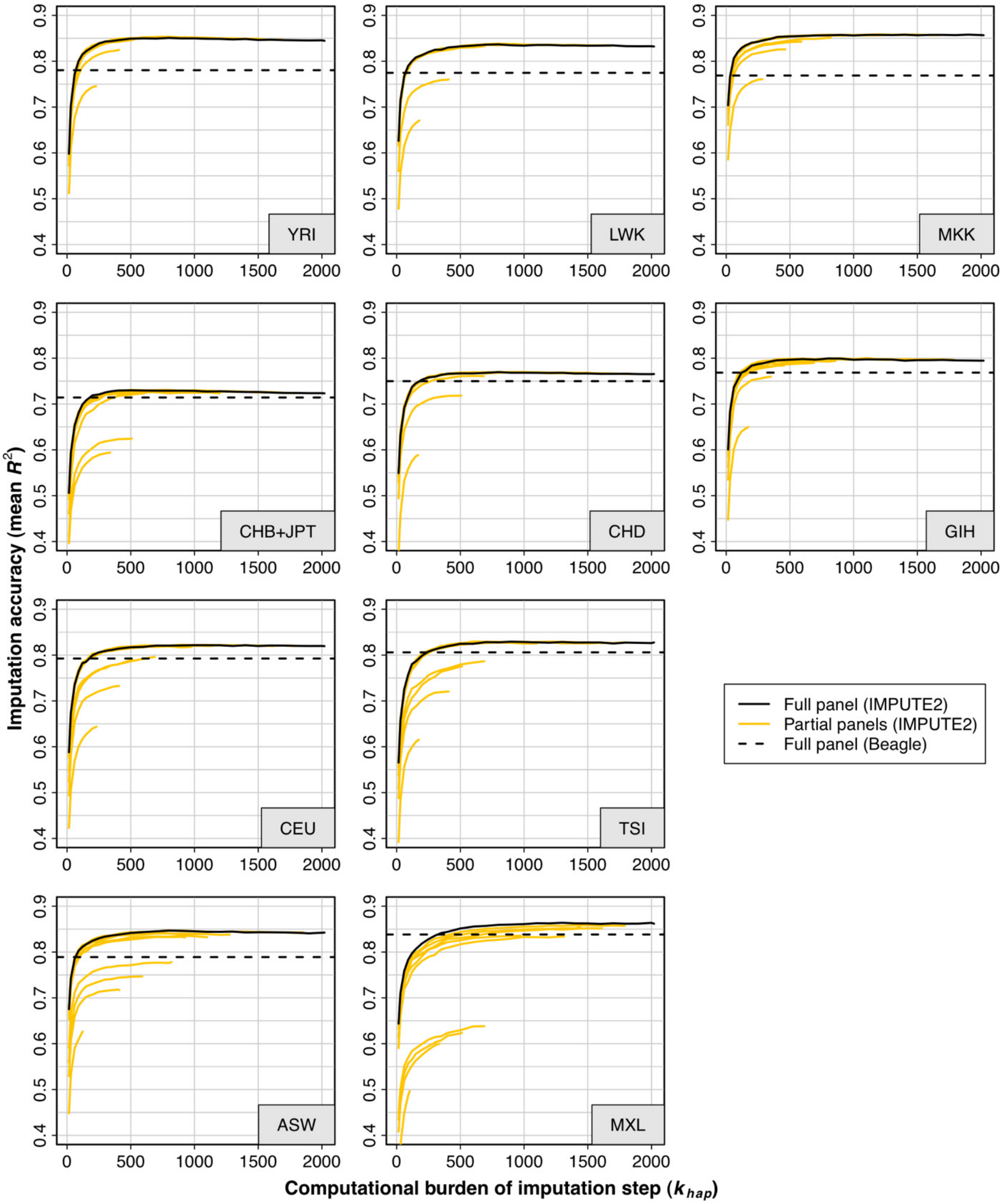

Figure S16 Imputation accuracy at low-frequency (MAF < 5%) SNPs imputed from observed Illumina 1M genotypes in HapMap 3 cross-validations, as a function of target panel, reference panel composition, k_{hap} value, and imputation method. Further details can be found at the start of this section.

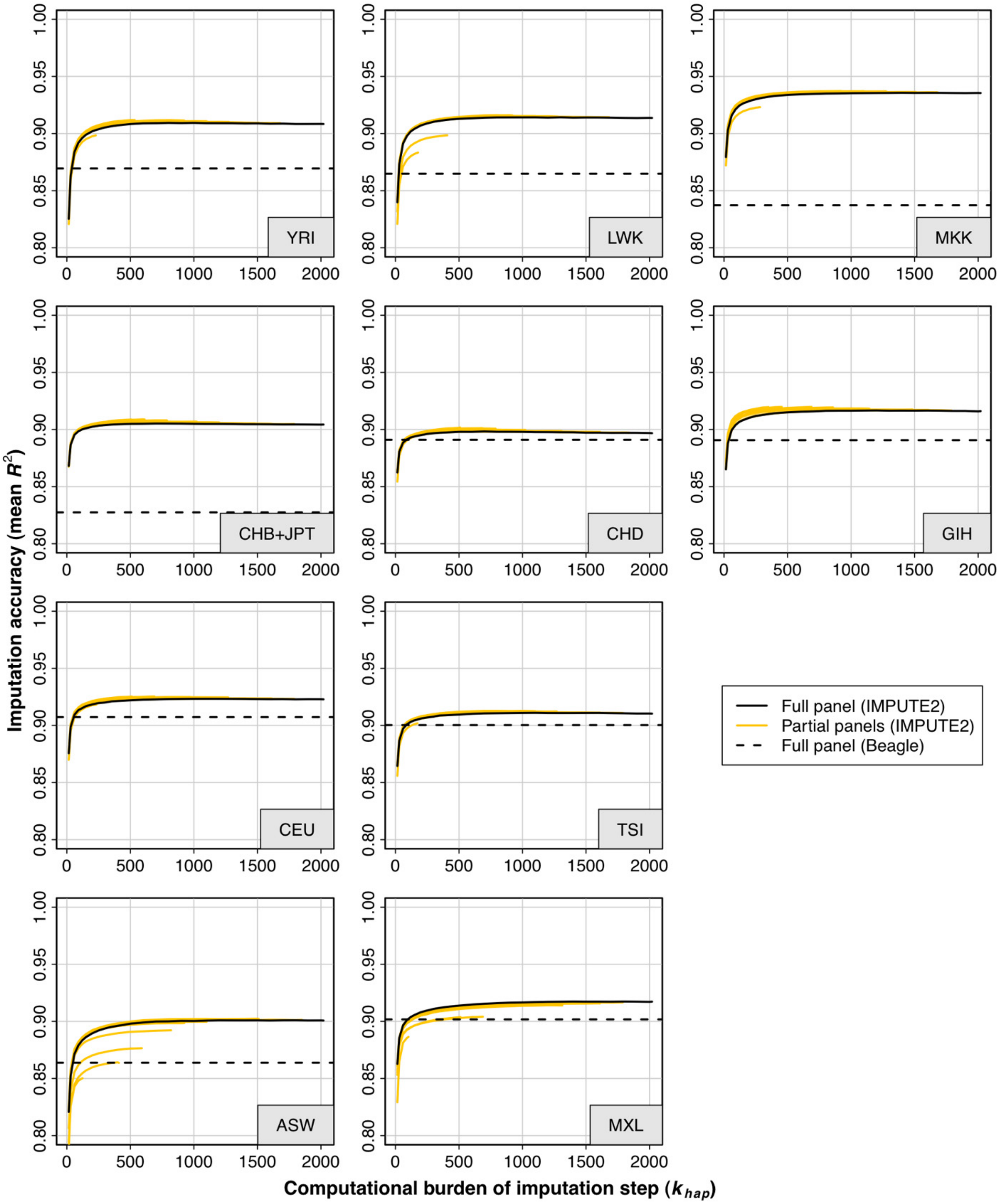

Figure S17 Imputation accuracy at common (MAF ≥ 5%) SNPs imputed from observed Affymetrix 6.0 genotypes in HapMap 3 cross-validations, as a function of target panel, reference panel composition, k_{hap} value, and imputation method. Further details can be found at the start of this section.

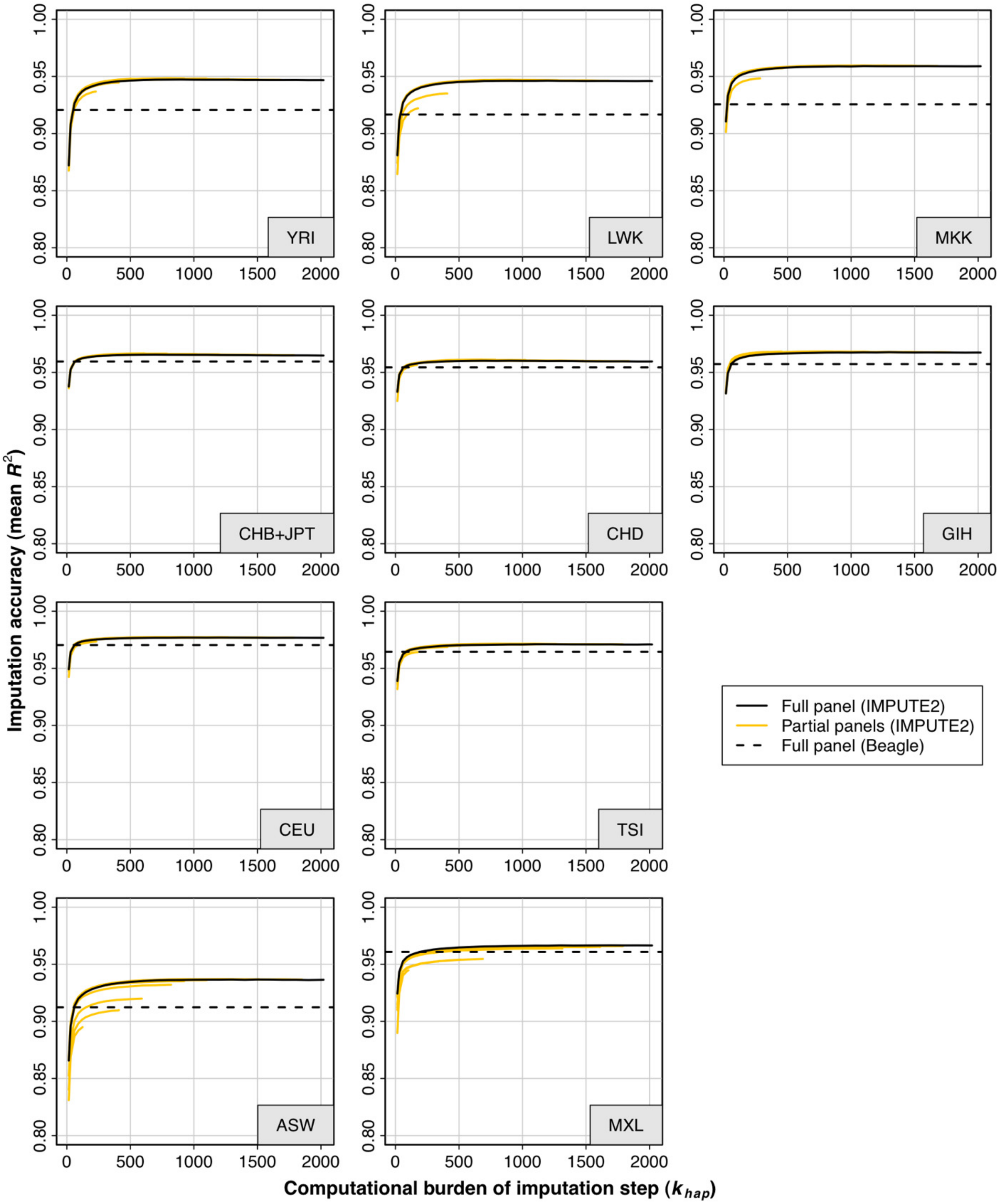

Figure S18 Imputation accuracy at common (MAF ≥ 5%) SNPs imputed from observed Illumina 1M genotypes in HapMap 3 cross-validations, as a function of target panel, reference panel composition, k_{hap} value, and imputation method. Further details can be found at the start of this section.

File S4

DISCREPANCY WITH IMPUTE2 VS. BEAGLE COMPARISON IN JOSTINS ET AL.

One aim of this paper is to compare our imputation framework (as implemented in IMPUTE2) with Beagle, which is another leading method for imputing from large reference panels. We found that IMPUTE2 was more accurate than Beagle in all populations considered. However, as noted in the Results section of the main text, a recent paper by Jostins et al. (2011) reached a different conclusion when comparing these two methods in a similar context. We believe that this discrepancy can be explained by spurious IMPUTE2 results in the Jostins et al. study, as we explain below.

As in our study, Jostins et al. ran IMPUTE2 and Beagle with a cosmopolitan HapMap 3 reference panel. Their cross-validation data comprised ∼1,300 controls sampled from the United Kingdom as part of the Wellcome Trust Case Control Consortium (WTCCC); the closest analogue in our study is the use of the HapMap 3 CEU panel as the target for imputation. Whereas we found that IMPUTE2 was more accurate than Beagle when imputing CEU data from a cosmopolitan HapMap 3 panel, Jostins et al. found that Beagle was more accurate than IMPUTE2 when imputing WTCCC data from the same reference panel: for SNPs with
MAF < 5%, our mean R^2 values were 0.739 and 0.716 for IMPUTE2 and Beagle, respectively, whereas their mean R^2 values were 0.833 and 0.873.

At face value, these results are not necessarily contradictory since they come from different target datasets (WTCCC vs. HapMap 3 CEU) and program settings (Jostins et al. used a smaller value of the *k* parameter in IMPUTE2, which should speed up the program at the cost of imputation accuracy). However, we also noticed other aspects of their results that disagreed with our expectations. For example, Jostins et al. ran IMPUTE version 1 (Marchini et al. 2007) on their data, and they found that it was consistently more accurate than IMPUTE2 ("IMPUTE version 2"), with an especially large difference at low-frequency SNPs. This was true even for ancestrally homogeneous and well-matched reference panels that contained too few haplotypes to activate IMPUTE2's k_{hap} approximation at the default value of 500.

These results contrast with our long experience as developers of both versions of IMPUTE: in a wide variety of datasets, we have always found that version 2 is slightly more accurate than version 1. The relative accuracies that Jostins et al. obtained for IMPUTE v1 and Beagle fit with our expectations – IMPUTE v1 was somewhat more accurate than Beagle, regardless of reference panel composition – which led us to believe that there might have been an issue with their IMPUTE2 runs.

Fortunately, in this case we had access to the same WTCCC data that Jostins et al. analyzed. To see whether our intuitions were correct, we repeated their imputation experiments as closely as possible (same reference panels, program versions, program settings, target chromosome, and genomic partitioning for parallel runs). Our accuracy numbers were

broadly similar to theirs, with the exception that IMPUTE2 became the most accurate method in almost all scenarios – exactly as we had anticipated.

It is hard to guess the mechanistic reasons for the relatively poor performance of IMPUTE2 in the Jostins et al. study. We downloaded the software from the web, just as any external researcher would do, and it worked well in our hands. Regardless, we believe it is safe to view the IMPUTE2 results from Jostins et al. as an anomaly, whereas the results in this study consistently show that IMPUTE2 is more accurate than Beagle when imputing from large, diverse reference panels.

File S5

POTENTIAL PROBLEMS IN MULTI-POPULATION REFERENCE SETS

Here we consider the kinds of problems that could arise from combining reference haplotypes from different populations. In this discussion, we will assume the availability of a reference panel with similar ancestry to the study data, and we will ask whether it would be harmful to augment this panel with haplotypes that are more diverged from the study population. When evaluating whether to include a particular set of haplotypes in the reference panel for a study, one should balance the chances that those haplotypes will contribute shared alleles to the study population against the chances that they will mislead an imputation algorithm. It is difficult to quantify these effects without extensive sequencing or accurate demographic models for the populations in question, but we can still discuss the tradeoffs between useful and misleading haplotypes in general terms.

Intuitively, allele sharing should decrease as the candidate haplotypes grow more diverged from the study population. Highly diverged reference haplotypes are easily identified and discarded by imputation models, so while they are unlikely to help imputation accuracy, they are also unlikely to hurt it. On the other hand, reference haplotypes that are closely related to the study population have the capacity to both help and mislead imputation methods. There are a few kinds of sites at which a set of candidate reference haplotypes could be misleading when added to a well-matched panel:

1. Sites that are monomorphic in the study dataset but polymorphic in the reference haplotypes. These could cause the imputation of alleles that do not exist in the study data.
2. Sites that are polymorphic in the study dataset but monomorphic in the reference haplotypes. These could dilute the imputation signal if the haplotype background of the variant allele is present in the reference set.
3. Sites that have experienced recurrent mutation, such that the study population and reference haplotypes carry the same variant allele on different haplotype backgrounds. These could cause imputed false alleles, missed true alleles, or low-confidence imputation, depending on the relative frequencies of the mutations.

The prevalence of these scenarios will depend on a complex mixture of factors such as sample size, allele frequency, divergence time, migration rates, and properties of the SNPs typed in the study individuals. We can evaluate the respective scenarios in light of their potential effects on power in an association study:

1. Imputed, non-existent alleles clearly do not increase power, but we argue that they are also unlikely to create

false associations. For this to happen, the variant alleles in the reference panel would have to correlate strongly with the phenotype in an association study after applying standard stratification corrections, and this seems unlikely to happen in a well-designed study. We looked at these kinds of sites in our HapMap 3 cross-validations, and we found that variant alleles were almost never imputed into panels that did not carry those alleles (data not shown). Hence, this class of sites neither helps nor hurts the association testing.

2. Diluted imputation signals can hurt the power to detect associations at these sites. Signal dilution is most likely to occur in closely related populations that share haplotype backgrounds and that lack the flanking mutations to distinguish these backgrounds. (Note that "distinguishing mutations" must be assayed on a study's genotyping platform if they are to discriminate similar haplotype backgrounds.) Fortunately, this effect is self-limiting since the more closely two populations are related, the rarer an allele would tend to be if it were present in one population but not the other. We hypothesize that these kinds of alleles will typically be too rare to have phenotypic effects that are detectable in single-marker association tests.
3. Recurrent mutations should cause much the same effects as the diluted imputation signals discussed above, and they should be subject to the same frequency limitations. Previous work has shown that of the alleles which occur at low frequency in multiple populations, only a small fraction show evidence of recurrent mutation (The International HapMap Consortium 2010).

While quantifying these statements is an important and open area of research, we expect that the worldwide reference set being collected by the 1,000 Genomes Project will be mostly immune to these problems, which motivates our suggestion to use inclusive reference sets from that resource. Rare genomic phenomena (e.g., the well-known positive selection on recurrent mutations in the beta-globin gene) will create occasional exceptions to the trends discussed here, but the regions that could harbor these kinds of events can probably be identified ahead of time and given special treatment. We also note that negative effects of misleading haplotypes will be mitigated by the continuing development of SNP arrays targeted at low-frequency variation. Looking ahead, it will be important to reevaluate the merits of combining panels in future reference datasets, including dedicated reference data collected in fine-mapping experiments or exome sequencing studies.

File S6

RELATIONSHIP BETWEEN k_{hap} AND A COALESCENT-BASED APPROXIMATION

While we were preparing this manuscript for publication, another group published a method for choosing a custom reference panel for each study individual in each part of the genome (Pasaniuc et al. 2010). Like our k_{hap} approximation, their "coalescent-based" approach is motivated by genealogical reasoning, and the methods share other features: they both eliminate reference haplotypes that differ by too many alleles from a study haplotype of interest, and they are both designed to handle multi-population reference panels and admixed individuals.

However, there are important differences in the motivations for these approximations. Aside from accounting for changes in local ancestry, the other main goal of the Pasaniuc et al. method is to increase imputation accuracy by re-weighting the reference haplotypes with probabilities derived from coalescent theory. Their method essentially looks at the data and then modifies the probability that a given study individual will copy each reference haplotype; reference haplotypes that show high identity with the study genotypes are upweighted, thereby increasing the chances that an imputation method will copy them. IMPUTE2 also uses coalescent arguments to weight reference haplotypes, but in our case the weights are built into the underlying statistical model (which was originally described by Li and Stephens (2003)) and can be obtained with a uniform prior on copying each reference haplotype. Our k_{hap} approximation can be seen as changing these weights, in the sense that it rounds to zero the copying probabilities of haplotypes separated by many allele differences, but it assumes a uniform prior probability of copying the remaining k_{hap} haplotypes. This approximation does not aim to change the haplotype weights; its goal is to maintain the relative weights under the model while avoiding expensive HMM calculations on reference haplotypes that will contribute little to the genotype imputation probabilities.

Fundamentally, the Pasaniuc et al. approximation is designed to increase imputation accuracy (usually at increased computational cost), whereas our approximation is designed to maintain accuracy while reducing computation (although, as we saw in the Results, it may slightly improve accuracy in some situations). For investigators running imputation methods, accuracy and efficiency are competing demands. Imputing a large GWAS requires substantial computing power, so any method that claims to increase accuracy must produce large enough gains to justify the computational cost.

To assess this tradeoff, we implemented the Pasaniuc et al. method and tried it with various settings in our HapMap 3 comparisons. As suggested, we used their approximation to create local reference panels for each study individual and reference SNP, then ran IMPUTE2 with no k_{hap} restriction. Our preliminary experiments showed that the haplotype re-weighting scheme was both slower (as expected) *and* less accurate than IMPUTE2 on default settings. This was true even for admixed datasets, where local re-weighting is meant to excel. On the basis of these initial results, we decided not to pursue the Pasaniuc

et al. method further.

We acknowledge that the Pasaniuc et al. approximation could, in principle, improve accuracy by introducing a notion of time depth in the coalescent process; Stephens and Scheet (2005) used a similar idea to develop an extension of the Li and Stephens model. Hence, the poor performance of the Pasaniuc et al. approximation in our experiments could reflect a failure to locate the optimal parameters of the model, which might depend on reference panel size, the SNP density and content of a GWAS dataset, the allele frequency of the SNP being imputed, and other factors. Alternatively, our results could imply that the method inherently works less well in reference panels like HapMap 3 than in the smaller panels on which it was originally tested, as was recently suggested by some of the authors of the Pasaniuc et al. method (Pasaniuc et al. 2011).

Regardless of the true reasons for these results, we find it instructive that we, as well-informed users, could not get the Pasaniuc et al. method to produce high accuracy on our data. Perhaps future developments will make the method easier to use in a variety of situations, but in the meantime we suggest that the k_{hap} approximation within IMPUTE2 offers many practical advantages for GWAS investigators.

REFERENCES

1. L. Jostins, K. I. Morley, and J. C. Barrett. Imputation of low-frequency variants using the HapMap3 benefits from large, diverse reference sets. *Eur. J. Hum. Genet.*, 19:662–666, Jun 2011.
2. Na Li and Matthew Stephens. Modeling linkage disequilibrium and identifying recombination hotspots using single-nucleotide polymorphism data. *Genetics*, 165(4):2213–2233, Dec 2003.
3. B. Pasaniuc, R. Avinery, T. Gur, C. F. Skibola, P. M. Bracci, and E. Halperin. A generic coalescent-based framework for the selection of a reference panel for imputation. *Genet. Epidemiol.*, 34: 773–782, Dec 2010.
4. B. Pasaniuc, N. Zaitlen, G. Lettre, G. K. Chen, A. Tandon, W. H. Kao, I. Ruczinski, M. Fornage, D. S. Siscovick, X. Zhu, et al. Enhanced Statistical Tests for GWAS in Admixed Populations: Assessment using African Americans from CARe and a Breast Cancer Consortium. *PLoS Genet.*, 7:e1001371, Apr 2011.
5. Matthew Stephens and Paul Scheet. Accounting for decay of linkage disequilibrium in haplotype inference and missing-data imputation. *Am J Hum Genet*, 76(3):449–462, Mar 2005.
6. The International HapMap Consortium. Integrating common and rare genetic variation in diverse human populations. *Nature*, 467:52–58, Sep 2010.

Table S1 Number of low-frequency SNPs imputed in each HapMap 3 panel from Affymetrix 6.0 SNPs

Panel	Number of SNPs with MAF < 5%
ASW	1870
CEU	1579
CHB+JPT	2206
CHD	1882
GIH	1667
LWK	2230
MKK	1925
MXL	2364
TSI	1523
YRI	1844

Table S2 Discovery probabilities for SNPs by variant allele count in simulated reference panels

Variant allele count of SNP in reference set	Probability of discovery
1	0.25
2	0.50
3	0.74
4	0.87
5	0.90
6	0.93
7	0.97
8	0.98
9	0.98
10+	0.99
