# Supplementary material for: Genotype Imputation with Thousands of Genomes
Source: G3 (Bethesda). 2011 Nov 1;1(6):457–70. doi: 10.1534/g3.111.001198 (PMC3276165; doi:10.1534/g3.111.001198)
Supplement: Supporting Information [file supp_1.6.457_FileS1.pdf]

File S1

Figures S1-S10

These figures show detailed results from cross-validation experiments in HapMap 3. A set of SNPs was masked in one individual at a time, then imputed with various reference panels and k_{hap} settings. Each masked individual was provided with observed genotypes at Affymetrix 6.0 SNPs. The accuracy of each experiment is plotted on the *y*-axis as the mean R^2 across all imputed SNPs with MAF < 5% in the cross-validation panel (identified by the grey box in each figure). The *x*-axis shows the k_{hap} parameter, which scales linearly with the computational burden of imputation updates in IMPUTE2. Each figure contains results for a single HapMap 3 cross-validation panel, in two parts:

(A) These plots are similar to the ones shown in Figure 1 of the main text. Each curve represents a different reference panel, with panels added cumulatively in the order shown in the legend, reading from bottom to top. All results are from IMPUTE2.

(B) These plots are identical to the ones shown in Figure 2 of the main text. Results from the full HapMap 3 reference panel of 2,020 haplotypes are represented in black (solid line for IMPUTE2, dashed line for Beagle), and results from sub-panels of the HapMap 3 set are shown in orange (IMPUTE2 only). This way of displaying the results highlights our proposed strategy, which is to use all available reference haplotypes for imputation in every population.

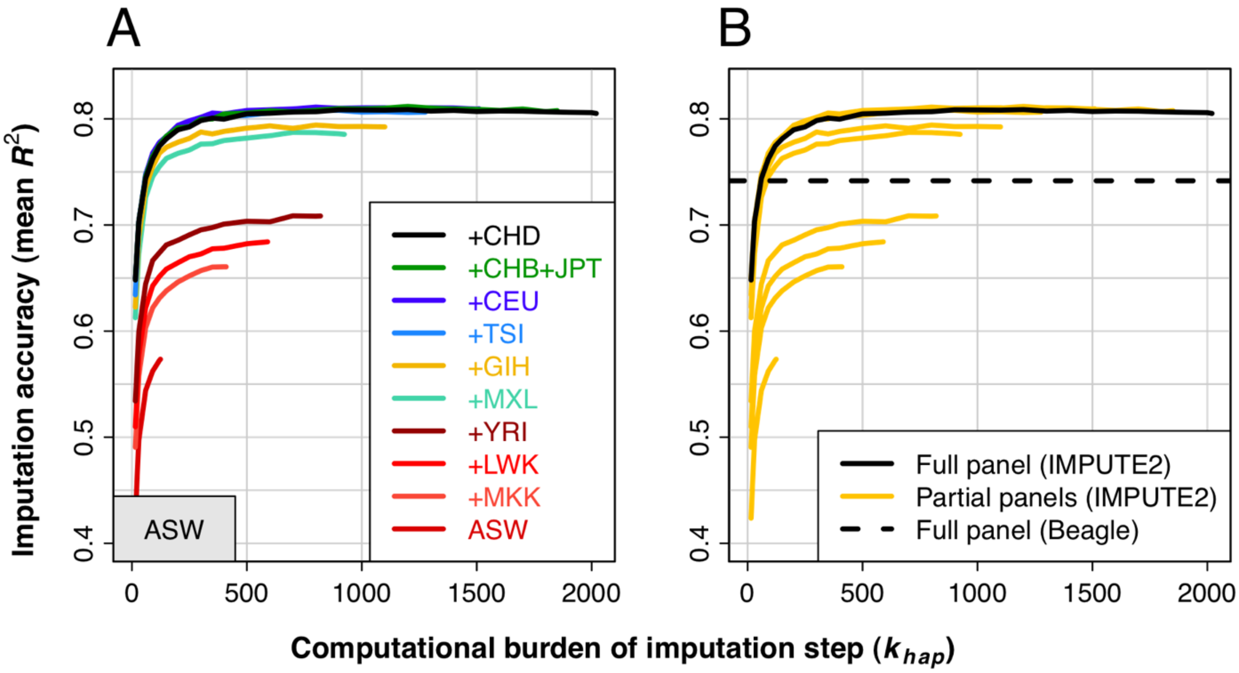

Figure S1 Imputation accuracy at low-frequency SNPs in HapMap 3 cross-validations in ASW, as a function of reference panel composition, k_{hap} value, and imputation method. Further details can be found at the start of this section.

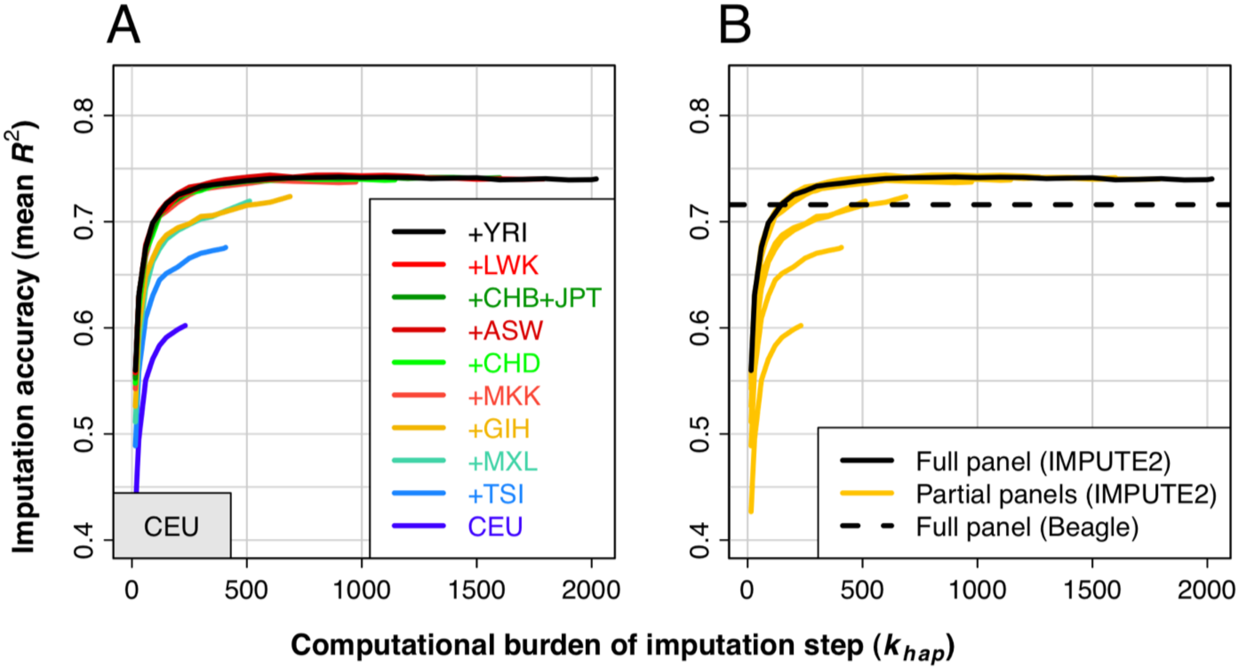

Figure S2 Imputation accuracy at low-frequency SNPs in HapMap 3 cross-validations in CEU, as a function of reference panel composition, k_{hap} value, and imputation method. Further details can be found at the start of this section.

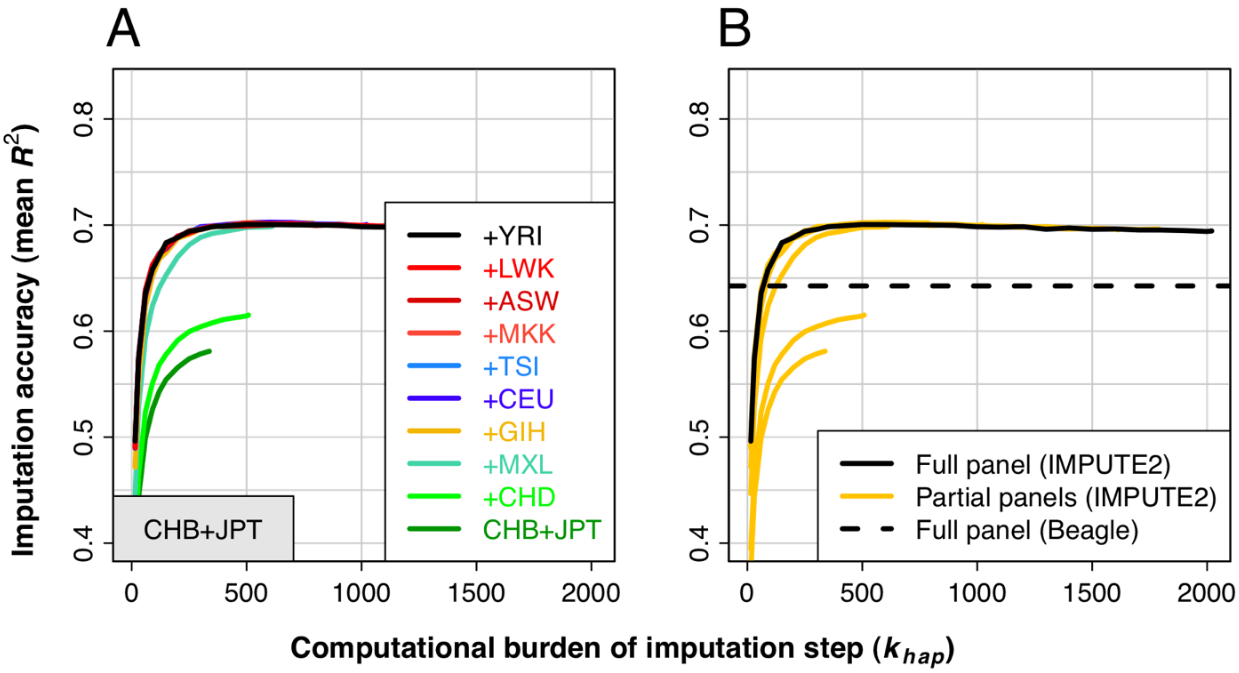

Figure S3 Imputation accuracy at low-frequency SNPs in HapMap 3 cross-validations in CHB+JPT, as a function of reference panel composition, k_{hap} value, and imputation method. Further details can be found at the start of this section.

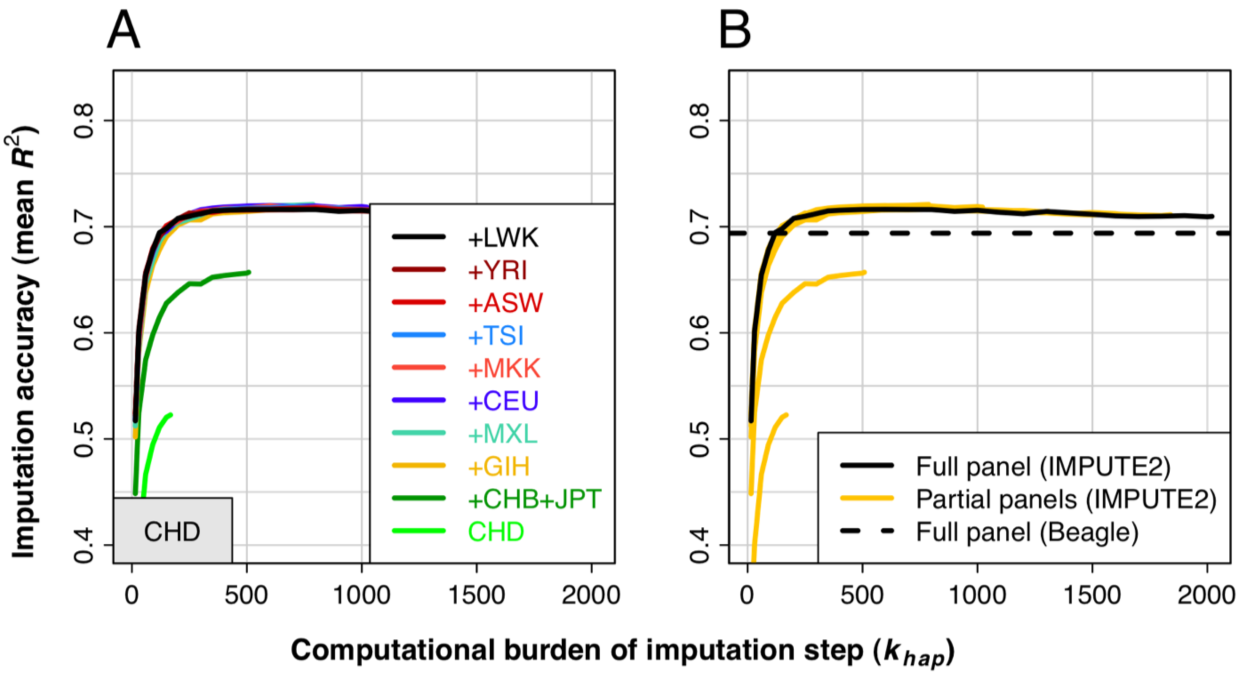

Figure S4 Imputation accuracy at low-frequency SNPs in HapMap 3 cross-validations in CHD, as a function of reference panel composition, k_{hap} value, and imputation method. Further details can be found at the start of this section.

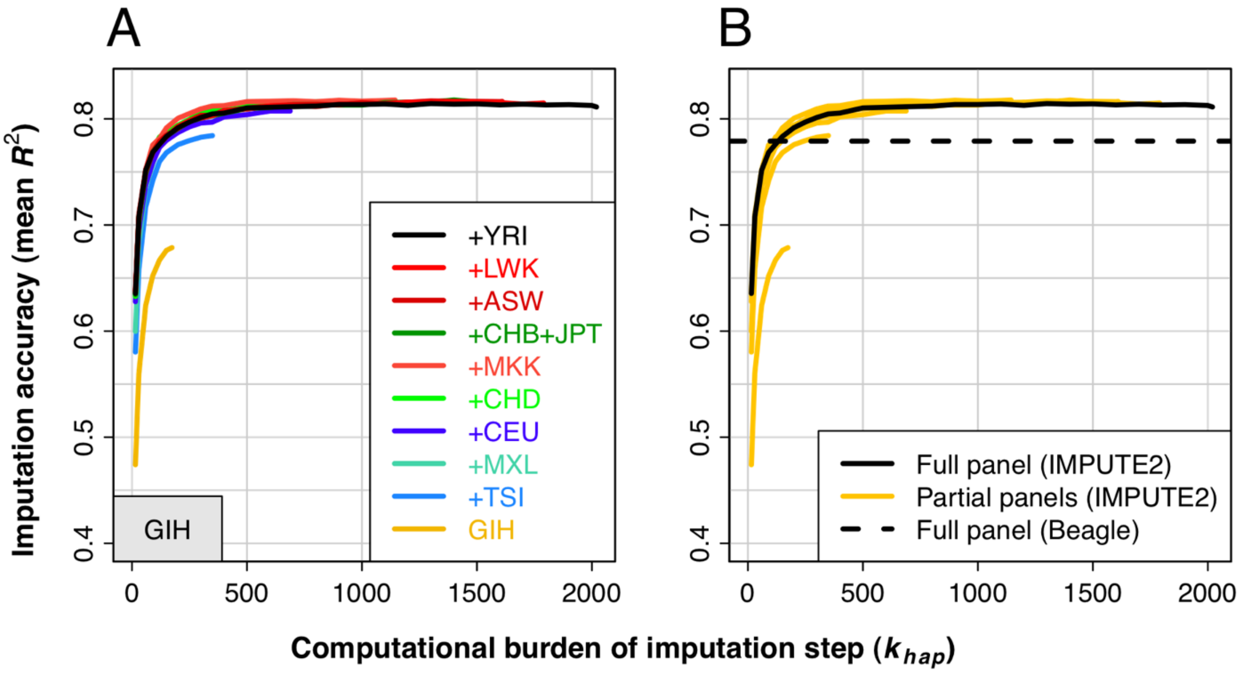

Figure S5 Imputation accuracy at low-frequency SNPs in HapMap 3 cross-validations in GIH, as a function of reference panel composition, k_{hap} value, and imputation method. Further details can be found at the start of this section.

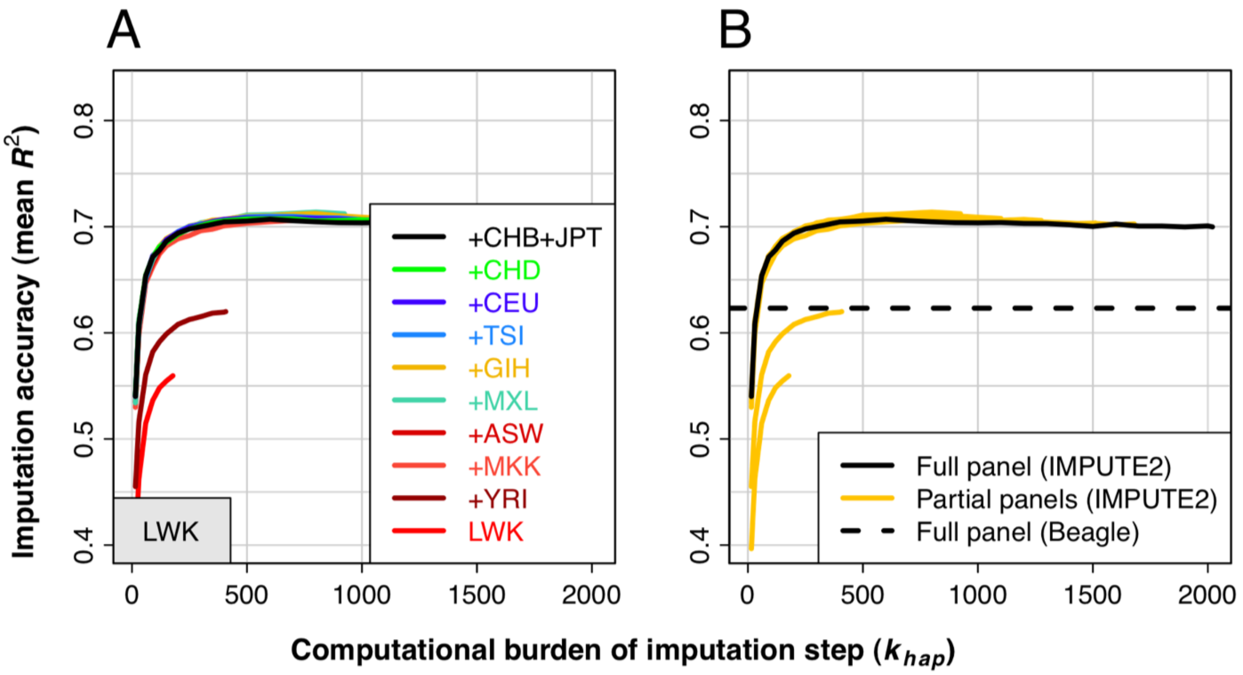

Figure S6 Imputation accuracy at low-frequency SNPs in HapMap 3 cross-validations in LWK, as a function of reference panel composition, k_{hap} value, and imputation method. Further details can be found at the start of this section.

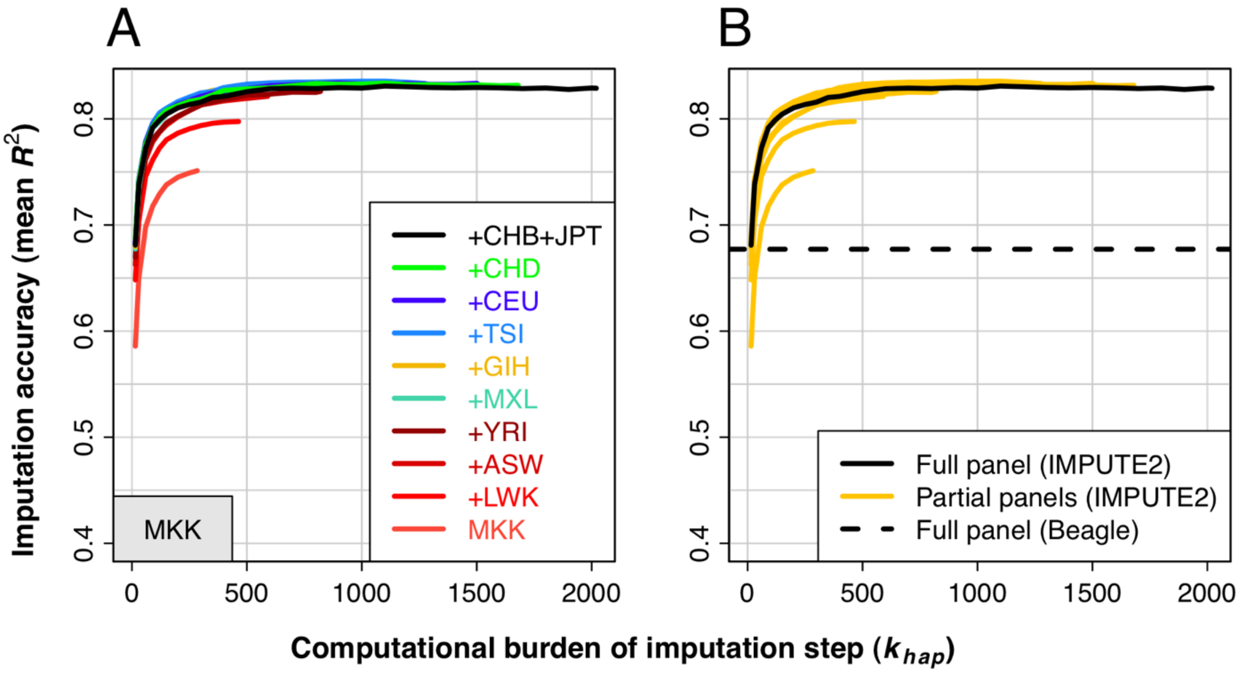

Figure S7 Imputation accuracy at low-frequency SNPs in HapMap 3 cross-validations in MKK, as a function of reference panel composition, k_{hap} value, and imputation method. Further details can be found at the start of this section.

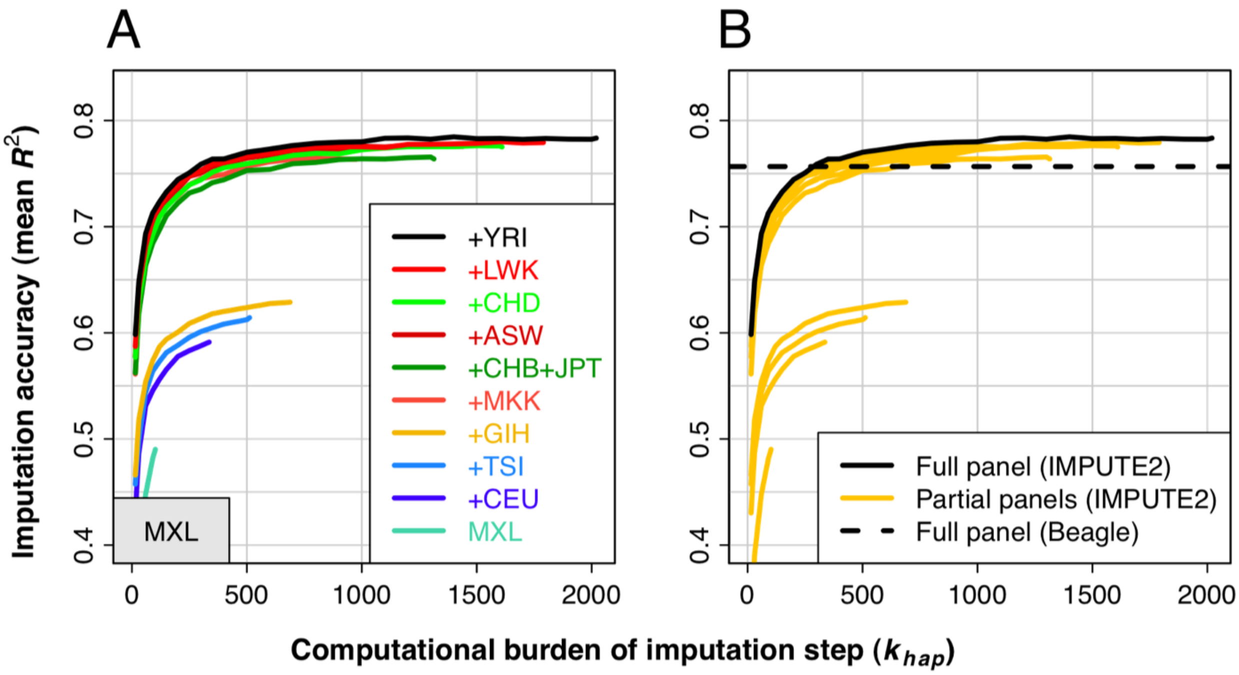

Figure S8 Imputation accuracy at low-frequency SNPs in HapMap 3 cross-validations in MXL, as a function of reference panel composition, k_{hap} value, and imputation method. Further details can be found at the start of this section.

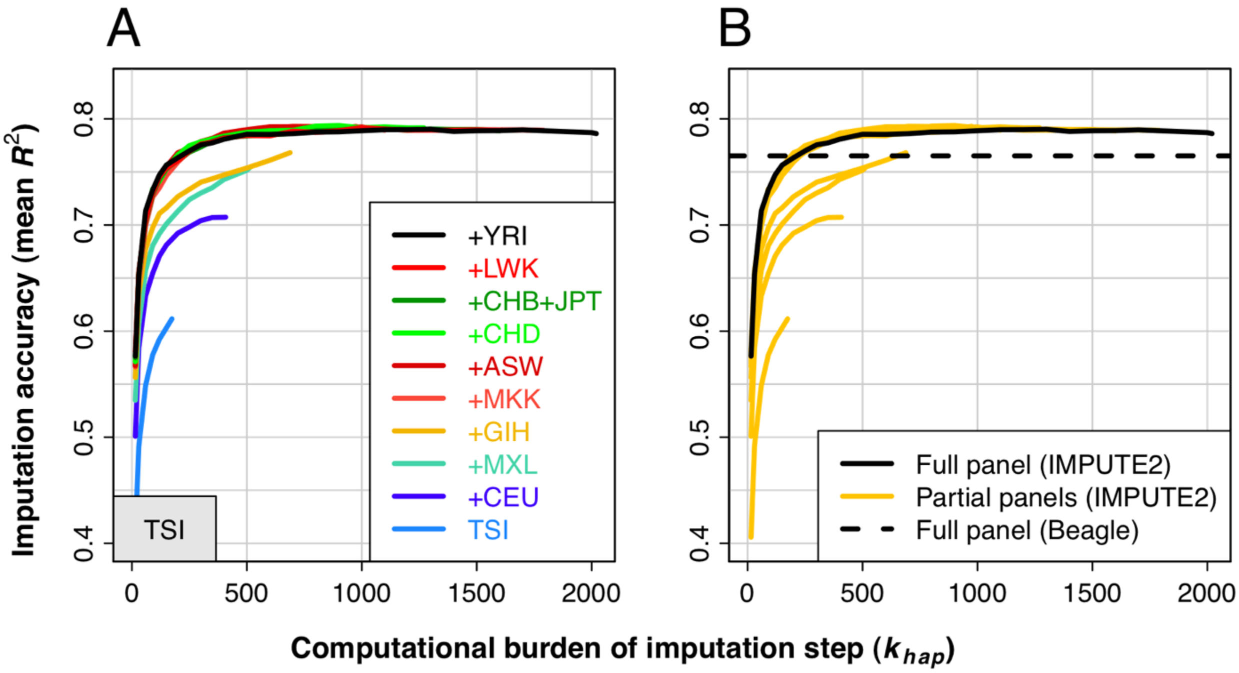

Figure S9 Imputation accuracy at low-frequency SNPs in HapMap 3 cross-validations in TSI, as a function of reference panel composition, k_{hap} value, and imputation method. Further details can be found at the start of this section.

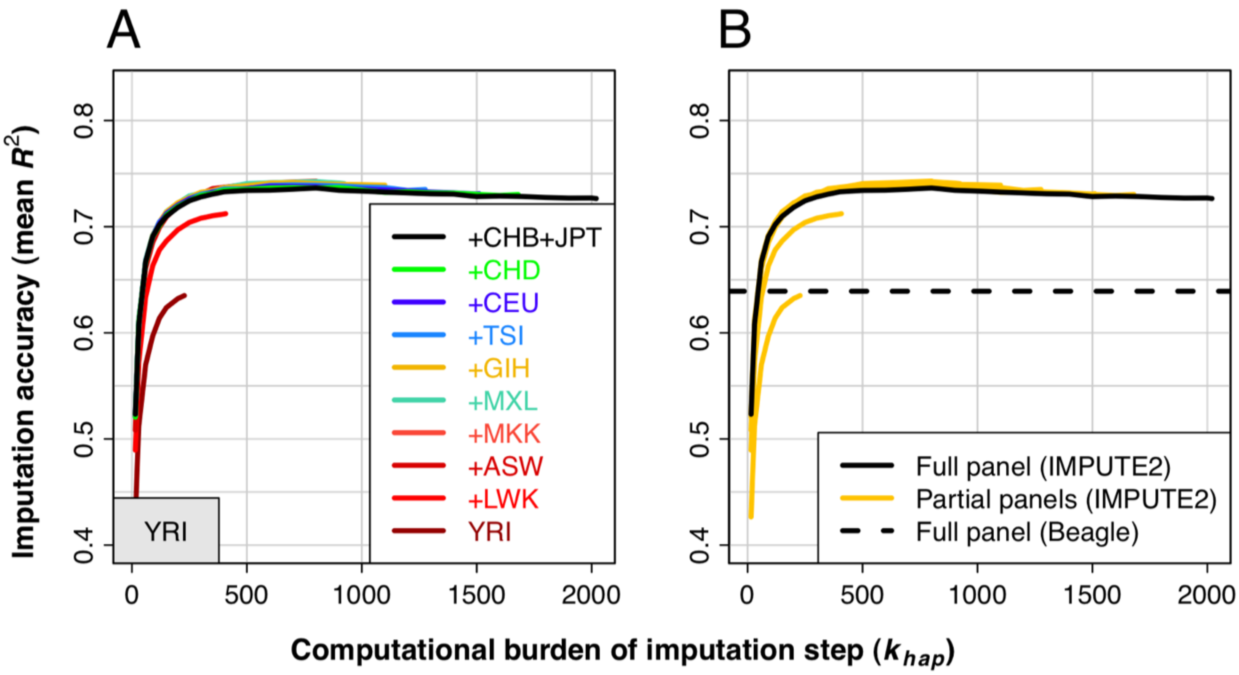

Figure S10 Imputation accuracy at low-frequency SNPs in HapMap 3 cross-validations in YRI, as a function of reference panel composition, k_{hap} value, and imputation method. Further details can be found at the start of this section.
