# Supplementary material for: Genotype Imputation with Thousands of Genomes
Source: G3 (Bethesda). 2011 Nov 1;1(6):457–70. doi: 10.1534/g3.111.001198 (PMC3276165; doi:10.1534/g3.111.001198)
Supplement: Supporting Information [file supp_1.6.457_FileS2.pdf]

File S2

Figures S11-S15

These figures provide a deeper explanation of the results presented in Figures S1-S10. For each HapMap 3 panel, we took slices of the IMPUTE2 accuracy curves in those figures at k_{hap} = 500, and we expanded the mean R^2 values to full cumulative distributions of SNP-wise R^2 values.

Each column corresponds to a single cross-validation target panel from HapMap 3, as labeled by the grey box in the topmost plot. Reading from top to bottom, the plots display R^2 distributions for SNPs with 1, 2, 3, and N copies of the minor allele in the target panel, where N is the number that corresponds to MAF = 20% in that panel. The different curves within a plot show the R^2 distributions for reference panels with various levels of inclusiveness, with red representing imputation within the target panel and black representing imputation from the full HapMap 3 panel. We allocated the intermediate colors (blue and orange) separately for each target panel to capture interesting features; the composite reference panels they represent can be determined by reading the plot legends from bottom to top.

The topmost plots in each figure show the results for SNPs with a single minor allele copy (singletons). These SNPs are a special case: in a leave-one-out analysis within the target panel, it should be impossible to correctly impute a singleton allele since each such allele is removed from the reference panel when the individual carrying it is masked. This scenario models alleles that exist in a population at low frequency but were not sampled in a population-specific reference panel. The red curves in the topmost plots are concentrated almost entirely at R^2 = 0 since a variant allele cannot be imputed when it is not represented in the reference panel; non-zero values in the other curves correspond to alleles that were “rescued” through the inclusion of reference panels from other populations. (Note that the red curves occasionally achieve non-zero R^2 values, as in the ASW panel, through stochastic fluctuations in the imputation.)

These results confirm that ancestrally diverse reference panels are most beneficial at SNPs with low MAFs, whereas common SNPs are imputed well regardless of panel composition. Our pro- posed strategy of using all available reference haplotypes leads to the highest accuracy levels in most of these plots.

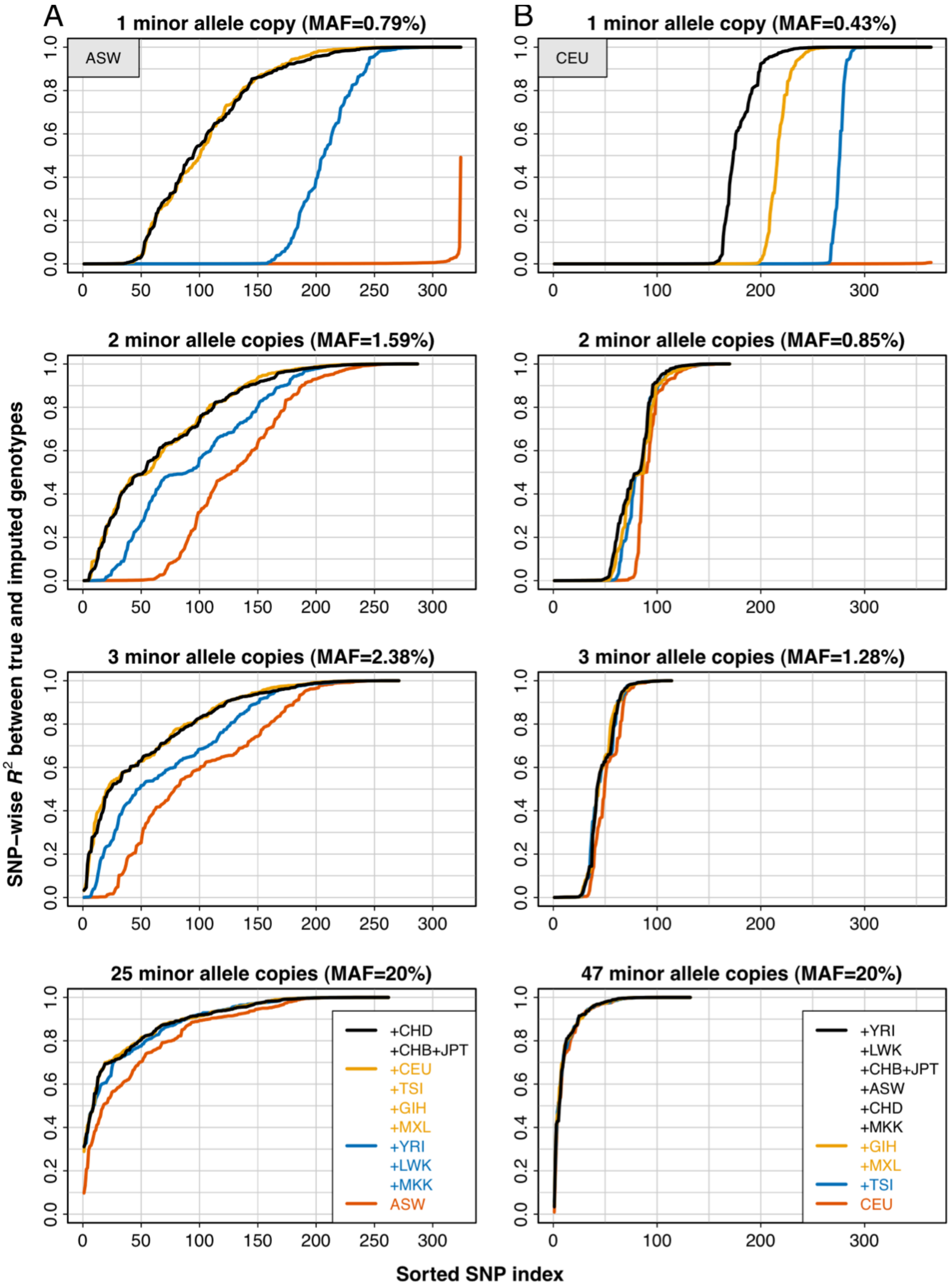

Figure S11 Cumulative distributions of SNP-wise imputation accuracy (R^2) in (A) ASW and (B) CEU, as a function of reference panel composition and minor allele count in the target panel. Further details can be found at the start of this section.

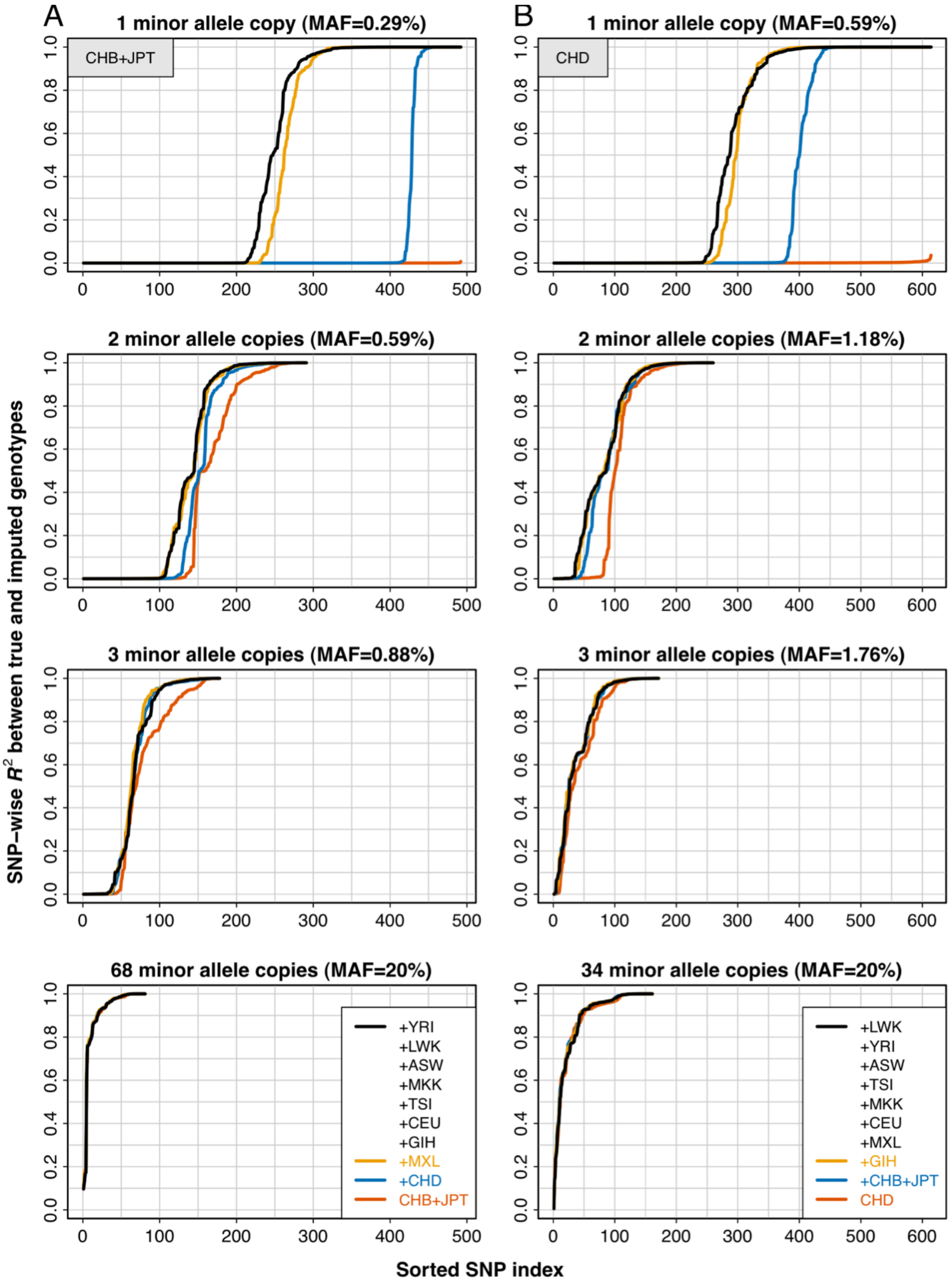

Figure S12 Cumulative distributions of SNP-wise imputation accuracy (R^2) in (A) CHB+JPT and (B) CHD, as a function of reference panel composition and minor allele count in the target panel. Further details can be found at the start of this section.

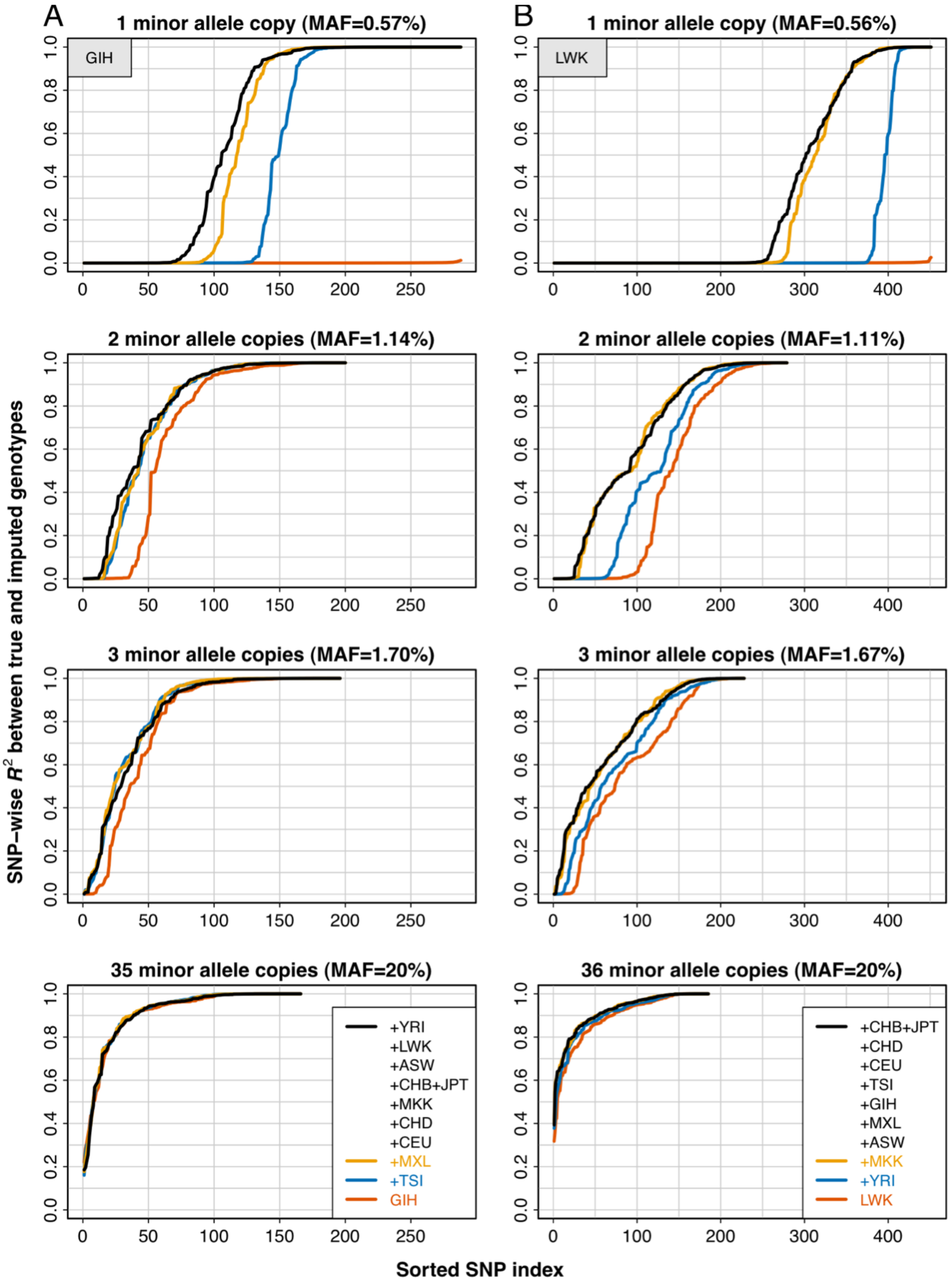

Figure S13 Cumulative distributions of SNP-wise imputation accuracy (R^2) in (A) GIH and (B) LWK, as a function of reference panel composition and minor allele count in the target panel. Further details can be found at the start of this section.

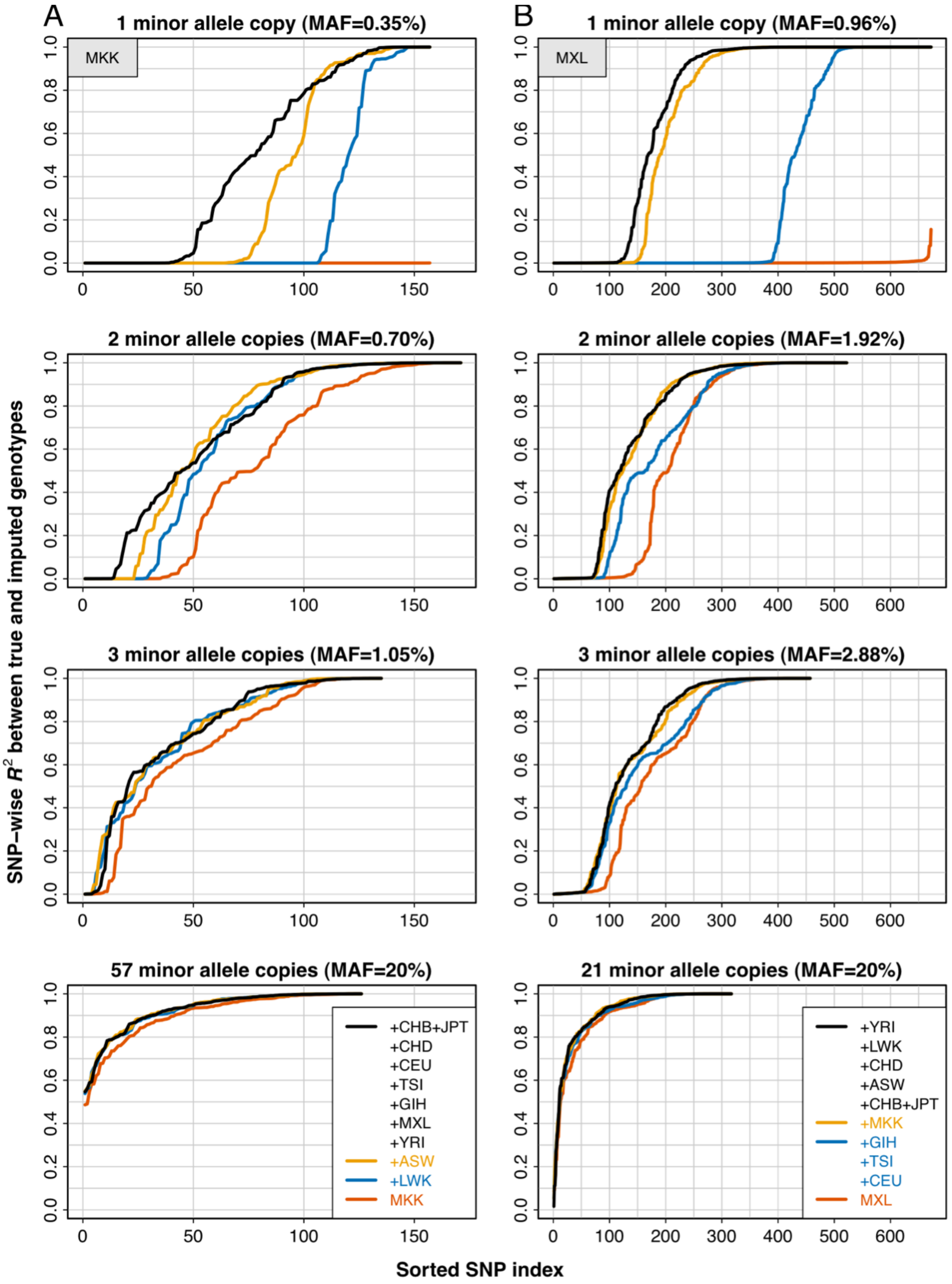

Figure S14 Cumulative distributions of SNP-wise imputation accuracy (R^2) in (A) MKK and (B) MXL, as a function of reference panel composition and minor allele count in the target panel. Further details can be found at the start of this section.

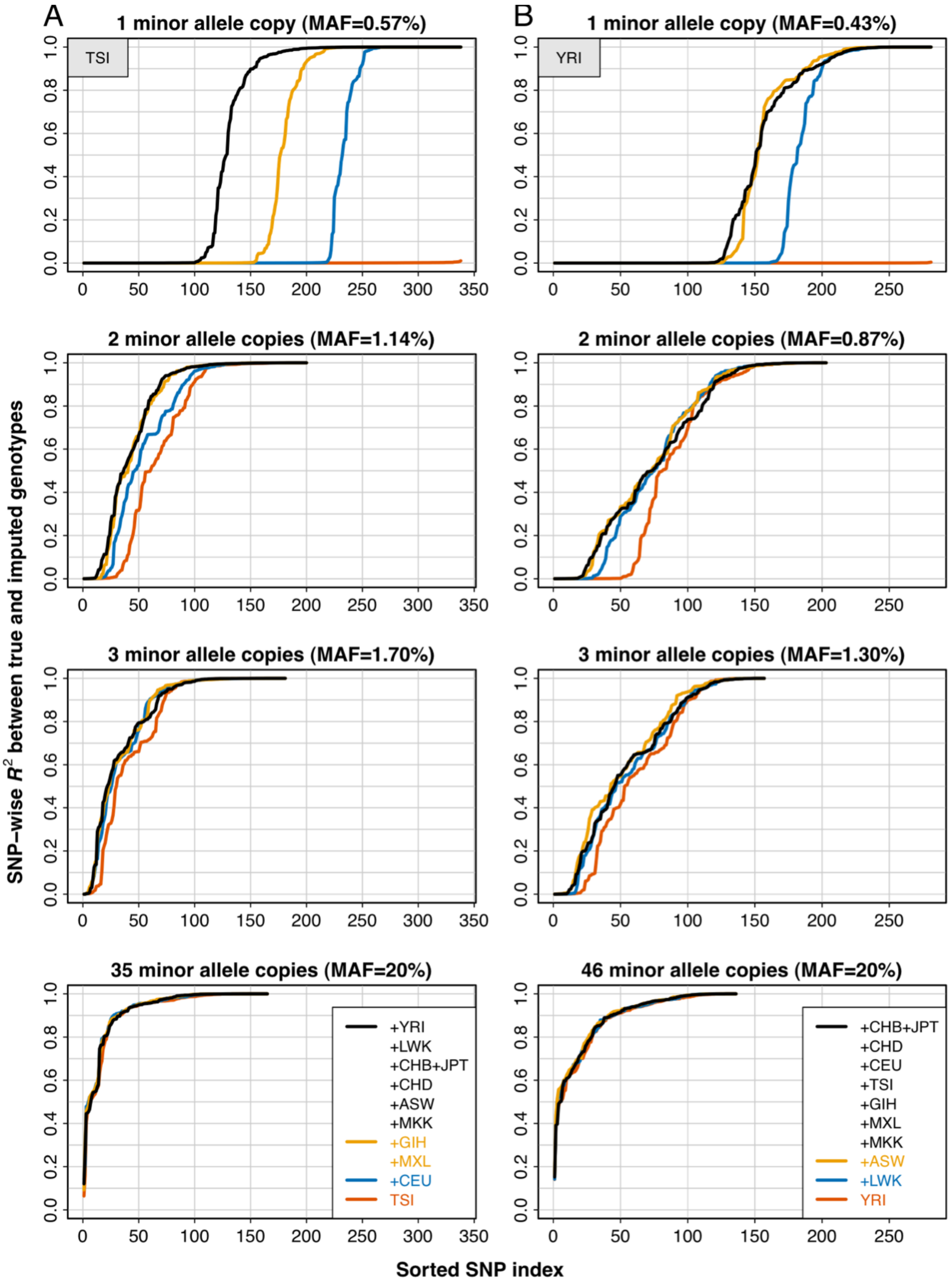

Figure S15 Cumulative distributions of SNP-wise imputation accuracy (R^2) in (A) TSI and (B) YRI, as a function of reference panel composition and minor allele count in the target panel. Further details can be found at the start of this section.
