# Supplementary material for: Genotype Imputation with Thousands of Genomes
Source: G3 (Bethesda). 2011 Nov 1;1(6):457–70. doi: 10.1534/g3.111.001198 (PMC3276165; doi:10.1534/g3.111.001198)
Supplement: Supporting Information [file supp_1.6.457_FileS3.pdf]

**File S3**

**Figures S16-S18**

These figures are similar to Figure 2 of the main text, except they are based on different observed SNP sets (Affymetrix 6.0 or Illumina 1M) and/or imputed SNP MAFs (low-frequency or common), as detailed in the figure captions. The plots show the imputation accuracy of IMPUTE2 and Beagle in all HapMap 3 cross-validation experiments. The accuracy of each experiment is plotted on the *y*-axis as the mean $R^2$ across all imputed SNPs in the specified frequency range in the cross-validation panel (identified by the grey box in each plot). The *x*-axis shows the $k_{hap}$ parameter, which scales linearly with the computational burden of imputation updates in IMPUTE2. The solid black curves show how $R^2$ varies with $k_{hap}$ when using IMPUTE2 with a reference panel containing the full set of 2,020 HapMap 3 haplotypes; the dashed black lines show the accuracy of Beagle with the same reference panel. IMPUTE2 was also applied to sub-panels of the full HapMap 3 panel, with results shown as orange curves.

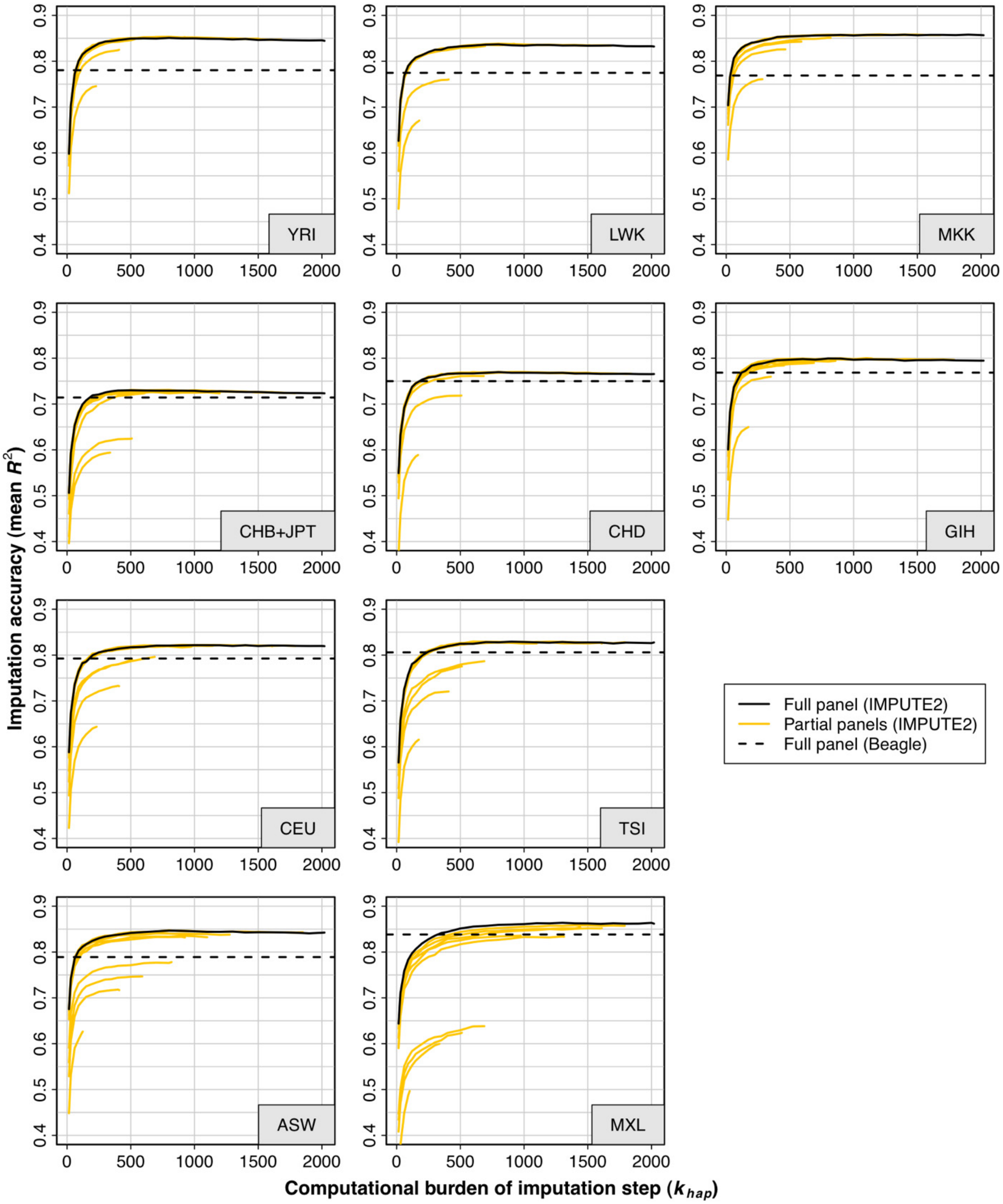


**Figure S16** Imputation accuracy at low-frequency (MAF < 5%) SNPs imputed from observed Illumina 1M genotypes in HapMap 3 cross-validations, as a function of target panel, reference panel composition, $k_{hap}$ value, and imputation method. Further details can be found at the start of this section.

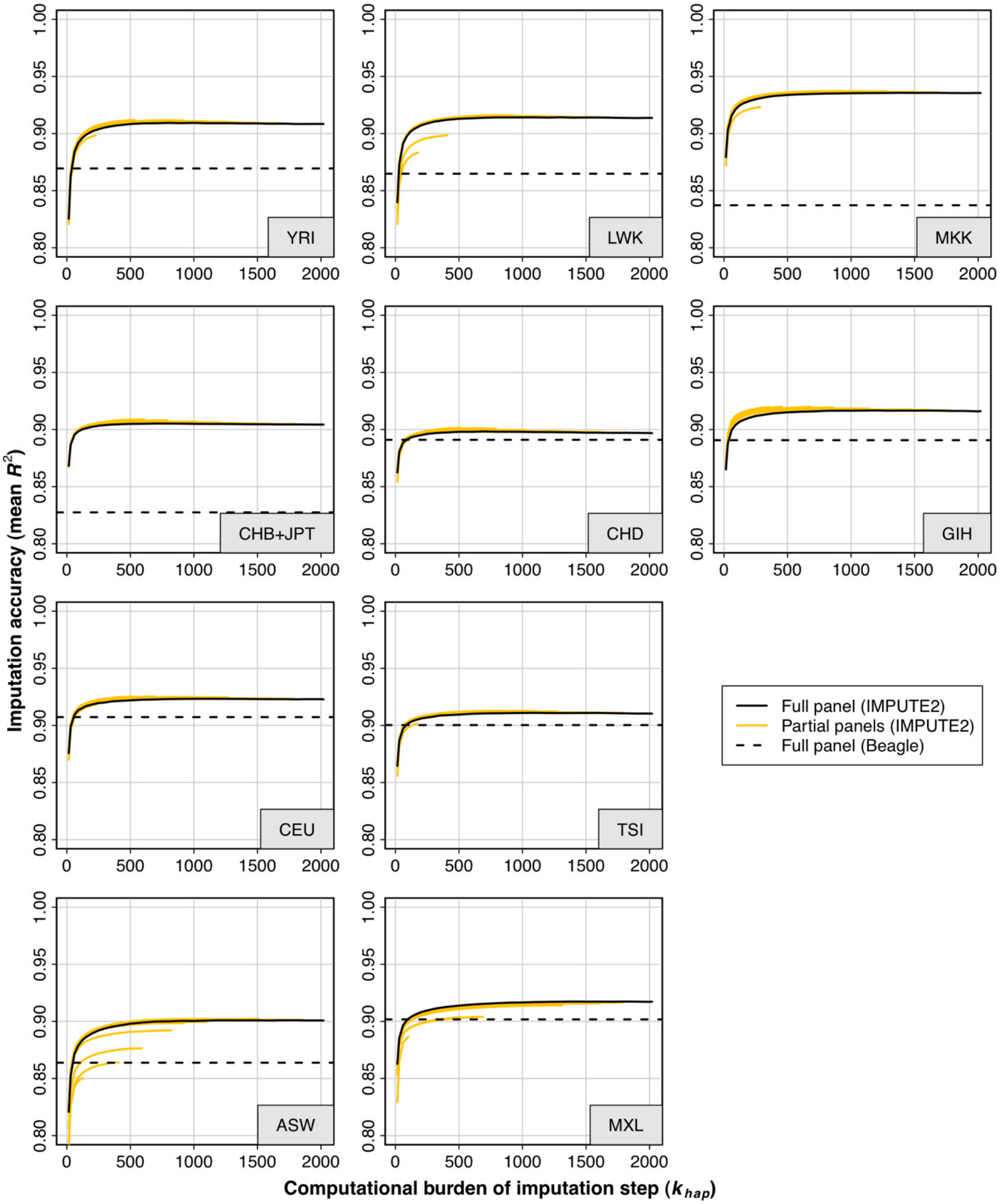


**Figure S17** Imputation accuracy at common (MAF ≥ 5%) SNPs imputed from observed Affymetrix 6.0 genotypes in HapMap 3 cross-validations, as a function of target panel, reference panel composition, $k_{hap}$ value, and imputation method. Further details can be found at the start of this section.

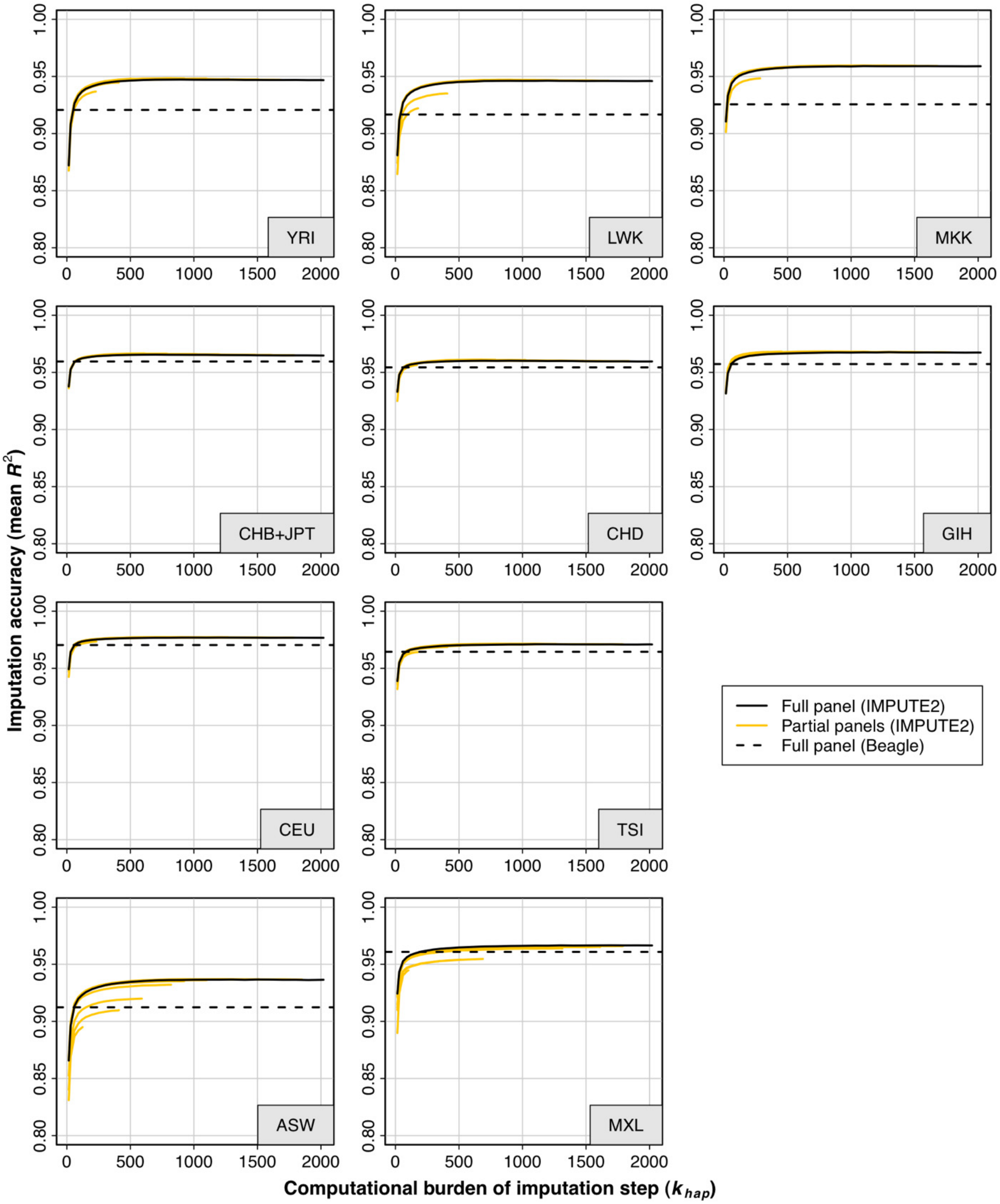


**Figure S18** Imputation accuracy at common (MAF ≥ 5%) SNPs imputed from observed Illumina 1M genotypes in HapMap 3 cross-validations, as a function of target panel, reference panel composition, $k_{hap}$ value, and imputation method. Further details can be found at the start of this section.
