# Supplementary material for: Genotype Imputation with Thousands of Genomes
Source: G3 (Bethesda). 2011 Nov 1;1(6):457–70. doi: 10.1534/g3.111.001198 (PMC3276165; doi:10.1534/g3.111.001198)
Supplement: Supporting Information [file supp_1.6.457_FileS4.pdf]

File S4

DISCREPANCY WITH IMPUTE2 VS. BEAGLE COMPARISON IN JOSTINS ET AL.

One aim of this paper is to compare our imputation framework (as implemented in IMPUTE2) with Beagle, which is another leading method for imputing from large reference panels. We found that IMPUTE2 was more accurate than Beagle in all populations considered. However, as noted in the Results section of the main text, a recent paper by Jostins et al. (2011) reached a different conclusion when comparing these two methods in a similar context. We believe that this discrepancy can be explained by spurious IMPUTE2 results in the Jostins et al. study, as we explain below.

As in our study, Jostins et al. ran IMPUTE2 and Beagle with a cosmopolitan HapMap 3 reference panel. Their cross-validation data comprised ∼1,300 controls sampled from the United Kingdom as part of the Wellcome Trust Case Control Consortium (WTCCC); the closest analogue in our study is the use of the HapMap 3 CEU panel as the target for imputation. Whereas we found that IMPUTE2 was more accurate than Beagle when imputing CEU data from a cosmopolitan HapMap 3 panel, Jostins et al. found that Beagle was more accurate than IMPUTE2 when imputing WTCCC data from the same reference panel: for SNPs with
MAF < 5%, our mean R^2 values were 0.739 and 0.716 for IMPUTE2 and Beagle, respectively, whereas their mean R^2 values were 0.833 and 0.873.

At face value, these results are not necessarily contradictory since they come from different target datasets (WTCCC vs. HapMap 3 CEU) and program settings (Jostins et al. used a smaller value of the *k* parameter in IMPUTE2, which should speed up the program at the cost of imputation accuracy). However, we also noticed other aspects of their results that disagreed with our expectations. For example, Jostins et al. ran IMPUTE version 1 (Marchini et al. 2007) on their data, and they found that it was consistently more accurate than IMPUTE2 (“IMPUTE version 2”), with an especially large difference at low-frequency SNPs. This was true even for ancestrally homogeneous and well-matched reference panels that contained too few haplotypes to activate IMPUTE2’s k_{hap} approximation at the default value of 500.

These results contrast with our long experience as developers of both versions of IMPUTE: in a wide variety of datasets, we have always found that version 2 is slightly more accurate than version 1. The relative accuracies that Jostins et al. obtained for IMPUTE v1 and Beagle fit with our expectations – IMPUTE v1 was somewhat more accurate than Beagle, regardless of reference panel composition – which led us to believe that there might have been an issue with their IMPUTE2 runs.

Fortunately, in this case we had access to the same WTCCC data that Jostins et al. analyzed. To see whether our intuitions were correct, we repeated their imputation experiments as closely as possible (same reference panels, program versions, program settings, target chromosome, and genomic partitioning for parallel runs). Our accuracy numbers were

broadly similar to theirs, with the exception that IMPUTE2 became the most accurate method in almost all scenarios – exactly as we had anticipated.

It is hard to guess the mechanistic reasons for the relatively poor performance of IMPUTE2 in the Jostins et al. study. We downloaded the software from the web, just as any external researcher would do, and it worked well in our hands. Regardless, we believe it is safe to view the IMPUTE2 results from Jostins et al. as an anomaly, whereas the results in this study consistently show that IMPUTE2 is more accurate than Beagle when imputing from large, diverse reference panels.
