# Supplementary material for: Genotype Imputation with Thousands of Genomes
Source: G3 (Bethesda). 2011 Nov 1;1(6):457–70. doi: 10.1534/g3.111.001198 (PMC3276165; doi:10.1534/g3.111.001198)
Supplement: Supporting Information [file supp_1.6.457_FileS5.pdf]

File S5

POTENTIAL PROBLEMS IN MULTI-POPULATION REFERENCE SETS

Here we consider the kinds of problems that could arise from combining reference haplotypes from different populations. In this discussion, we will assume the availability of a reference panel with similar ancestry to the study data, and we will ask whether it would be harmful to augment this panel with haplotypes that are more diverged from the study population. When evaluating whether to include a particular set of haplotypes in the reference panel for a study, one should balance the chances that those haplotypes will contribute shared alleles to the study population against the chances that they will mislead an imputation algorithm. It is difficult to quantify these effects without extensive sequencing or accurate demographic models for the populations in question, but we can still discuss the tradeoffs between useful and misleading haplotypes in general terms.

Intuitively, allele sharing should decrease as the candidate haplotypes grow more diverged from the study population. Highly diverged reference haplotypes are easily identified and discarded by imputation models, so while they are unlikely to help imputation accuracy, they are also unlikely to hurt it. On the other hand, reference haplotypes that are closely related to the study population have the capacity to both help and mislead imputation methods. There are a few kinds of sites at which a set of candidate reference haplotypes could be misleading when added to a well-matched panel:

1. Sites that are monomorphic in the study dataset but polymorphic in the reference haplotypes. These could cause the imputation of alleles that do not exist in the study data.
2. Sites that are polymorphic in the study dataset but monomorphic in the reference haplotypes. These could dilute the imputation signal if the haplotype background of the variant allele is present in the reference set.
3. Sites that have experienced recurrent mutation, such that the study population and reference haplotypes carry the same variant allele on different haplotype backgrounds. These could cause imputed false alleles, missed true alleles, or low-confidence imputation, depending on the relative frequencies of the mutations.

The prevalence of these scenarios will depend on a complex mixture of factors such as sample size, allele frequency, divergence time, migration rates, and properties of the SNPs typed in the study individuals. We can evaluate the respective scenarios in light of their potential effects on power in an association study:

1. Imputed, non-existent alleles clearly do not increase power, but we argue that they are also unlikely to create

false associations. For this to happen, the variant alleles in the reference panel would have to correlate strongly with the phenotype in an association study after applying standard stratification corrections, and this seems unlikely to happen in a well-designed study. We looked at these kinds of sites in our HapMap 3 cross-validations, and we found that variant alleles were almost never imputed into panels that did not carry those alleles (data not shown). Hence, this class of sites neither helps nor hurts the association testing.

2. Diluted imputation signals can hurt the power to detect associations at these sites. Signal dilution is most likely to occur in closely related populations that share haplotype backgrounds and that lack the flanking mutations to distinguish these backgrounds. (Note that "distinguishing mutations" must be assayed on a study's genotyping platform if they are to discriminate similar haplotype backgrounds.) Fortunately, this effect is self-limiting since the more closely two populations are related, the rarer an allele would tend to be if it were present in one population but not the other. We hypothesize that these kinds of alleles will typically be too rare to have phenotypic effects that are detectable in single-marker association tests.
3. Recurrent mutations should cause much the same effects as the diluted imputation signals discussed above, and they should be subject to the same frequency limitations. Previous work has shown that of the alleles which occur at low frequency in multiple populations, only a small fraction show evidence of recurrent mutation (The International HapMap Consortium 2010).

While quantifying these statements is an important and open area of research, we expect that the worldwide reference set being collected by the 1,000 Genomes Project will be mostly immune to these problems, which motivates our suggestion to use inclusive reference sets from that resource. Rare genomic phenomena (e.g., the well-known positive selection on recurrent mutations in the beta-globin gene) will create occasional exceptions to the trends discussed here, but the regions that could harbor these kinds of events can probably be identified ahead of time and given special treatment. We also note that negative effects of misleading haplotypes will be mitigated by the continuing development of SNP arrays targeted at low-frequency variation. Looking ahead, it will be important to reevaluate the merits of combining panels in future reference datasets, including dedicated reference data collected in fine-mapping experiments or exome sequencing studies.
