# Supplementary material for: Genotype Imputation with Thousands of Genomes
Source: G3 (Bethesda). 2011 Nov 1;1(6):457–70. doi: 10.1534/g3.111.001198 (PMC3276165; doi:10.1534/g3.111.001198)
Supplement: Supporting Information [file supp_1.6.457_FileS6.pdf]

File S6

RELATIONSHIP BETWEEN k_{hap} AND A COALESCENT-BASED APPROXIMATION

While we were preparing this manuscript for publication, another group published a method for choosing a custom reference panel for each study individual in each part of the genome (Pasaniuc et al. 2010). Like our k_{hap} approximation, their "coalescent-based" approach is motivated by genealogical reasoning, and the methods share other features: they both eliminate reference haplotypes that differ by too many alleles from a study haplotype of interest, and they are both designed to handle multi-population reference panels and admixed individuals.

However, there are important differences in the motivations for these approximations. Aside from accounting for changes in local ancestry, the other main goal of the Pasaniuc et al. method is to increase imputation accuracy by re-weighting the reference haplotypes with probabilities derived from coalescent theory. Their method essentially looks at the data and then modifies the probability that a given study individual will copy each reference haplotype; reference haplotypes that show high identity with the study genotypes are upweighted, thereby increasing the chances that an imputation method will copy them. IMPUTE2 also uses coalescent arguments to weight reference haplotypes, but in our case the weights are built into the underlying statistical model (which was originally described by Li and Stephens (2003)) and can be obtained with a uniform prior on copying each reference haplotype. Our k_{hap} approximation can be seen as changing these weights, in the sense that it rounds to zero the copying probabilities of haplotypes separated by many allele differences, but it assumes a uniform prior probability of copying the remaining k_{hap} haplotypes. This approximation does not aim to change the haplotype weights; its goal is to maintain the relative weights under the model while avoiding expensive HMM calculations on reference haplotypes that will contribute little to the genotype imputation probabilities.

Fundamentally, the Pasaniuc et al. approximation is designed to increase imputation accuracy (usually at increased computational cost), whereas our approximation is designed to maintain accuracy while reducing computation (although, as we saw in the Results, it may slightly improve accuracy in some situations). For investigators running imputation methods, accuracy and efficiency are competing demands. Imputing a large GWAS requires substantial computing power, so any method that claims to increase accuracy must produce large enough gains to justify the computational cost.

To assess this tradeoff, we implemented the Pasaniuc et al. method and tried it with various settings in our HapMap 3 comparisons. As suggested, we used their approximation to create local reference panels for each study individual and reference SNP, then ran IMPUTE2 with no k_{hap} restriction. Our preliminary experiments showed that the haplotype re-weighting scheme was both slower (as expected) *and* less accurate than IMPUTE2 on default settings. This was true even for admixed datasets, where local re-weighting is meant to excel. On the basis of these initial results, we decided not to pursue the Pasaniuc

et al. method further.

We acknowledge that the Pasaniuc et al. approximation could, in principle, improve accuracy by introducing a notion of time depth in the coalescent process; Stephens and Scheet (2005) used a similar idea to develop an extension of the Li and Stephens model. Hence, the poor performance of the Pasaniuc et al. approximation in our experiments could reflect a failure to locate the optimal parameters of the model, which might depend on reference panel size, the SNP density and content of a GWAS dataset, the allele frequency of the SNP being imputed, and other factors. Alternatively, our results could imply that the method inherently works less well in reference panels like HapMap 3 than in the smaller panels on which it was originally tested, as was recently suggested by some of the authors of the Pasaniuc et al. method (Pasaniuc et al. 2011).

Regardless of the true reasons for these results, we find it instructive that we, as well-informed users, could not get the Pasaniuc et al. method to produce high accuracy on our data. Perhaps future developments will make the method easier to use in a variety of situations, but in the meantime we suggest that the k_{hap} approximation within IMPUTE2 offers many practical advantages for GWAS investigators.

REFERENCES

1. L. Jostins, K. I. Morley, and J. C. Barrett. Imputation of low-frequency variants using the HapMap3 benefits from large, diverse reference sets. *Eur. J. Hum. Genet.*, 19:662–666, Jun 2011.
2. Na Li and Matthew Stephens. Modeling linkage disequilibrium and identifying recombination hotspots using single-nucleotide polymorphism data. *Genetics*, 165(4):2213–2233, Dec 2003.
3. B. Pasaniuc, R. Avinery, T. Gur, C. F. Skibola, P. M. Bracci, and E. Halperin. A generic coalescent-based framework for the selection of a reference panel for imputation. *Genet. Epidemiol.*, 34: 773–782, Dec 2010.
4. B. Pasaniuc, N. Zaitlen, G. Lettre, G. K. Chen, A. Tandon, W. H. Kao, I. Ruczinski, M. Fornage, D. S. Siscovick, X. Zhu, et al. Enhanced Statistical Tests for GWAS in Admixed Populations: Assessment using African Americans from CARe and a Breast Cancer Consortium. *PLoS Genet.*, 7:e1001371, Apr 2011.
5. Matthew Stephens and Paul Scheet. Accounting for decay of linkage disequilibrium in haplotype inference and missing-data imputation. *Am J Hum Genet*, 76(3):449–462, Mar 2005.
6. The International HapMap Consortium. Integrating common and rare genetic variation in diverse human populations. *Nature*, 467:52–58, Sep 2010.
