# Supplementary material for: Genotype Imputation with Thousands of Genomes
Source: G3 (Bethesda). 2011 Nov 1;1(6):457–70. doi: 10.1534/g3.111.001198 (PMC3276165; doi:10.1534/g3.111.001198)
Supplement: Supporting Information [file supp_1.6.457_TableS1.pdf]

Table S1   Number of low-frequency SNPs imputed in each HapMap 3 panel from Affymetrix 6.0 SNPs

Panel	Number of SNPs with MAF < 5%
ASW	1870
CEU	1579
CHB+JPT	2206
CHD	1882
GIH	1667
LWK	2230
MKK	1925
MXL	2364
TSI	1523
YRI	1844
