# Supplementary material for: Genotype Imputation with Thousands of Genomes
Source: G3 (Bethesda). 2011 Nov 1;1(6):457–70. doi: 10.1534/g3.111.001198 (PMC3276165; doi:10.1534/g3.111.001198)
Supplement: Supporting Information [file supp_1.6.457_TableS2.pdf]

Table S2 Discovery probabilities for SNPs by variant allele count in simulated reference panels

Variant allele count of SNP in reference set	Probability of discovery
1	0.25
2	0.50
3	0.74
4	0.87
5	0.90
6	0.93
7	0.97
8	0.98
9	0.98
10+	0.99
